# A step-by-step workflow for low-level analysis of single-cell RNA-seq data with Bioconductor

**DOI:** 10.12688/f1000research.9501.2

**Published:** 2016-10-31

**Authors:** Aaron T.L. Lun, Davis J. McCarthy, John C. Marioni

**Affiliations:** 1 Cancer Research UK Cambridge Institute, Cambridge, UK; 2 EMBL European Bioinformatics Institute, Cambridge, UK; 3 St Vincent’s Institute of Medical Research, Fitzroy, Australia; 4 Wellcome Trust Sanger Institute, Cambridge, UK

**Keywords:** Single cell, RNA-seq, bioinformatics, Bioconductor, workflow

## Abstract

Single-cell RNA sequencing (scRNA-seq) is widely used to profile the transcriptome of individual cells. This provides biological resolution that cannot be matched by bulk RNA sequencing, at the cost of increased technical noise and data complexity. The differences between scRNA-seq and bulk RNA-seq data mean that the analysis of the former cannot be performed by recycling bioinformatics pipelines for the latter. Rather, dedicated single-cell methods are required at various steps to exploit the cellular resolution while accounting for technical noise. This article describes a computational workflow for low-level analyses of scRNA-seq data, based primarily on software packages from the open-source Bioconductor project. It covers basic steps including quality control, data exploration and normalization, as well as more complex procedures such as cell cycle phase assignment, identification of highly variable and correlated genes, clustering into subpopulations and marker gene detection. Analyses were demonstrated on gene-level count data from several publicly available datasets involving haematopoietic stem cells, brain-derived cells, T-helper cells and mouse embryonic stem cells. This will provide a range of usage scenarios from which readers can construct their own analysis pipelines.

## Introduction

Single-cell RNA sequencing (scRNA-seq) is widely used to measure the genome-wide expression profile of individual cells. From each cell, mRNA is isolated and reverse transcribed to cDNA for high-throughput sequencing ( Stegle *et al.*, 2015). This can be done using microfluidics platforms like the Fluidigm C1 ( Pollen *et al.*, 2014), protocols based on microtiter plates like Smart-seq2 ( Picelli *et al.*, 2014), or droplet-based technologies like inDrop ( Klein *et al.*, 2015; Macosko *et al.*, 2015). The number of reads mapped to each gene is then used to quantify its expression in each cell. Alternatively, unique molecular identifiers (UMIs) can be used to directly measure the number of transcript molecules for each gene ( Islam *et al.*, 2014). Count data are analyzed to detect highly variable genes (HVGs) that drive heterogeneity across cells in a population, to find correlations between genes and cellular phenotypes, or to identify new subpopulations via dimensionality reduction and clustering. This provides biological insights at a single-cell resolution that cannot be achieved with conventional bulk RNA sequencing of cell populations.

Strategies for scRNA-seq data analysis differ markedly from those for bulk RNA-seq. One technical reason is that scRNA-seq data are much noisier than bulk data ( Brennecke *et al.*, 2013; Marinov *et al.*, 2014). Reliable capture (i.e., conversion) of transcripts into cDNA for sequencing is difficult with the low quantity of RNA in a single cell. This increases the frequency of drop-out events where none of the transcripts for a gene are captured. Dedicated steps are required to deal with this noise during analysis, especially during quality control. In addition, scRNA-seq data can be used to study cell-to-cell heterogeneity, e.g., to identify new cell subtypes, to characterize differentiation processes, to assign cells into their cell cycle phases, or to identify HVGs driving variability across the population ( Fan *et al.*, 2016; Trapnell *et al.*, 2014; Vallejos *et al.*, 2015). This is simply not possible with bulk data, meaning that custom methods are required to perform these analyses.

This article describes a computational workflow for basic analysis of scRNA-seq data, using software packages from the open-source Bioconductor project (release 3.4) ( Huber *et al.*, 2015). Starting from a count matrix, this workflow contains the steps required for quality control to remove problematic cells; normalization of cell-specific biases, with and without spike-ins; cell cycle phase classification from gene expression data; data exploration to identify putative subpopulations; and finally, HVG and marker gene identification to prioritize interesting genes. The application of different steps in the workflow will be demonstrated on several public scRNA-seq datasets involving haematopoietic stem cells, brain-derived cells, T-helper cells and mouse embryonic stem cells, generated with a range of experimental protocols and platforms ( Buettner *et al.*, 2015; Kolodziejczyk *et al.*, 2015; Wilson *et al.*, 2015; Zeisel *et al.*, 2015). The aim is to provide a variety of modular usage examples that can be applied to construct custom analysis pipelines.

## Analysis of haematopoietic stem cells

### Overview

To introduce most of the concepts of scRNA-seq data analysis, we use a relatively simple dataset from a study of haematopoietic stem cells (HSCs) ( Wilson *et al.*, 2015). Single mouse HSCs were isolated into microtiter plates and libraries were prepared for 96 cells using the Smart-seq2 protocol. A constant amount of spike-in RNA from the External RNA Controls Consortium (ERCC) was also added to each cell’s lysate prior to library preparation. High-throughput sequencing was performed and the expression of each gene was quantified by counting the total number of reads mapped to its exonic regions. Similarly, the quantity of each spike-in transcript was measured by counting the number of reads mapped to the spike-in reference sequences. Counts for all genes/transcripts in each cell were obtained from the NCBI Gene Expression Omnibus (GEO) as a supplementary file under the accession number GSE61533 ( http://www.ncbi.nlm.nih.gov/geo/query/acc.cgi?acc=GSE61533).

For simplicity, we forego a description of the read processing steps required to generate the count matrix, i.e., read alignment and counting into features. These steps have been described in some detail elsewhere ( Chen *et al.*, 2016; Love *et al.*, 2015), and are largely the same for bulk and single-cell data. The only additional consideration is that the spike-in information must be included in the pipeline. Typically, spike-in sequences can be included as additional FASTA files during genome index building prior to alignment, while genomic intervals for both spike-in transcripts and endogenous genes can be concatenated into a single GTF file prior to counting. For users favouring an R-based approach to read alignment and counting, we suggest using the methods in the *Rsubread* package ( Liao *et al.*, 2013; Liao *et al.*, 2014). Alternatively, rapid quantification of expression with alignment-free methods such as *kallisto* ( Bray *et al.*, 2016) or *Salmon* ( Patro *et al.*, 2015) can be performed using the functions runKallisto and runSalmon in the *scater* package.

### Count loading

The first task is to load the count matrix into memory. In this case, some work is required to retrieve the data from the Gzip-compressed Excel format. Each row of the matrix represents an endogenous gene or a spike-in transcript, and each column represents a single HSC. For convenience, the counts for spike-in transcripts and endogenous genes are stored in a SCESet object from the *scater* package ( McCarthy *et al.*, 2016).



                        library
                        (R.utils)

                        gunzip
                        (
                        "GSE61533_HTSEQ_count_results.xls.gz"
                        , 
                        remove=
                        FALSE
                        , 
                        overwrite=
                        TRUE
                        )

                        library
                        (gdata)

                        all.counts <- 
                        read.xls
                        (
                        ’GSE61533_HTSEQ_count_results.xls’
                        , 
                        sheet=
                        1
                        , 
                        header=
                        TRUE
                        , 
                        row.names=
                        1
                        )

                        library
                        (scater)

                        sce <- 
                        newSCESet
                        (
                        countData=
                        all.counts)

                        dim
                        (sce)
                    




                        ## Features Samples
##    38498      96
                    


We identify the rows corresponding to ERCC spike-ins and mitochondrial genes. For this dataset, this information can be easily extracted from the row names. In general, though, identifying mitochondrial genes from standard identifiers like Ensembl requires extra annotation (this will be discussed later in more detail).



                        is.spike <- 
                        grepl
                        (
                        "^ERCC"
                        , 
                        rownames
                        (sce))

                        is.mito <- 
                        grepl
                        (
                        "^mt-"
                        , 
                        rownames
                        (sce))
                    


For each cell, we calculate quality control metrics such as the total number of counts or the proportion of counts in mitochondrial genes or spike-in transcripts. These are stored in the pData of the SCESet for future reference.



                        sce <- 
                        calculateQCMetrics
                        (sce, 
                        feature_controls=list
                        (
                        ERCC=
                        is.spike, 
                        Mt=
                        is.mito))

                        head
                        (
                        colnames
                        (
                        pData
                        (sce)))
                    




                        ## [1] "total_counts"          "log10_total_counts"       "filter_on_total_counts"
## [4] "total_features"        "log10_total_features"     "filter_on_total_features"
                    


We need to explicitly indicate that the ERCC set is, in fact, a spike-in set. This is necessary as spike-ins require special treatment in some downstream steps such as variance estimation and normalization. We do this by supplying the name of the spike-in set to isSpike.



                        library
                        (scran)

                        isSpike
                        (sce) <- 
                        "ERCC"
                    


### Quality control on the cells

**Figure 1.**
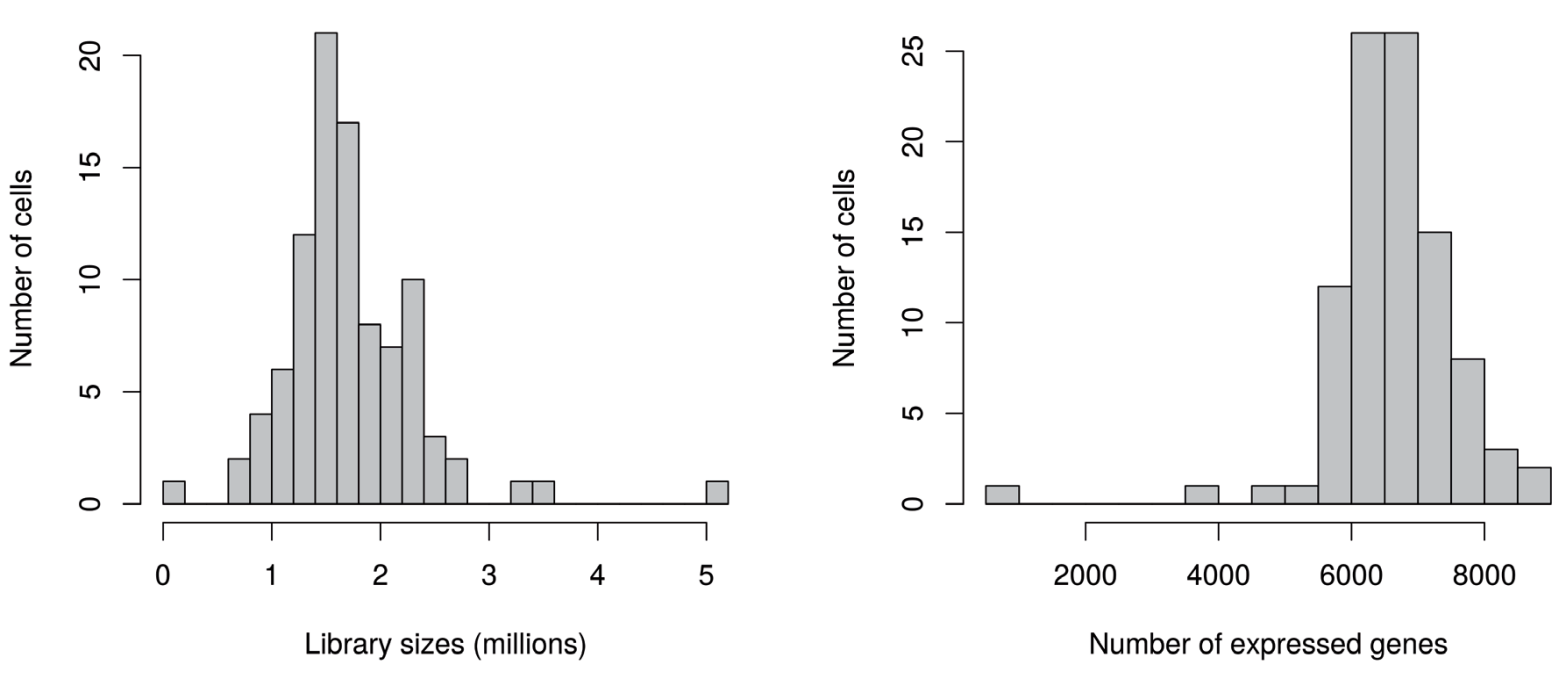
Histograms of library sizes (left) and number of expressed genes (right) for all cells in the HSC dataset.



Low-quality cells need to be removed to ensure that technical effects do not distort downstream analysis results. Two common measures of cell quality are the library size and the number of expressed features in each library. The library size is defined as the total sum of counts across all features, i.e., genes and spike-in transcripts. Cells with relatively small library sizes are considered to be of low quality as the RNA has not been efficiently captured (i.e., converted into cDNA and amplified) during library preparation. The number of expressed features in each cell is defined as the number of features with non-zero counts for that cell. Any cell with very few expressed genes is likely to be of poor quality as the diverse transcript population has not been successfully captured. The distributions of both of these metrics are shown in Figure 1.

                        par
                        (
                        mfrow=c
                        (
                        1
                        ,
                        2
                        ))

                        hist
                        (sce$total_counts/
                        1e6
                        , 
                        xlab=
                        "Library sizes (millions)"
                        , 
                        main=
                        ""
                        ,
     
                        breaks=
                        20
                        , 
                        col=
                        "grey80"
                        , 
                        ylab=
                        "Number of cells"
                        )

                        hist
                        (sce$total_features, 
                        xlab=
                        "Number of expressed genes"
                        , 
                        main=
                        ""
                        ,
     
                        breaks=
                        20
                        , 
                        col=
                        "grey80"
                        , 
                        ylab=
                        "Number of cells"
                        )
                    


Picking a threshold for these metrics is not straightforward as their absolute values depend on the protocol and biological system. For example, sequencing to greater depth will lead to more reads, regardless of the quality of the cells. To obtain an adaptive threshold, we assume that most of the dataset consists of high-quality cells. We remove cells with log-library sizes that are more than 3 median absolute deviations (MADs) below the median log-library size. (A log-transformation improves resolution at small values, especially when the MAD of the raw values is comparable to or greater than the median.) We also remove cells where the log-transformed number of expressed genes is 3 MADs below the median.



                        libsize.drop <- 
                        isOutlier
                        (sce$total_counts, 
                        nmads=
                        3
                        , 
                        type=
                        "lower"
                        , 
                        log=
                        TRUE
                        )

                        feature.drop <- 
                        isOutlier
                        (sce$total_features, 
                        nmads=
                        3
                        , 
                        type=
                        "lower"
                        , 
                        log=
                        TRUE
                        )
                    


**Figure 2.**
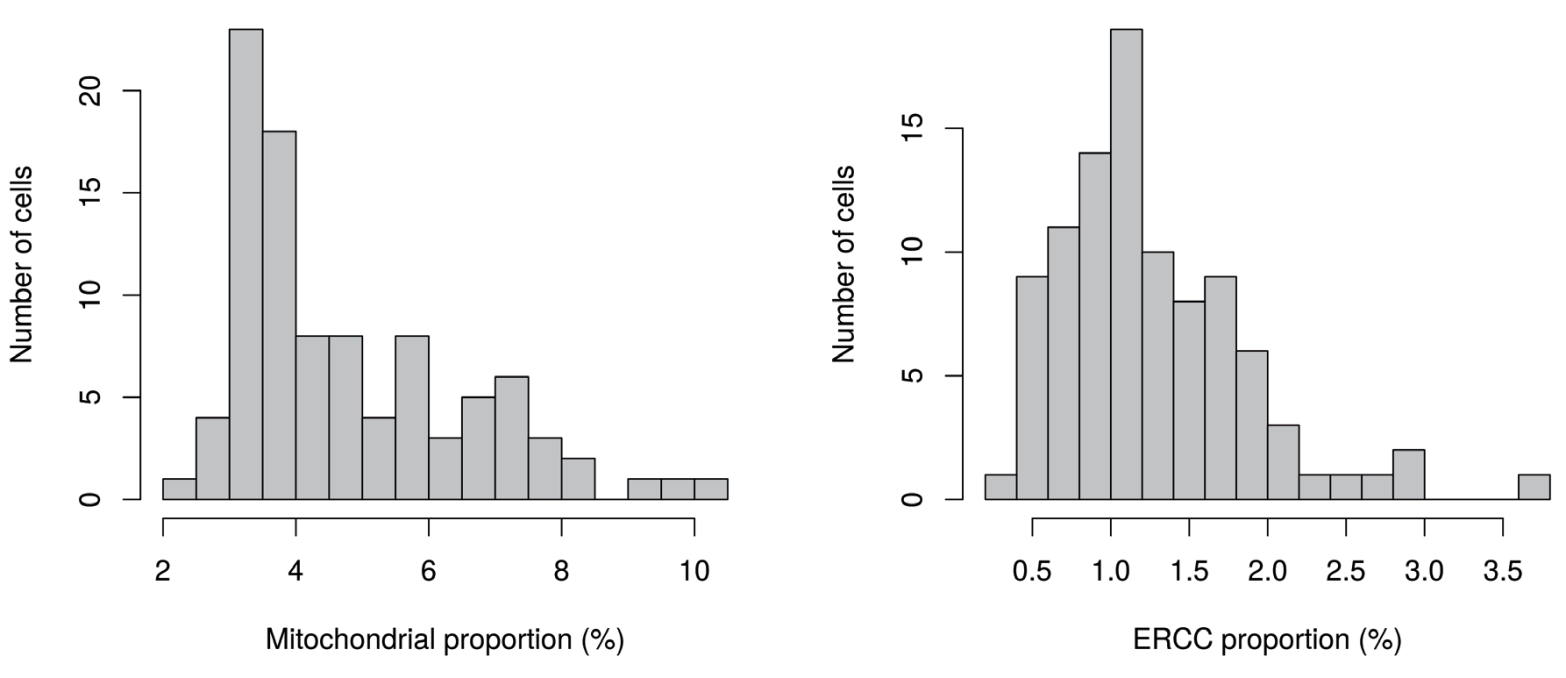
Histogram of the proportion of reads mapped to mitochondrial genes (left) or spike-in transcripts (right) across all cells in the HSC dataset.



Another measure of quality is the proportion of reads mapped to genes in the mitochondrial genome. High proportions are indicative of poor-quality cells ( Ilicic *et al.*, 2016; Islam *et al.*, 2014), possibly because of increased apoptosis and/or loss of cytoplasmic RNA from lysed cells. Similar reasoning applies to the proportion of reads mapped to spike-in transcripts. The quantity of spike-in RNA added to each cell should be constant, which means that the proportion should increase upon loss of endogenous RNA in low-quality cells. The distributions of mitochondrial and spike-in proportions across all cells are shown in Figure 2.

                        par
                        (
                        mfrow=c
                        (
                        1
                        ,
                        2
                        ))

                        hist
                        (sce$pct_counts_feature_controls_Mt, 
                        xlab=
                        "Mitochondrial proportion (%)"
                        ,
     
                        ylab=
                        "Number of cells"
                        , 
                        breaks=
                        20
                        , 
                        main=
                        ""
                        , 
                        col=
                        "grey80"
                        )

                        hist
                        (sce$pct_counts_feature_controls_ERCC, 
                        xlab=
                        "ERCC proportion (%)"
                        ,
     
                        ylab=
                        "Number of cells"
                        , 
                        breaks=
                        20
                        , 
                        main=
                        ""
                        , 
                        col=
                        "grey80"
                        )
                    


Again, the ideal threshold for these proportions depends on the cell type and the experimental protocol. Cells with more mitochondria or more mitochondrial activity may naturally have larger mitochondrial proportions. Similarly, cells with more endogenous RNA or that are assayed with protocols using less spike-in RNA will have lower spike-in proportions. If we assume that most cells in the dataset are of high quality, then the threshold can be set to remove any large outliers from the distribution of proportions. We use the MAD-based definition of outliers to remove putative low-quality cells from the dataset.



                        mito.drop <- 
                        isOutlier
                        (sce$pct_counts_feature_controls_Mt, 
                        nmads=
                        3
                        , 
                        type=
                        "higher"
                        )

                        spike.drop <- 
                        isOutlier
                        (sce$pct_counts_feature_controls_ERCC, 
                        nmads=
                        3
                        , 
                        type=
                        "higher"
                        )
                    


Subsetting by column will retain only the high-quality cells that pass each filter described above. We examine the number of cells removed by each filter as well as the total number of retained cells. Removal of a substantial proportion of cells (> 10%) may be indicative of an overall issue with data quality. It may also reflect genuine biology in extreme cases (e.g., low numbers of expressed genes in erythrocytes) for which the filters described here are inappropriate.



                        sce <- sce[,!(libsize.drop | feature.drop | mito.drop | spike.drop)]

                        data.frame
                        (
                        ByLibSize=sum
                        (libsize.drop), 
                        ByFeature=sum
                        (feature.drop),
     
                        ByMito=sum
                        (mito.drop), 
                        BySpike=sum
                        (spike.drop), 
                        Remaining=ncol
                        (sce))
                    




                        ##         ByLibSize ByFeature ByMito BySpike Remaining
## Samples         2         2      6       3        86
                    


**Figure 3.**
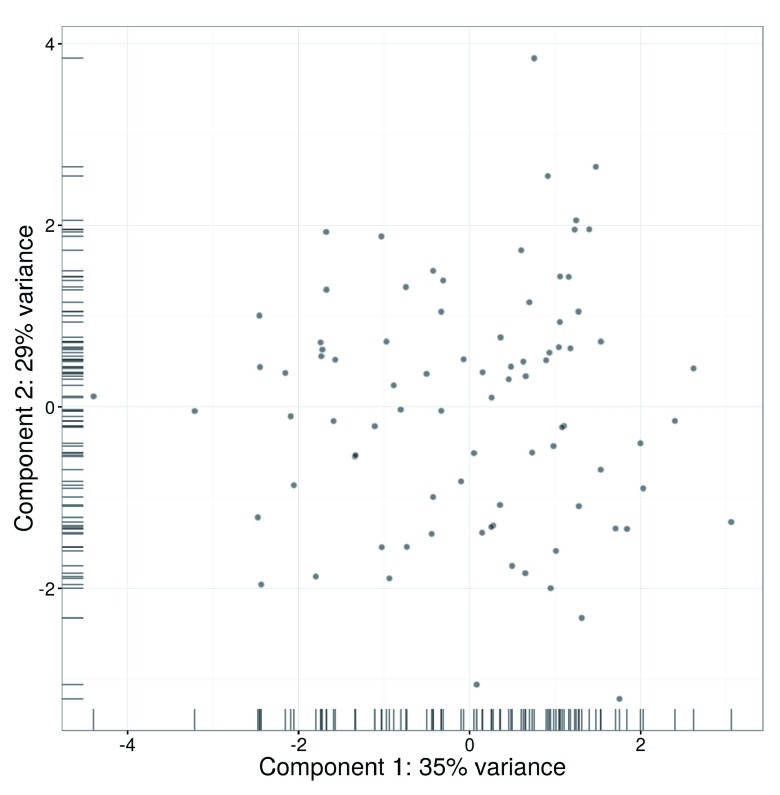
PCA plot for cells in the HSC dataset, constructed using quality metrics. The first and second components are shown on each axis, along with the percentage of total variance explained by each component. Bars represent the coordinates of the cells on each axis.



An alternative approach to quality control is to perform a principal components analysis (PCA) based on the quality metrics for each cell, e.g., the total number of reads, the total number of features and the proportion of mitochondrial or spike-in reads. Outliers on a PCA plot may be indicative of low-quality cells that have aberrant technical properties compared to the (presumed) majority of high-quality cells. In Figure 3, no obvious outliers are present, which is consistent with the removal of suspect cells in the preceding quality control steps.

                        fontsize <- 
                        theme
                        (
                        axis.text=element_text
                        (
                        size=
                        12
                        ), 
                        axis.title=element_text
                        (
                        size=
                        16
                        ))

                        plotPCA
                        (sce, 
                        pca_data_input=
                        "pdata"
                        ) + fontsize
                    


Methods like PCA-based outlier detection and support vector machines can provide more power to distinguish low-quality cells from high-quality counterparts ( Ilicic *et al.*, 2016). This is because they are able to detect subtle patterns across many quality metrics simultaneously. However, this comes at some cost to interpretability, as the reason for removing a given cell may not always be obvious. Thus, for this workflow, we will use the simple approach whereby each quality metric is considered separately. Users interested in the more sophisticated approaches are referred to the *scater* and *cellity* packages.

### Classification of cell cycle phase

**Figure 4.**
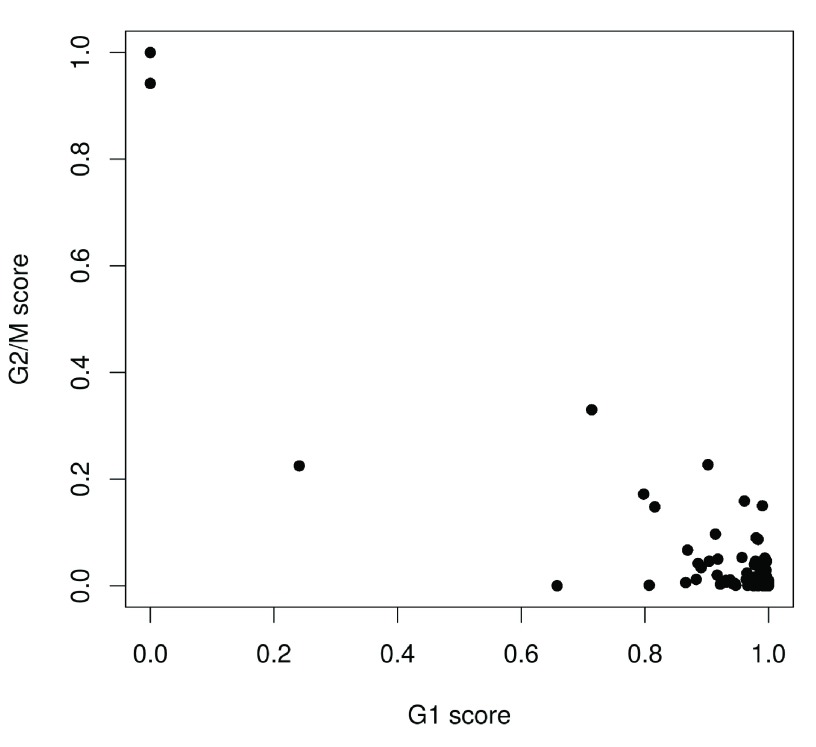
Cell cycle phase scores from applying the pair-based classifier on the HSC dataset, where each point represents a cell.



We use the prediction method described by Scialdone *et al.* (2015) to classify cells into cell cycle phases based on the gene expression data. Using a training dataset, the sign of the difference in expression between two genes was computed for each pair of genes. Pairs with changes in the sign across cell cycle phases were chosen as markers. Cells in a test dataset can then be classified into the appropriate phase, based on whether the observed sign for each marker pair is consistent with one phase or another. This approach is implemented in the cyclone function using a pre-trained set of marker pairs for mouse data. The result of phase assignment for each cell in the HSC dataset is shown in Figure 4. (Some additional work is necessary to match the gene symbols in the data to the Ensembl annotation in the pre-trained marker set.)

                        mm.pairs <- 
                        readRDS
                        (
                        system.file
                        (
                        "exdata"
                        , 
                        "mouse_cycle_markers.rds"
                        , 
                        package=
                        "scran"
                        ))

                        library
                        (org.Mm.eg.db)

                        anno <- 
                        select
                        (org.Mm.eg.db, 
                        keys=rownames
                        (sce), 
                        keytype=
                        "SYMBOL"
                        , 
                        column=
                        "ENSEMBL"
                        )
ensembl <- anno$ENSEMBL[
                        match
                        (
                        rownames
                        (sce), anno$SYMBOL)]
assignments <- 
                        cyclone
                        (sce, mm.pairs, 
                        gene.names=
                        ensembl)

                        plot
                        (assignments$score$G1, assignments$score$G2M, 
                        xlab=
                        "G1 score"
                        , 
                        ylab=
                        "G2/M score"
                        , 
                        pch=
                        16
                        )
                    


Cells are classified as being in G1 phase if the G1 score is above 0.5 and greater than the G2/M score; in G2/M phase if the G2/M score is above 0.5 and greater than the G1 score; and in S phase if neither score is above 0.5. Here, the vast majority of cells are classified as being in G1 phase. We will focus on these cells in the downstream analysis. Cells in other phases are removed to avoid potential confounding effects from cell cycle-induced differences. Alternatively, if a non-negligible number of cells are in other phases, we can use the assigned phase as a blocking factor in downstream analyses. This protects against cell cycle effects without discarding information.



                        sce <- sce[,assignments$phases==
                        "G1"
                        ]
                    


Pre-trained classifiers are available in *scran* for human and mouse data. While the mouse classifier used here was trained on data from embryonic stem cells, it is still accurate for other cell types ( Scialdone *et al.*, 2015). This may be due to the conservation of the transcriptional program associated with the cell cycle ( Bertoli *et al.*, 2013; Conboy *et al.*, 2007). The pair-based method is also a non-parametric procedure that is robust to most technical differences between datasets. However, it will be less accurate for data that are substantially different from those used in the training set, e.g., due to the use of a different protocol. In such cases, users can construct a custom classifier from their own training data using the sandbag function. This will also be necessary for other model organisms where pre-trained classifiers are not available.

### Filtering out low-abundance genes

Low-abundance genes are problematic as zero or near-zero counts do not contain enough information for reliable statistical inference ( Bourgon *et al.*, 2010). In addition, the discreteness of the counts may interfere with downstream statistical procedures, e.g., by compromising the accuracy of continuous approximations. Here, low-abundance genes are defined as those with an average count below a filter threshold of 1. These genes are likely to be dominated by drop-out events ( Brennecke *et al.*, 2013), which limits their usefulness in later analyses. Removal of these genes mitigates discreteness and reduces the amount of computational work without major loss of information.



                        ave.counts <- 
                        rowMeans
                        (
                        counts
                        (sce))

                        keep <- ave.counts >= 
                        1

                        sum
                        (keep)
                    




                        ## [1] 13965
                    


**Figure 5.**
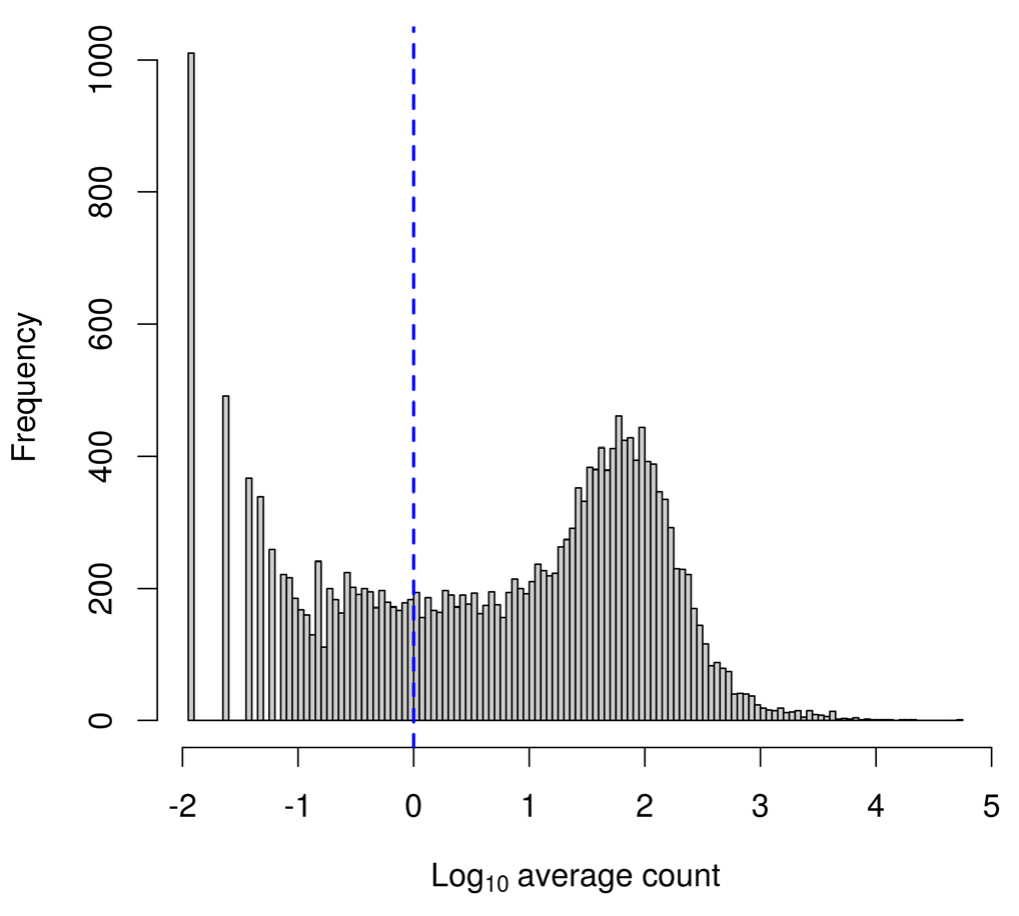
Histogram of log-average counts for all genes in the HSC dataset. The filter threshold is represented by the blue line.



To check whether the chosen threshold is suitable, we examine the distribution of log-means across all genes ( Figure 5). The peak represents the bulk of moderately expressed genes while the rectangular component corresponds to lowly expressed genes. The filter threshold should cut the distribution at some point along the rectangular component to remove the majority of low-abundance genes.

                        hist
                        (
                        log10
                        (ave.counts), 
                        breaks=
                        100
                        , 
                        main=
                        ""
                        , 
                        col=
                        "grey80"
                        ,
     
                        xlab=expression
                        (Log[
                        10
                        ]
                            ^~^
                        
                        "average count"
                        ))

                        abline
                        (
                        v=log10
                        (
                        1
                        ), 
                        col=
                        "blue"
                        , 
                        lwd=
                        2
                        , 
                        lty=
                        2
                        )
                    


**Figure 6.**
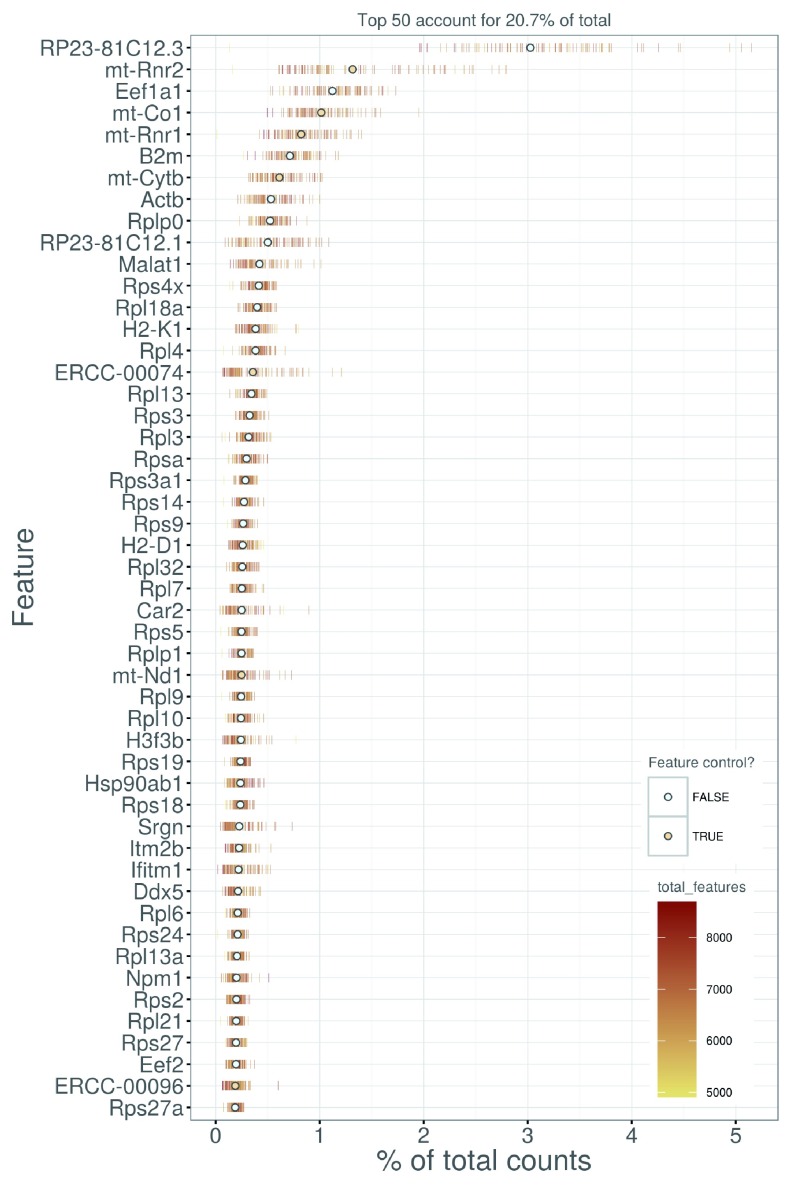
Percentage of total counts assigned to the top 50 most highly-abundant features in the HSC dataset. For each feature, each bar represents the percentage assigned to that feature for a single cell, while the circle represents the average across all cells. Bars are coloured by the total number of expressed features in each cell, while circles are coloured according to whether the feature is labelled as a control feature.



We also look at the identities of the most highly expressed genes ( Figure 6). This should generally be dominated by constitutively expressed transcripts, such as those for ribosomal or mitochondrial proteins. The presence of other classes of features may be cause for concern if they are not consistent with expected biology. For example, a top set containing many spike-in transcripts suggests that too much spike-in RNA was added during library preparation, while the absence of ribosomal proteins and/or the presence of their pseudogenes are indicative of suboptimal alignment.

                        plotQC
                        (sce, 
                        type = 
                        "highest-expression"
                        , 
                        n=
                        50
                        ) + fontsize
                    


An alternative approach to gene filtering is to select genes that have non-zero counts in at least *n* cells. This provides some more protection against genes with outlier expression patterns, i.e., strong expression in only one or two cells. Such outliers are typically uninteresting as they can arise from amplification artifacts that are not replicable across cells. (The exception is for studies involving rare cells where the outliers may be biologically relevant.) An example of this filtering approach is shown below for *n* set to 10, though smaller values may be necessary to retain genes expressed in rare cell types.



                        numcells <- 
                        nexprs
                        (sce, 
                        byrow=
                        TRUE
                        )

                        alt.keep <- numcells >= 
                        10

                        sum
                        (alt.keep)
                    




                        ## [1] 11988
                    


**Figure 7.**
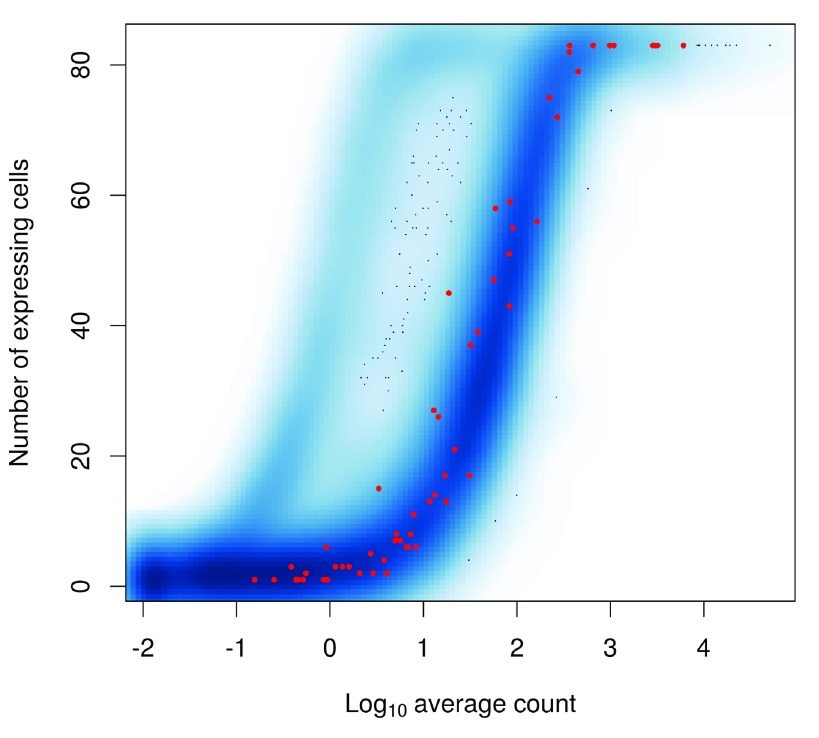
Number of expressing cells against the log-mean expression for each gene in the HSC dataset. Spike-in transcripts are highlighted in red.



The relationship between the number of expressing cells and the mean is shown in Figure 7. The two statistics tend to be well-correlated so filtering on either should give roughly similar results.

                        smoothScatter
                        (
                        log10
                        (ave.counts), numcells, 
                        xlab=expression
                        (Log[
                        10
                        ]
                            ^~^
                        
                        "average count"
                        ),
    
                         ylab=
                        "Number of expressing cells"
                        )

                        is.ercc <- 
                        isSpike
                        (sce, 
                        type=
                        "ERCC"
                        )

                        points
                        (
                        log10
                        (ave.counts[is.ercc]), numcells[is.ercc], 
                        col=
                        "red"
                        , 
                        pch=
                        16
                        , 
                        cex=
                        0.5
                        )
                    


In general, we prefer the mean-based filter as it tends to be less aggressive. A gene will be retained as long as it has sufficient expression in any subset of cells. Genes expressed in fewer cells require higher levels of expression in those cells to be retained, but this is not undesirable as it avoids selecting uninformative genes (with low expression in few cells) that contribute little to downstream analyses, e.g., HVG detection or clustering. In contrast, the “at least *n*” filter depends heavily on the choice of *n*. With *n* = 10, a gene expressed in a subset of 9 cells would be filtered out, regardless of the level of expression in those cells. This may result in the failure to detect rare subpopulations that are present at frequencies below *n*. While the mean-based filter will retain more outlier-driven genes, this can be handled by choosing methods that are robust to outliers in the downstream analyses.

Thus, we apply the mean-based filter to the data by subsetting the SCESet object as shown below. This removes all rows corresponding to endogenous genes or spike-in transcripts with abundances below the specified threshold.



                        sce <- sce[keep,]
                    


### Normalization of cell-specific biases

***Using the deconvolution method to deal with zero counts.*** Read counts are subject to differences in capture efficiency and sequencing depth between cells ( Stegle *et al.*, 2015). Normalization is required to eliminate these cell-specific biases prior to downstream quantitative analyses. This is often done by assuming that most genes are not differentially expressed (DE) between cells. Any systematic difference in count size across the non-DE majority of genes between two cells is assumed to represent bias and is removed by scaling. More specifically, “size factors” are calculated that represent the extent to which counts should be scaled in each library.

Size factors can be computed with several different approaches, e.g., using the estimateSizeFactorsFromMatrix function in the *DESeq2* package ( Anders & Huber, 2010; Love *et al.*, 2014), or with the calcNormFactors function ( Robinson & Oshlack, 2010) in the *edgeR* package. However, single-cell data can be problematic for these bulk data-based methods due to the dominance of low and zero counts. To overcome this, we pool counts from many cells to increase the count size for accurate size factor estimation ( Lun *et al.*, 2016). Pool-based size factors are then “deconvolved” into cell-based factors for cell-specific normalization.



                        sce <- 
                        computeSumFactors
                        (sce, 
                        sizes=c
                        (
                        20
                        , 
                        40
                        , 
                        60
                        , 
                        80
                        ))

                        summary
                        (
                        sizeFactors
                        (sce))
                    




                        ##     Min.  1st Qu.   Median     Mean  3rd Qu.     Max.

                        ##   0.4161   0.8055   0.9434   1.0000   1.1890   1.8410
                    


**Figure 8.**
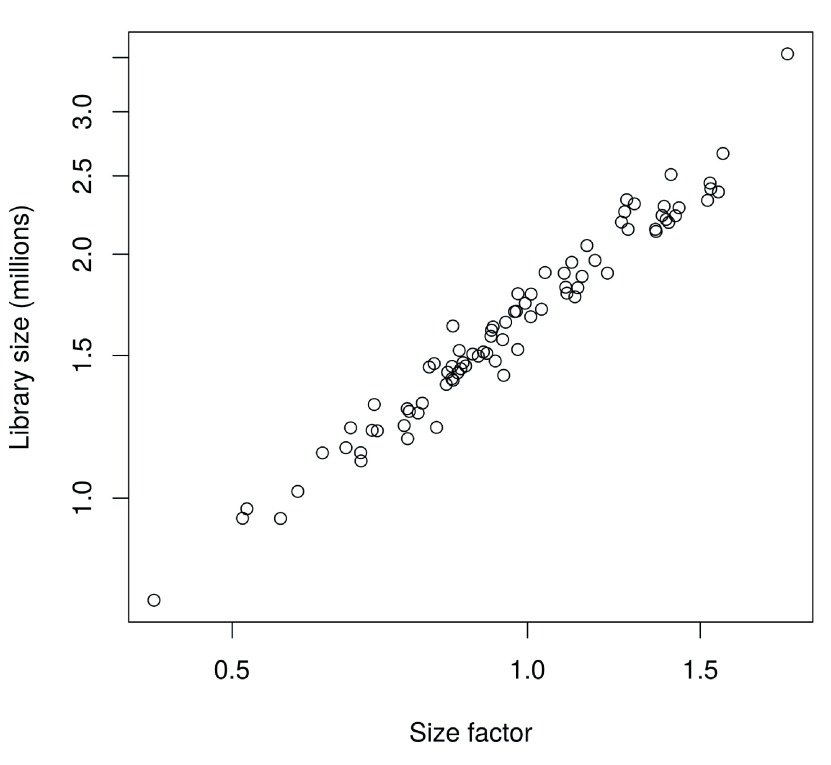
Size factors from deconvolution, plotted against library sizes for all cells in the HSC dataset. Axes are shown on a log-scale.



In this case, the size factors are tightly correlated with the library sizes for all cells ( Figure 8). This suggests that the systematic differences between cells are primarily driven by differences in capture efficiency or sequencing depth. Any DE between cells would yield a non-linear trend between the total count and size factor, and/or increased scatter around the trend. This does not occur here as strong DE is unlikely to exist within a homogeneous population of cells.

                        plot
                        (
                        sizeFactors
                        (sce), sce$total_counts/
                        1e6
                        , 
                        log=
                        "xy"
                        ,
     
                        ylab=
                        "Library size (millions)"
                        , 
                        xlab=
                        "Size factor"
                        )
                    


***Computing separate size factors for spike-in transcripts.*** Size factors computed from the counts for endogenous genes are usually not appropriate for normalizing the counts for spike-in transcripts. Consider an experiment without library quantification, i.e., the amount of cDNA from each library is *not* equalized prior to pooling and multiplexed sequencing. Here, cells containing more RNA have greater counts for endogenous genes and thus larger size factors to scale down those counts. However, the same amount of spike-in RNA is added to each cell during library preparation. This means that the counts for spike-in transcripts are not subject to the effects of RNA content. Attempting to normalize the spike-in counts with the gene-based size factors will lead to over-normalization and incorrect quantification of expression. Similar reasoning applies in cases where library quantification is performed. For a constant total amount of cDNA, any increases in endogenous RNA content will suppress the coverage of spike-in transcripts. As a result, the bias in the spike-in counts will be opposite to that captured by the gene-based size factor.

To ensure normalization is performed correctly, we compute a separate set of size factors for the spike-in set. For each cell, the spike-in-specific size factor is defined as the total count across all transcripts in the spike-in set. This assumes that none of the spike-in transcripts are differentially expressed, which is reasonable given that the same amount and composition of spike-in RNA should have been added to each cell. (See below for a more detailed discussion on spike-in normalization.) These size factors are stored in a separate field of the SCESet object by setting general.use=FALSE in computeSpikeFactors. This ensures that they will only be used with the spike-in transcripts but not the endogenous genes.



                        sce <- 
                        computeSpikeFactors
                        (sce, 
                        type=
                        "ERCC"
                        , 
                        general.use=
                        FALSE
                        )
                    


***Applying the size factors to normalize gene expression***. The count data are used to compute normalized log-expression values for use in downstream analyses. Each value is defined as the log-ratio of each count to the size factor for the corresponding cell, after adding a prior count of 1 to avoid undefined values at zero counts. Division by the size factor ensures that any cell-specific biases are removed. If spike-in-specific size factors are present in sce, they will be automatically applied to normalize the spike-in transcripts separately from the endogenous genes.



                        sce <- 
                        normalize
                        (sce)
                    


The log-transformation provides some measure of variance stabilization ( Law *et al.*, 2014), so that high-abundance genes with large variances do not dominate downstream analyses. The computed values are stored as an exprs matrix in addition to the other assay elements.

### Checking for important technical factors

We check whether there are technical factors that contribute substantially to the heterogeneity of gene expression. If so, the factor may need to be regressed out to ensure that it does not inflate the variances or introduce spurious correlations. For this dataset, the simple experimental design means that there are no plate or batch effects to examine. Instead, we use the (log-transformed) total count for the spike-in transcripts as a proxy for the relative bias in each sample. This bias is purely technical in origin, given that the same amount of spike-in RNA should have been added to each cell. Thus, any association of gene expression with this factor is not biologically interesting and should be removed.

**Figure 9.**
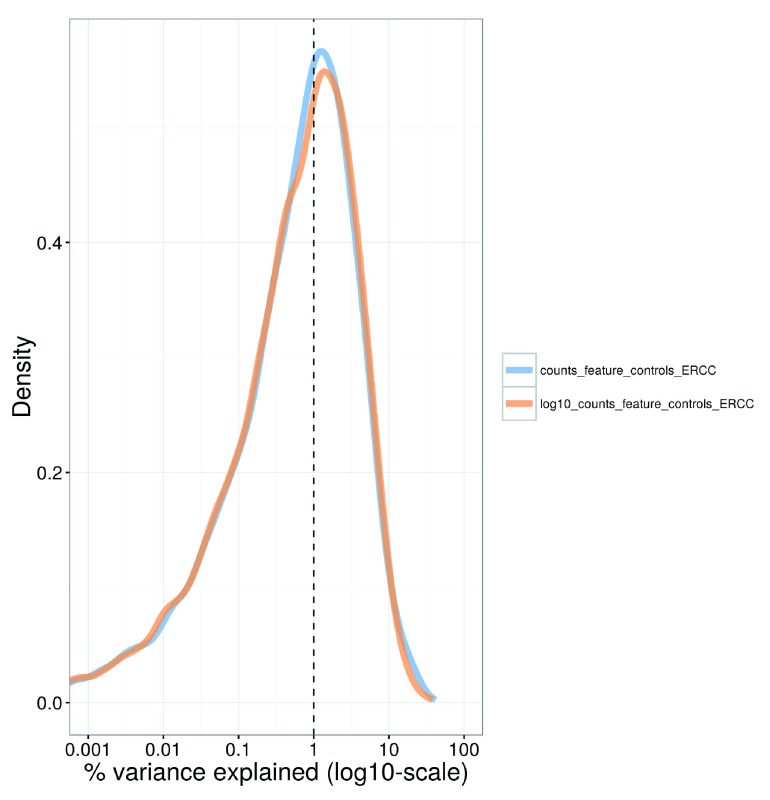
Density plot of the percentage of variance explained by the (log-transformed) total spike-in counts across all genes in the HSC dataset. For each gene, the percentage of the variance of the normalized log-expression values across cells that is explained by each factor is calculated. Each curve corresponds to one factor and represents the distribution of percentages across all genes.



For each gene, we calculate the percentage of the variance of the expression values that is explained by the spike-in totals ( Figure 9). The percentages are generally small (1–3%), indicating that the expression profiles of most genes are not strongly associated with this factor. This result is consistent with successful removal of cell-specific biases by scaling normalization. Thus, the spike-in total does not need to be explicitly modelled in our downstream analyses.

                        plotExplanatoryVariables
                        (sce, 
                        variables=c
                        (
                        "counts_feature_controls_ERCC"
                        ,
     
                        "log10_counts_feature_controls_ERCC"
                        )) + fontsize
                    


Note that the use of the spike-in total as an accurate proxy for the relative technical bias assumes that no library quantification is performed. Otherwise, the coverage of the spike-in transcripts would be dependent on the total amount of endogenous RNA in each cell. (Specifically, if the same amount of cDNA is used for sequencing per cell, any increase in the amount of endogenous RNA will suppress the coverage of the spike-in transcripts.) This means that the spike-in totals could be confounded with genuine biological effects associated with changes in RNA content.

### Identifying HVGs from the normalized log-expression

We identify HVGs to focus on the genes that are driving heterogeneity across the population of cells. This requires estimation of the variance in expression for each gene, followed by decomposition of the variance into biological and technical components. HVGs are then identified as those genes with the largest biological components. This avoids prioritizing genes that are highly variable due to technical factors such as sampling noise during RNA capture and library preparation.

Ideally, the technical component would be estimated by fitting a mean-variance trend to the spike-in transcripts using the trendVar function. Recall that the same set of spike-ins was added in the same quantity to each cell. This means that the spike-in transcripts should exhibit no biological variability, i.e., any variance in their counts should be technical in origin. Given the mean abundance of a gene, the fitted value of the trend can be used as an estimate of the technical component for that gene. The biological component of the variance can then be calculated by subtracting the technical component from the total variance of each gene with the decomposeVar function.

In practice, this strategy is compromised by the small number of spike-in transcripts, the uneven distribution of their abundances and (for low numbers of cells) the imprecision of their variance estimates. This makes it difficult to accurately fit a complex mean-dependent trend to the spike-in variances. An alternative approach is to fit the trend to the variance estimates of the endogenous genes, using the use.spikes=FALSE setting as shown below. This assumes that the majority of genes are not variably expressed, such that the technical component dominates the total variance for those genes. The fitted value of the trend is then used as an estimate of the technical component. Obviously, this is the only approach that can be used if no spike-ins were added in the experiment.



                        var.fit <- 
                        trendVar
                        (sce, 
                        trend=
                        "loess"
                        , 
                        use.spikes=
                        FALSE
                        , 
                        span=
                        0.2
                        )

                        var.out <- 
                        decomposeVar
                        (sce, var.fit)
                    


**Figure 10.**
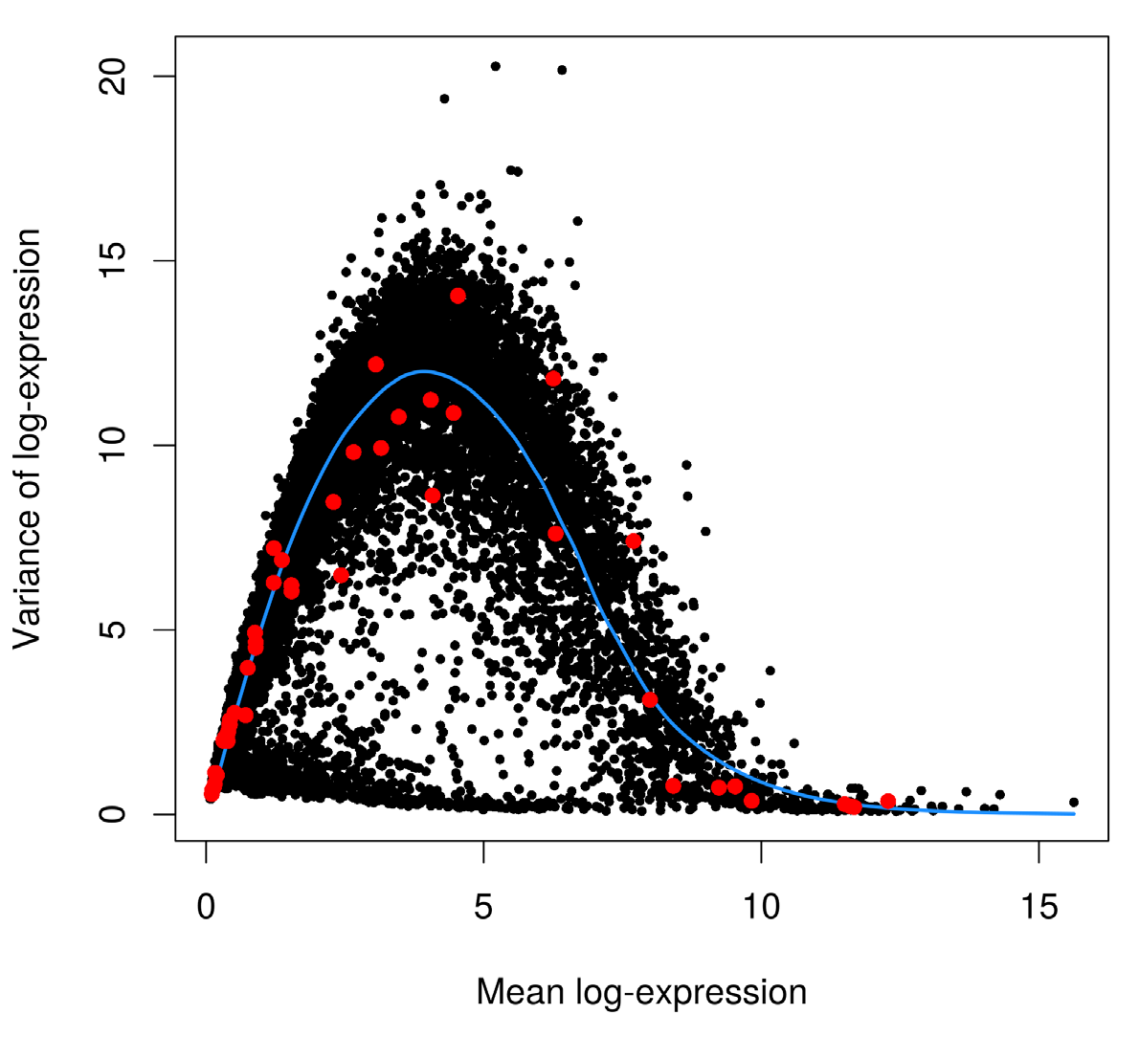
Variance of normalized log-expression values for each gene in the HSC dataset, plotted against the mean log-expression. The blue line represents the mean-dependent trend fitted to the variances of the endogenous genes. Variance estimates for spike-in transcripts are highlighted in red.



We assess the suitability of the trend fitted to the endogenous variances by examining whether it is consistent with the spike-in variances ( Figure 10). The trend passes through or close to most of the spike-in variances, indicating that our assumption (that most genes have low levels of biological variability) is valid. This strategy exploits the large number of endogenous genes to obtain a stable trend, with the spike-in transcripts used as diagnostic features rather than in the trend fitting itself. However, if our assumption did *not* hold, we would instead fit the trend directly to the spike-in variances with the default use.spikes=TRUE. This sacrifices stability to reduce systematic errors in the estimate of the biological component for each gene. (In such cases, tinkering with the trend fitting parameters may yield a more stable curve – see ?trendVar for more details.)

                        plot
                        (var.out$mean, var.out$total, 
                        pch=
                        16
                        , 
                        cex=
                        0.6
                        , 
                        xlab=
                        "Mean log-expression"
                        ,
     
                        ylab=
                        "Variance of log-expression"
                        )

                        o <- 
                        order
                        (var.out$mean)

                        lines
                        (var.out$mean[o], var.out$tech[o], 
                        col=
                        "dodgerblue"
                        , 
                        lwd=
                        2)

                        cur.spike <- 
                        isSpike
                        (sce)

                        points
                        (var.out$mean[cur.spike], var.out$total[cur.spike], 
                        col=
                        "red"
                        , 
                        pch=
                        16
                        )
                    


HVGs are defined as genes with biological components that are significantly greater than zero at a false discovery rate (FDR) of 5%. These genes are interesting as they drive differences in the expression profiles between cells, and should be prioritized for further investigation. In addition, we only consider a gene to be a HVG if it has a biological component greater than or equal to 0.5. For transformed expression values on the log _2_ scale, this means that the average difference in true expression between any two cells will be at least 2-fold. (This reasoning assumes that the true log-expression values are Normally distributed with variance of 0.5. The root-mean-square of the difference between two values is treated as the average log _2_-fold change between cells and is equal to unity.) We rank the results by the biological component to focus on genes with larger biological variability.



                        hvg.out <- var.out[
                        which
                        (var.out$FDR <= 
                        0.05 
                        & var.out$bio >= 
                        0.5
                        ),]
hvg.out <- hvg.out[
                        order
                        (hvg.out$bio, 
                        decreasing=
                        TRUE
                        ),]

                        nrow
                        (hvg.out)
                    




                        ## [1] 193
                    




                        write.table
                        (
                        file=
                        "hsc_hvg.tsv"
                        , 
                        hvg.out, 
                        sep=
                        "\t"
                        , 
                        quote=
                        FALSE
                        , 
                        col.names=
                        NA
                        )

                        head
                        (hvg.out)
                    




                        ##              mean     total       bio      tech       p.value           FDR
## Fos      6.412282 20.167804 12.287746  7.880058  3.609804e-13  2.283693e-10
## Rgs1     5.214003 20.271925  9.430165 10.841761  3.065697e-06  5.019808e-04
## Dusp1    6.693026 16.074489  9.044983  7.029506  3.066936e-10  1.156266e-07
## H2-Aa    4.294426 19.390442  7.496497 11.893945  2.736909e-04  2.333494e-02
## Ppp1r15a 6.545438 14.964370  7.460786  7.503584  2.308822e-07  4.943721e-05
## Ctla2a   8.654347  9.471605  7.368337  2.103268  4.574748e-38  9.095906e-35
                    


**Figure 11.**
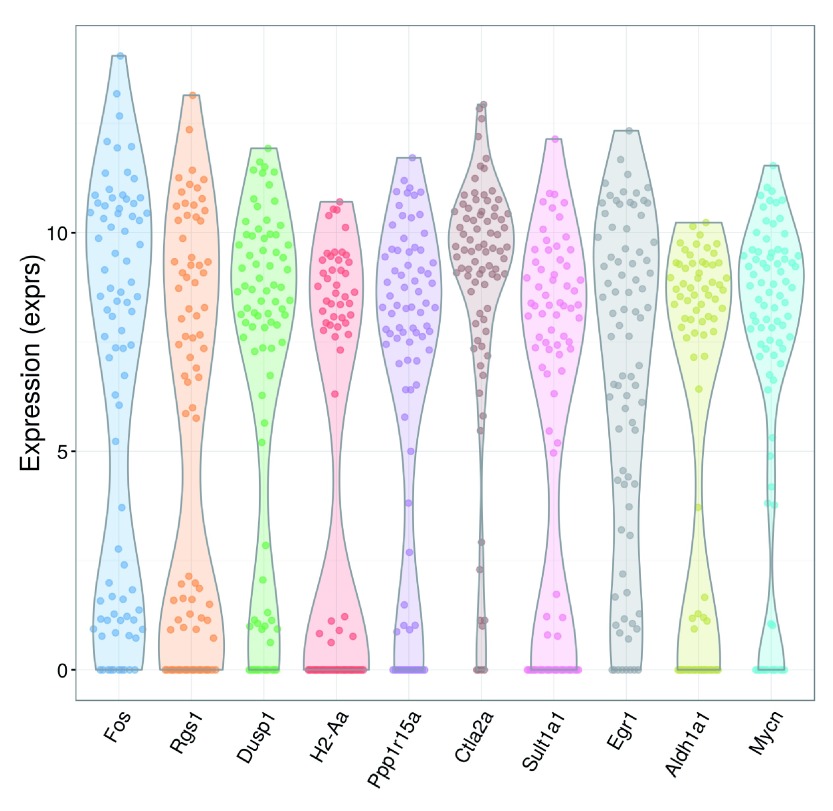
Violin plots of normalized log-expression values for the top 10 HVGs in the HSC dataset. Each point represents the log-expression value in a single cell.



We recommend checking the distribution of expression values for the top HVGs to ensure that the variance estimate is not being dominated by one or two outlier cells ( Figure 11).

                        plotExpression
                        (sce, 
                        rownames
                        (hvg.out)[
                        1
                        :
                        10
                        ]) + fontsize
                    


There are many other strategies for defining HVGs, e.g., by using the coefficient of variation ( Brennecke *et al.*, 2013; Kim *et al.*, 2015; Kolodziejczyk *et al.*, 2015), with the dispersion parameter in the negative binomial distribution ( McCarthy *et al.*, 2012), or as a proportion of total variability ( Vallejos *et al.*, 2015). Some of these methods are available in *scran* – for example, see DM or technicalCV2 for calculations based on the coefficient of variation. Here, we use the variance of the log-expression values because the log-transformation protects against genes with strong expression in only one or two cells. This ensures that the set of top HVGs is not dominated by genes with (mostly uninteresting) outlier expression patterns.

### Identifying correlated gene pairs with Spearman’s rho

Another useful procedure is to identify the HVGs that are highly correlated with one another. This distinguishes between HVGs caused by random noise and those involved in driving systematic differences between subpopulations. Correlations between genes are quantified by computing Spearman's rho, which accommodates non-linear relationships in the expression values. Gene pairs with significantly large positive or negative values of rho are identified using the correlatePairs function. We only apply this function to the set of HVGs, because these genes have large biological components and are more likely to exhibit strong correlations driven by biology. In contrast, calculating correlations for all possible gene pairs would require too much computational time and increase the severity of the multiple testing correction. It may also prioritize uninteresting genes that have strong correlations but low variance, e.g., tightly co-regulated house-keeping genes.



                        set.seed
                        (
                        100
                        )

                        var.cor <- 
                        correlatePairs
                        (sce, 
                        subset.row=rownames
                        (hvg.out))

                        write.table
                        (
                        file=
                        "hsc_cor.tsv"
                        , var.cor
                        , 
                        sep=
                        "\t"
                        , 
                        quote=
                        FALSE
                        , 
                        row.names=
                        FALSE
                        )

                        head
                        (var.cor)
                    




                        ##      gene1   gene2       rho      p.value         FDR
## 1   mt-Nd2 mt-Rnr1 0.6037110 1.999998e-06 0.005293709
## 2     Egr1     Jun 0.5218295 1.999998e-06 0.005293709
## 3    Pdia6   Hspa5 0.5119852 1.999998e-06 0.005293709
## 4      Fos    Egr1 0.5035263 1.999998e-06 0.005293709
## 5 Ppp1r15a   Zfp36 0.4975862 1.999998e-06 0.005293709
## 6   Hnrpdl  mt-Nd2 0.4963688 1.999998e-06 0.005293709
                    


The significance of each correlation is determined using a permutation test. For each pair of genes, the null hypothesis is that the expression profiles of two genes are independent. Shuffling the profiles and recalculating the correlation yields a null distribution that is used to obtain a *p*-value for each observed correlation value ( Phipson & Smyth, 2010). Correction for multiple testing across many gene pairs is performed by controlling the FDR at 5%. Correlated gene pairs can be directly used for experimental validation with orthogonal techniques (e.g., fluorescence-activated cell sorting, immunohistochemistry or RNA fluorescence *in situ* hybridization) to verify that these expression patterns are genuinely present across the cell population.



                        sig.cor <- var.cor$FDR <= 
                        0.05

                        summary
                        (sig.cor)
                    




                        ##    Mode FALSE TRUE NA’s
## logical 18485   43    0
                    


Larger sets of correlated genes are assembled by treating genes as nodes in a graph and each pair of genes with significantly large correlations as an edge. In particular, an undirected graph is constructed using methods in the *RBGL* package. Highly connected subgraphs are then identified and defined as gene sets. This provides a convenient summary of the pairwise correlations between genes.



                        library
                        (RBGL)
g <- 
                        ftM2graphNEL
                        (
                        cbind
                        (var.cor$gene1, var.cor$gene2)[sig.cor,],
     
                        W=
                        NULL
                        , 
                        V=
                        NULL
                        , 
                        edgemode=
                        "undirected"
                        )
cl <- 
                        highlyConnSG
                        (g)$clusters
cl <- cl[
                        order
                        (
                        lengths
                        (cl), 
                        decreasing=
                        TRUE
                        )
                        ]

                        head
                        (cl)
                    




                        ## [[1]]
## [1] "Egr1"  "Fos"   "Zfp36" "Ier2"
##
## [[2]]
## [1] "mt-Nd2"  "Sh3bgrl" "mt-Rnr1"
##
## [[3]]
## [1] "Hspd1"   "Pik3ip1" "Srm"
##
## [[4]]
## [1] "Sqstm1" "Phgdh"  "Cct3"
##
## [[5]]
## [1] "Morf4l2" "Impdh2"  "Ncl"
##
## [[6]]
## [1] "Hsd17b12" "Srsf7"
                    


Significant correlations provide evidence for substructure in the dataset, i.e., subpopulations of cells with systematic differences in their expression profiles. The number of significantly correlated HVG pairs represents the strength of the substructure. If many pairs were significant, this would indicate that the subpopulations were clearly defined and distinct from one another. For this particular dataset, a relatively low number of HVGs exhibit significant correlations. This suggests that any substructure in the data will be modest, which is expected given that rigorous selection was performed to obtain a homogeneous population of HSCs ( Wilson *et al.*, 2015).

### Using correlated HVGs for further data exploration

**Figure 12.**
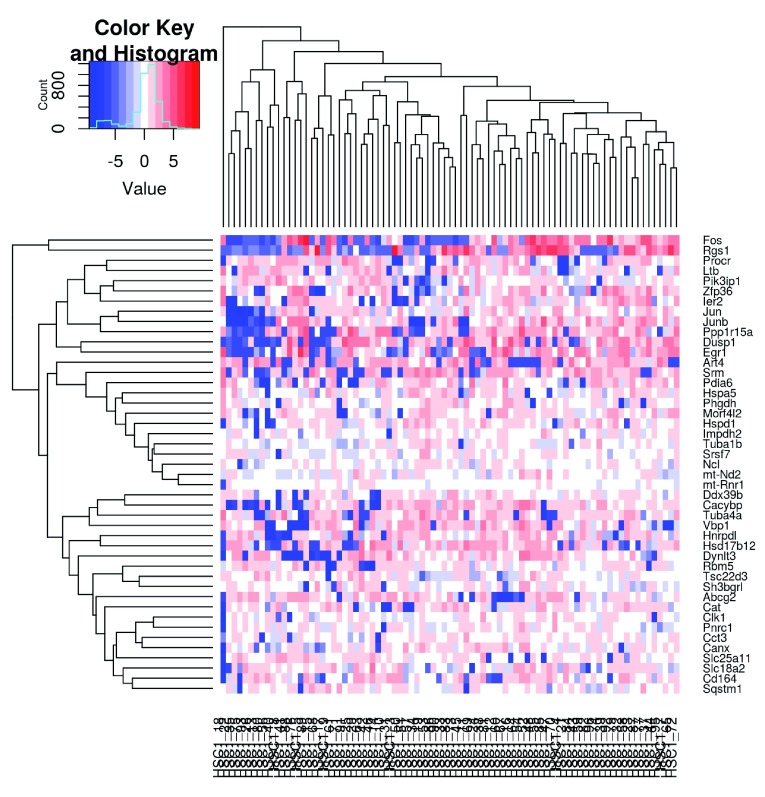
Heatmap of mean-centred normalized log-expression values for correlated HVGs in the HSC dataset. Dendrograms are formed by hierarchical clustering on the Euclidean distances between genes (row) or cells (column).



We visualize the expression profiles of the correlated HVGs with a heatmap ( Figure 12). All expression values are mean-centred for each gene to highlight the relative differences in expression between cells. If any subpopulations were present, they would manifest as rectangular “blocks” in the heatmap, corresponding to sets of genes that are systematically up- or down-regulated in specific groups of cells. This is not observed in Figure 12, consistent with the lack of strong substructure. There may be a subpopulation of *Fos* and *Jun*-negative cells, but it is poorly defined given the small numbers of cells and genes involved.

                        chosen <- 
                        unique
                        (
                        c
                        (var.cor$gene1[sig.cor], var.cor$gene2[sig.cor]))
norm.exprs <- 
                        exprs
                        (sce)[chosen,,drop=
                        FALSE
                        ]
heat.vals <- norm.exprs - 
                        rowMeans
                        (norm.exprs)

                        library
                        (gplots)
heat.out <- 
                        heatmap.2
                        (heat.vals, 
                        col=
                        bluered, 
                        symbreak=
                        TRUE
                        , 
                        trace=
                        ’none’
                        , 
                        cexRow=
                        0.6
                        )
                    


**Figure 13.**
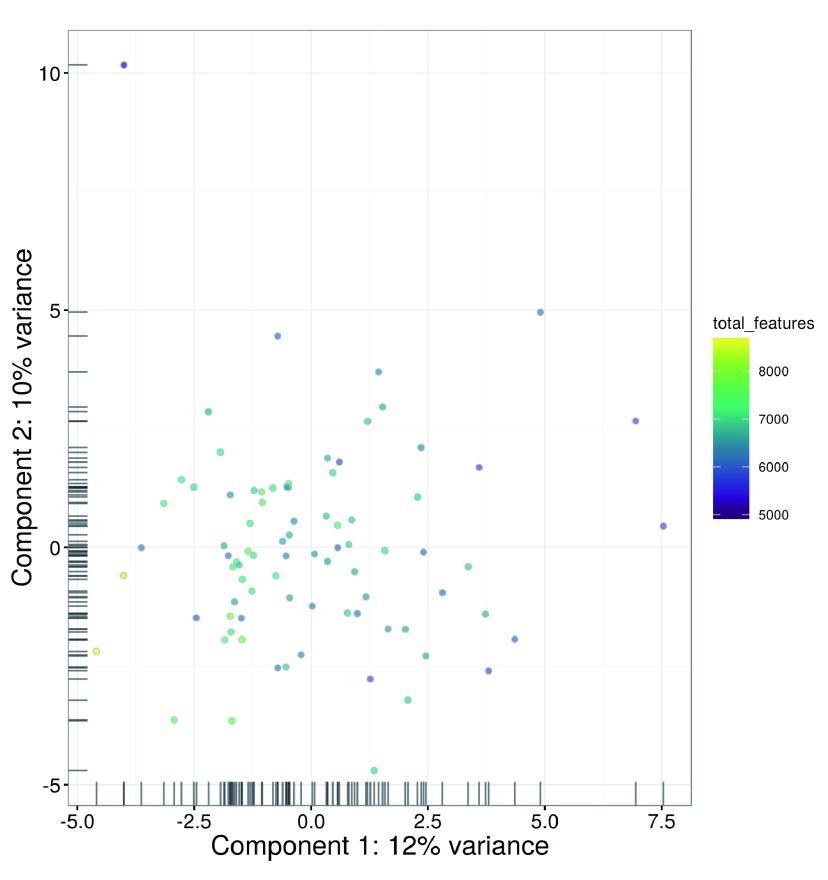
PCA plot constructed from normalized log-expression values of correlated HVGs, where each point represents a cell in the HSC dataset. First and second components are shown, along with the percentage of variance explained. Bars represent the coordinates of the cells on each axis. Each cell is coloured according to its total number of expressed features.



We also apply dimensionality reduction techniques to visualize the relationships between cells. This is done by constructing a PCA plot from the normalized log-expression values of the correlated HVGs ( Figure 13). Cells with similar expression profiles should be located close together in the plot, while dissimilar cells should be far apart. We only use the correlated HVGs in plotPCA because any substructure should be most pronounced in the expression profiles of these genes. Even so, no clear separation of cells into distinct subpopulations is observed.

                        plotPCA
                        (sce, 
                        exprs_values=
                        "exprs"
                        , 
                        colour_by=
                        "total_features"
                        ,
     
                        feature_set=
                        chosen) + fontsize
                    


On a related note, we only show the first two components that contribute most to the variance in Figure 13. Additional components can be visualized by increasing the ncomponents argument in plotPCA to construct pairwise plots. The percentage of variance explained by each component can also be obtained by running plotPCA with return_SCESet=TRUE, and then calling reducedDimension on the returned object. This information may be useful for selecting high-variance components (possibly corresponding to interesting underlying factors) for further examination.

**Figure 14.**
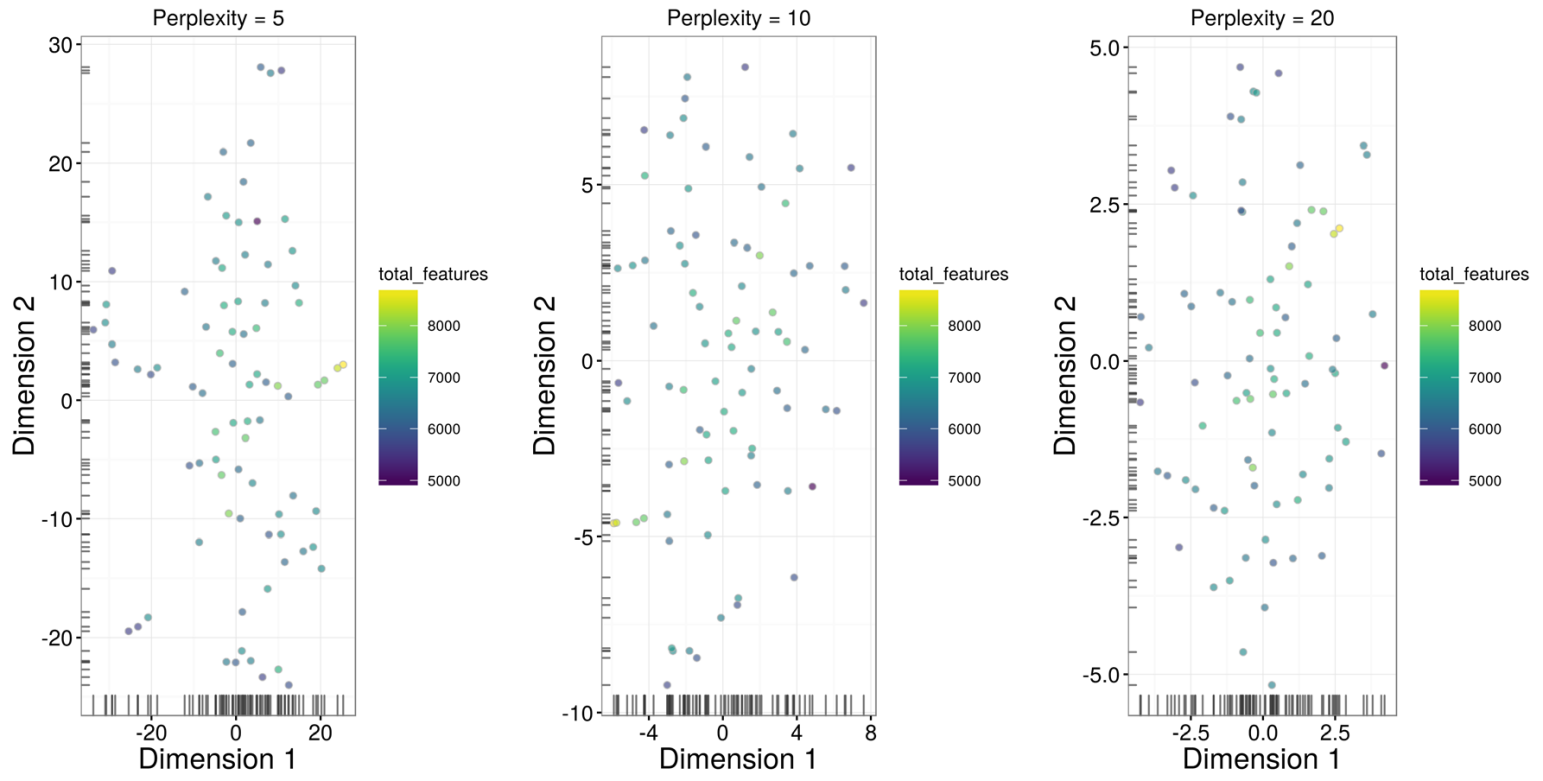
*t*-SNE plots constructed from normalized log-expression values of correlated HVGs, using a range of perplexity values. In each plot, each point represents a cell in the HSC dataset. Bars represent the coordinates of the cells on each axis. Each cell is coloured according to its total number of expressed features.



Another widely used approach is the *t*-stochastic neighbour embedding ( *t*-SNE) method ( Van der Maaten & Hinton, 2008). *t*-SNE tends to work better than PCA for separating cells in more diverse populations. This is because the former can directly capture non-linear relationships in high-dimensional space, whereas the latter must represent them (suboptimally) as linear components. However, this improvement comes at the cost of more computational effort and complexity. In particular, *t*-SNE is a stochastic method, so users should run the algorithm several times to ensure that the results are representative, and then set a seed to ensure that the chosen results are reproducible. It is also advisable to test different settings of the “perplexity” parameter as this will affect the distribution of points in the low-dimensional space. This is demonstrated below in Figure 14, though no consistent substructure is observed in all plots.

                        set.seed
                        (
                        100
                        )

                        out5 <- 
                        plotTSNE
                        (sce, 
                        exprs_values=
                        "exprs"
                        , 
                        perplexity=
                        5
                        , 
                        colour_by=
                        "total_features"
                        ,
     
                        feature_set=
                        chosen) + fontsize + 
                        ggtitle
                        (
                        "Perplexity = 5"
                        )

                        out10 <- 
                        plotTSNE
                        (sce, 
                        exprs_values=
                        "exprs"
                        , 
                        perplexity=
                        10
                        , 
                        colour_by=
                        "total_features"
                        ,
     
                        feature_set=
                        chosen) + fontsize + 
                        ggtitle
                        (
                        "Perplexity = 10"
                        )

                        out20 <- 
                        plotTSNE
                        (sce, 
                        exprs_values=
                        "exprs"
                        , 
                        perplexity=
                        20
                        , 
                        colour_by=
                        "total_features"
                        ,
     
                        feature_set=
                        chosen) + fontsize + 
                        ggtitle
                        (
                        "Perplexity = 20"
                        )

                        multiplot
                        (out5, out10, out20, 
                        cols=
                        3
                        )
                    


There are many other dimensionality reduction techniques that we do not consider here but could also be used, e.g., multidimensional scaling, diffusion maps. These have their own advantages and disadvantages – for example, diffusion maps (see plotDiffusionMap) place cells along a continuous trajectory and are suited for visualizing graduated processes like differentiation ( Angerer *et al.*, 2016). For each visualization method, additional cell-specific information can be incorporated into the colour, size or shape of each point. Here, cells are coloured by the total number of expressed features to demonstrate that this metric does not drive any systematic differences across the population. The selectorPlot function from *scran* can also be used to interactively select groups of cells in two-dimensional space. This facilitates data exploration as visually identified subpopulations can be directly selected for further examination.

Finally, putative subpopulations can be computationally defined by cutting the dendrogram in heat.out$colDendrogram with cutree to form clusters. We do not attempt this here as the substructure is too weak for reliable clustering. In fact, users should generally treat clustering results with some caution. If the differences between cells are subtle, the assignment of cells into clusters may not be robust. Moreover, different algorithms can yield substantially different clusters by focusing on different aspects of the data. Experimental validation of the clusters is critical to ensure that the putative subpopulations actually exist.

### Additional comments

Once the basic analysis is completed, it is often useful to save the SCESet object to file with the saveRDS function. The object can then be easily restored into new R sessions using the readRDS function. This allows further work to be conducted without having to repeat all of the processing steps described above.



                        saveRDS
                        (
                        file=
                        "hsc_data.rds"
                        , sce)
                    


A variety of methods are available to perform more complex analyses on the processed expression data. For example, cells can be ordered in pseudotime (e.g., for progress along a differentiation pathway) with *monocle* ( Trapnell *et al.*, 2014) or *TSCAN* ( Ji & Ji, 2016); cell-state hierarchies can be characterized with the *sincell* package ( Julia *et al.*, 2015); and oscillatory behaviour can be identified using *Oscope* ( Leng *et al.*, 2015). HVGs can be used in gene set enrichment analyses to identify biological pathways and processes with heterogeneous activity, using packages designed for bulk data like *topGO* or with dedicated single-cell methods like *scde* ( Fan *et al.*, 2016). Full descriptions of these analyses are outside the scope of this workflow, so interested users are advised to consult the relevant documentation.

## Analysis of cell types in the brain

### Overview

We proceed to a more heterogeneous dataset from a study of cell types in the mouse brain ( Zeisel *et al.*, 2015). This contains approximately 3000 cells of varying types such as oligodendrocytes, microglia and neurons. Individual cells were isolated using the Fluidigm C1 microfluidics system and library preparation was performed on each cell using a UMI-based protocol. After sequencing, expression was quantified by counting the number of UMIs mapped to each gene. Count data for all endogenous genes, mitochondrial genes and spike-in transcripts were obtained from http://linnarssonlab.org/cortex.

### Count loading

The count data are distributed across several files, so some work is necessary to consolidate them into a single matrix. We define a simple utility function for loading data in from each file. (We stress that this function is only relevant to the current dataset, and should not be used for other datasets. This kind of effort is generally not required if all of the counts are in a single file and separated from the metadata.)



                        readFormat <- function(infile) {
     
                        # First column is empty.
     
                        metadata <- 
                        read.delim
                        (infile, 
                        stringsAsFactors=
                        FALSE
                        , 
                        header=
                        FALSE
                        , 
                        nrow=
                        10
                        )[,-
                        1
                        ]
     
                        rownames
                        (metadata) <- metadata[,
                        1
                        ]
     
                        metadata <- metadata[,-
                        1
                        ]
     
                        metadata <- 
                        as.data.frame
                        (
                        t
                        (metadata))
     
                        # First column after row names is some useless filler.
     
                        counts <- 
                        read.delim
                        (infile, 
                        stringsAsFactors=
                        FALSE
                        , 
                        header=
                        FALSE
                        , 
                        row.names=
                        1
                        , 
                        skip=
                        11
                        )[,-
                        1
                        ]
     
                        counts <- 
                        as.matrix
                        (counts)
     
                        return
                        (
                        list
                        (
                        metadata=
                        metadata, 
                        counts=
                        counts))
}
                    


Using this function, we read in the counts for the endogenous genes, ERCC spike-ins and mitochondrial genes.



                        endo.data <- 
                        readFormat
                        (
                        "expression_mRNA_17-Aug-2014.txt"
                        )

                        spike.data <- 
                        readFormat
                        (
                        "expression_spikes_17-Aug-2014.txt"
                        )

                        mito.data <- 
                        readFormat
                        (
                        "expression_mito_17-Aug-2014.txt"
                        )
                    


We also need to rearrange the columns for the mitochondrial data, as the order is not consistent with the other files.



                        m <- 
                        match
                        (endo.data$metadata$cell_id, mito.data$metadata$cell_id)
mito.data$metadata <- mito.data$metadata[m,]
mito.data$counts <- mito.data$counts[,m]
                    


The counts are then combined into a single matrix for constructing a SCESet object. For convenience, metadata for all cells are stored in the same object for later access.



                        all.counts <- 
                        rbind
                        (endo.data$counts, mito.data$counts, spike.data$counts)
metadata <- 
                        AnnotatedDataFrame
                        (endo.data$metadata)
sce <- 
                        newSCESet
                        (
                        countData=
                        all.counts, 
                        phenoData=
                        metadata)

                        dim
                        (sce)
                    




                        ## Features Samples
##    20063    3005
                    


We also add annotation identifying rows that correspond to each class of features.



                        nrows <- 
                        c
                        (
                        nrow
                        (endo.data$counts), 
                        nrow
                        (mito.data$counts), 
                        nrow
                        (spike.data$counts))
is.spike <- 
                        rep
                        (
                        c
                        (
                        FALSE
                        , 
                        FALSE
                        , 
                        TRUE
                        ), nrows)
is.mito <- 
                        rep
                        (
                        c
                        (
                        FALSE
                        , 
                        TRUE
                        , 
                        FALSE
                        ), nrows)
                    


### Quality control on the cells

The original authors of the study have already removed low-quality cells prior to data publication. Nonetheless, we compute some quality control metrics to check whether the remaining cells are satisfactory.



                        sce <- 
                        calculateQCMetrics
                        (sce, 
                        feature_controls=list
                        (
                        Spike=
                        is.spike, 
                        Mt=
                        is.mito))

                        isSpike
                        (sce) <- 
                        "Spike"
                    


**Figure 15.**
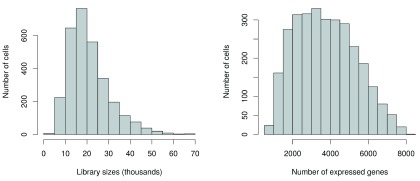
Histograms of library sizes (left) and number of expressed genes (right) for all cells in the brain dataset.



We examine the distribution of library sizes and numbers of expressed genes across cells ( Figure 15).

                        par
                        (
                        mfrow=c
                        (
                        1,
                        2
                        ))

                        hist
                        (sce$total_counts/
                        1e3
                        , 
                        xlab=
                        "Library sizes (thousands)"
                        , 
                        main=
                        ""
                        ,
     
                        breaks=
                        20
                        , 
                        col=
                        "grey80"
                        , 
                        ylab=
                        "Number of cells"
                        )

                        hist
                        (sce$total_features, 
                        xlab=
                        "Number of expressed genes"
                        , 
                        main=
                        ""
                        ,
     
                        breaks=
                        20
                        , 
                        col=
                        "grey80"
                        , 
                        ylab=
                        "Number of cells"
                        )
                    


**Figure 16.**
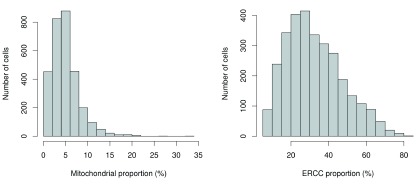
Histogram of the proportion of UMIs assigned to mitochondrial genes (left) or spike-in transcripts (right) across all cells in the brain dataset.

We also examine the distribution of the proportions of UMIs assigned to mitochondrial genes or spike-in transcripts ( Figure 16). The spike-in proportions here are more variable than in the HSC dataset. This may reflect a greater variability in the total amount of endogenous RNA per cell when many cell types are present.



                        par
                        (
                        mfrow=c
                        (
                        1
                        ,
                        2
                        ))

                        hist
                        (sce$pct_counts_feature_controls_Mt, 
                        xlab=
                        "Mitochondrial proportion (%)"
                        ,
     
                        ylab=
                        "Number of cells"
                        , 
                        breaks=
                        20
                        , 
                        main=
                        ""
                        , 
                        col=
                        "grey80"
                        )

                        hist
                        (sce$pct_counts_feature_controls_Spike, 
                        xlab=
                        "ERCC proportion (%)"
                        ,
     
                        ylab=
                        "Number of cells"
                        , 
                        breaks=
                        20
                        , 
                        main=
                        ""
                        , 
                        col=
                        "grey80"
                        )
                    


We remove small outliers in Figure 15 and large outliers in Figure 16, using a MAD-based threshold as previously described.



                        libsize.drop <- 
                        isOutlier
                        (sce$total_counts, 
                        nmads=
                        3
                        , 
                        type=
                        "lower"
                        , 
                        log=
                        TRUE
                        )
feature.drop <- 
                        isOutlier
                        (sce$total_features, 
                        nmads=
                        3
                        , 
                        type=
                        "lower"
                        , 
                        log=
                        TRUE
                        )
mito.drop <- 
                        isOutlier
                        (sce$pct_counts_feature_controls_Mt, 
                        nmads=
                        3
                        , 
                        type=
                        "higher"
                        )
spike.drop <- 
                        isOutlier
                        (sce$pct_counts_feature_controls_Spike, 
                        nmads=
                        3
                        , 
                        type=
                        "higher"
                        )
                    


Removal of low-quality cells is then performed by combining the filters for all of the metrics. The vast majority of cells are retained, which suggests that the original quality control procedures were generally adequate.



                        sce <- sce[,!(libsize.drop | feature.drop | spike.drop | mito.drop)]

                        data.frame
                        (
                        ByLibSize=sum
                        (libsize.drop), 
                        ByFeature=sum
                        (feature.drop),
    
                         ByMito=sum
                        (mito.drop), 
                        BySpike=sum
                        (spike.drop), 
                        Remaining=ncol
                        (sce))
                    




                        ## 	    ByLibSize ByFeature ByMito BySpike Remaining
##  Samples         8         3     87       8      2902
                    


### Cell cycle classification

**Figure 17.**
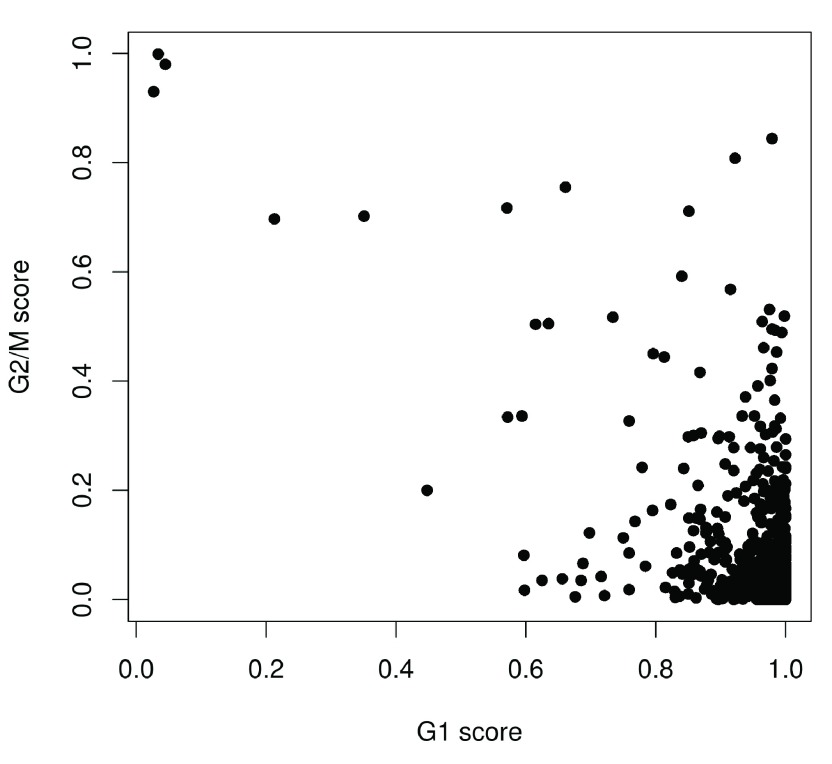
Cell cycle phase scores from applying the pair-based classifier on the brain dataset, where each point represents a cell.



Application of cyclone to the brain dataset suggests that most of the cells are in G1 phase ( Figure 17). However, the intepretation of this result requires some caution due to the differences between the test and training datasets. The classifier was trained on C1 SMARTer data ( Scialdone *et al.*, 2015) and accounts for the biases in that protocol. The brain dataset uses UMI counts, which has an entirely different set of biases, e.g., 3’-end coverage only, no length bias, no amplification noise. These new biases (and the absence of expected biases) may interfere with accurate classification of some cells.

                        anno <- 
                        select
                        (org.Mm.eg.db, 
                        keys=rownames
                        (sce), 
                        keytype=
                        "SYMBOL"
                        , 
                        column=
                        "ENSEMBL"
                        )
ensembl <- anno$ENSEMBL[
                        match
                        (
                        rownames
                        (sce), anno$SYMBOL)]
assignments <- 
                        cyclone
                        (sce, mm.pairs, 
                        gene.names=
                        ensembl)

                        plot
                        (assignments$score$G1, assignments$score$G2M, 
                        xlab=
                        "G1 score"
                        , 
                        ylab=
                        "G2/M score"
                        , 
                        pch=
                        16
                        )
                    


An additional complication is that many neuronal cell types are expected to lie in the G0 resting phase, which is distinct from the other phases of the cell cycle ( Coller *et al.*, 2006). Application of cyclone to these cells may be suboptimal if each cell must be assigned into one of the G1, S or G2/Mphases. To avoid problems from misclassification, we will not perform any processing of this dataset by cell cycle phase. This is unlikely to be problematic for this analysis, as the cell cycle effect will be relatively subtle compared to the obvious differences between cell types in a diverse population. Thus, the former is unlikely to distort the conclusions regarding the latter.

### Removing uninteresting genes

Low-abundance genes are removed by applying a simple mean-based filter. We use a lower threshold for UMI counts compared to that used for read counts. This is because the number of transcript molecules will always be lower than the number of reads generated from such molecules. While some information and power will be lost due to the decrease in the size of the counts, this is mitigated by a concomitant reduction in the variability of the counts. Specifically, the use of UMIs eliminates technical noise due to amplification biases ( Islam *et al.*, 2014).



                        ave.counts <- 
                        rowMeans
                        (
                        counts
                        (sce))

                        keep <- 
                        rowMeans
                        (
                        counts
                        (sce)) >= 
                        0.2
                    


**Figure 18.**
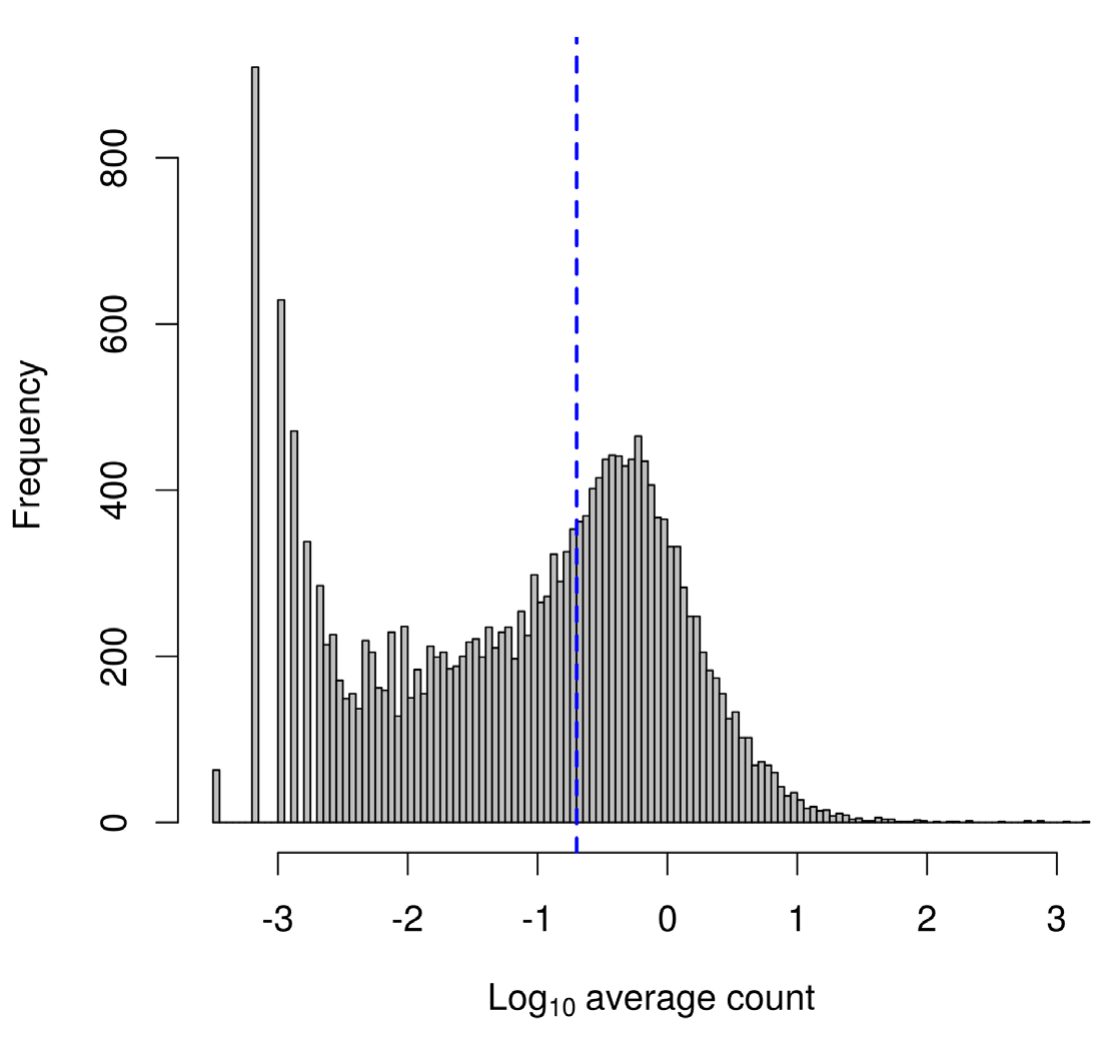
Histogram of log-average counts for all genes in the brain dataset. The filter threshold is represented by the blue line.



Figure 18 suggests that our choice of threshold is appropriate. The filter removes the bulk of lowly expressed genes while preserving the peak of moderately expressed genes.

                        hist
                        (
                        log10
                        (ave.counts), 
                        breaks=
                        100
                        , 
                        main=
                        ""
                        , 
                        col=
                        "grey"
                        ,
     
                        xlab=expression
                        (Log[
                        10
                        ]
                            ^~^
                        
                        "average count"
                        ))

                        abline
                        (
                        v=log10
                        (
                        0.2)
                        , 
                        col=
                        "blue"
                        , 
                        lwd=
                        2
                        , 
                        lty=
                        2
                        )
                    


The mean-based filter is applied to the dataset by subsetting sce as previously described. Despite the reduced threshold, the number of retained genes is lower than that in the HSC dataset, simply because the library sizes are much smaller with UMI counts.



                        sce <- sce[keep,]

                        nrow
                        (sce)
                    




                        ## Features
##     8939
                    


Some datasets also contain strong heterogeneity in mitochondrial RNA content, possibly due to differences in mitochondrial copy number or activity between cell types. This heterogeneity will cause mitochondrial genes to dominate the top set of results, e.g., for identification of correlated HVGs. However, these genes are largely uninteresting given that most studies focus on nuclear regulation. As such, we filter them out prior to further analysis. Other candidates for removal include pseudogenes or ribosome-associated genes, which might not be relevant for characterising cell types but can still interfere with the interpretation of the results.



                        sce <- sce[!
                        fData
                        (sce)$is_feature_control_Mt,]
                    


### Normalization of cell-specific biases

Normalization of cell-specific biases is performed using the deconvolution method in the computeSumFactors function. Here, we cluster similar cells together and normalize the cells in each cluster using the deconvolution method. This improves normalization accuracy by reducing the number of DE genes between cells in the same cluster. Scaling is then performed to ensure that size factors of cells in different clusters are comparable.



                        clusters <- 
                        quickCluster
                        (sce)
sce <- 
                        computeSumFactors
                        (sce, 
                        cluster
                        =clusters)
                    


**Figure 19.**
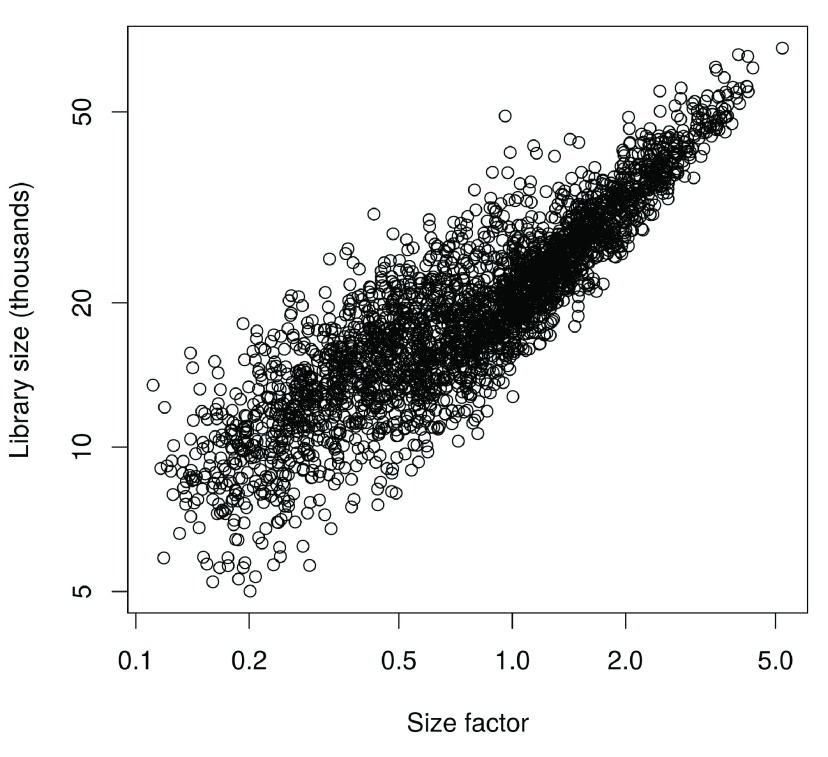
Size factors from deconvolution, plotted against library sizes for all cells in the brain dataset. Axes are shown on a log-scale.



Compared to the HSC analysis, more scatter is observed around the trend between the total count and size factor for each cell ( Figure 19). This is consistent with an increased amount of DE between cells of different types, which compromises the accuracy of library size normalization ( Robinson & Oshlack, 2010). In contrast, the size factors are estimated based on median ratios and are more robust to the presence of DE between cells.

                        plot
                        (
                        sizeFactors
                        (sce), sce$total_counts/
                        1e3
                        , 
                        log=
                        "xy"
                        ,
    
                        ylab=
                        "Library size (thousands)"
                        , 
                        xlab=
                        "Size factor"
                        )
                    


We also compute size factors specific to the spike-in set, as previously described.



                        sce <- 
                        computeSpikeFactors
                        (sce, 
                        type=
                        "Spike"
                        , 
                        general.use=
                        FALSE
                        )
                    


Finally, normalized log-expression values are computed for each endogenous gene or spike-in transcript using the appropriate size factors.



                        sce <- 
                        normalize
                        (sce)
                    


### Checking for important technical factors

**Figure 20.**
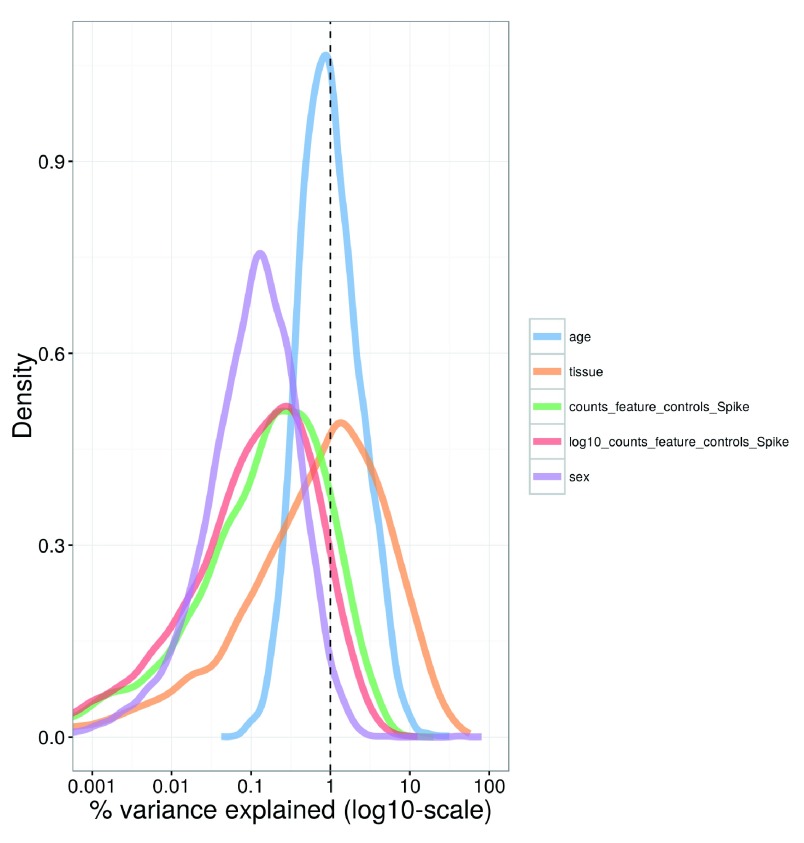
Density plot of the percentage of variance explained by each factor across all genes in the brain dataset. For each gene, the percentage of the variance of the normalized log-expression values that is explained by the (log-transformed) total spike-in counts, the sex or age of the mouse, or the tissue of origin is calculated. Each curve corresponds to one factor and represents the distribution of percentages across all genes.



Larger experiments contain more technical factors that need to be investigated. In this dataset, factors include the sex of the animal from which the cells were extracted, the age of the animal, the tissue of origin for each cell, and the total spike-in count in each cell. Figure 20 shows that the tissue of origin explains a substantial proportion of the variance for a subset of genes. This is probably because each tissue contains a different composition of cell types, leading to systematic differences in gene expression between tissues. The other factors explain only a small proportion of the variance for most genes and do not need to be incorporated into our downstream analyses.

                        plotExplanatoryVariables
                        (sce, 
                        variables=c
                        (
                        "counts_feature_controls_Spike"
                        ,
    
                        "log10_counts_feature_controls_Spike"
                        , 
                        "sex"
                        , 
                        "tissue"
                        , 
                        "age"
                        )) + fontsize
                    


Nonetheless, we demonstrate how to account for uninteresting technical factors by using sex as an example. We set up a design matrix with the sex of the animal as the explanatory factor for each cell. This ensures that any sex-specific changes in expression will be modelled in our downstream analyses. We do not block on the tissue of origin, despite the fact that it explains more of the variance than sex in Figure 20. This is because the tissue factor is likely to be associated with genuine differences between cell types, so including it in the model might regress out interesting biological effects.



                        design <- 
                        model.matrix
                        (
                            ^~^sce$sex)
                    


Other relevant factors include the chip or plate on which the cells were processed and the batch in which the libraries were sequenced. Blocking on these factors may be necessary to account for batch effects that are often observed in scRNA-seq data ( Hicks *et al.*, 2015; Tung *et al.*, 2016).

### Identifying correlated HVGs

We identify HVGs that may be involved in driving population heterogeneity. This is done by fitting a trend to the technical variances for the spike-in transcripts. We then compute the biological component of the variance for each endogenous gene by subtracting the fitted value of the trend from the total variance.



                        var.fit <- 
                        trendVar
                        (sce, 
                        trend=
                        "loess"
                        , 
                        design=
                        design, 
                        span=
                        0.4
                        )
var.out <- 
                        decomposeVar
                        (sce, var.fit)
                    


**Figure 21.**
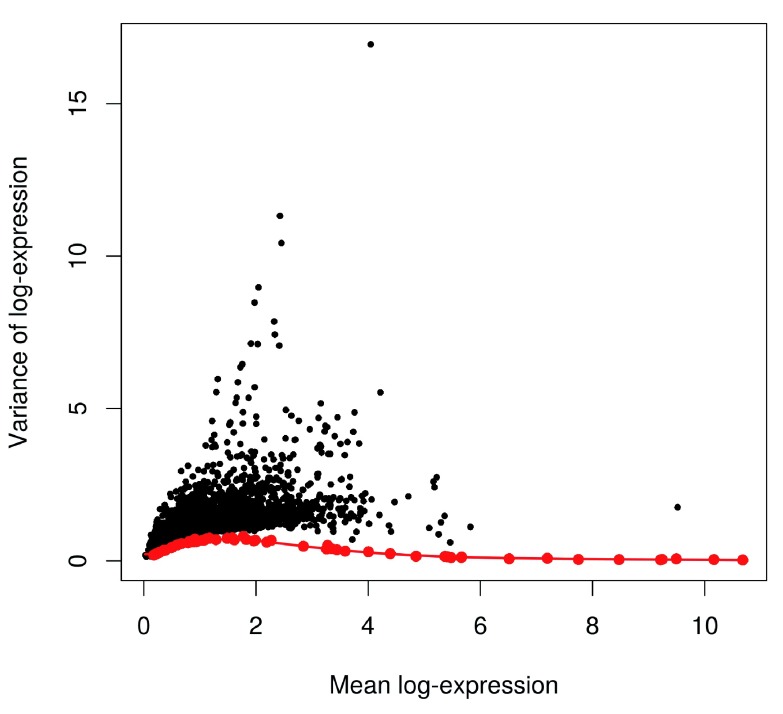
Variance of normalized log-expression values for each gene in the brain dataset, plotted against the mean log-expression. The red line represents the mean-dependent trend in the technical variance of the spike-in transcripts (also highlighted as red points).



Figure 21 suggests that the trend is fitted accurately to the technical variances. Errors in fitting are negligible due to the precision of the variance estimates in a large dataset containing thousands of cells. The technical and total variances are also much smaller than those in the HSC dataset. This is due to the use of UMIs which reduces the noise caused by variable PCR amplification. Furthermore, the spike-in trend is consistently lower than the variances of the endogenous genes. This reflects the heterogeneity in gene expression across cells of different types. It also means the previous strategy of fitting a trend to the endogenous variances would not be appropriate here (or necessary, given the quality of the spike-in trend).

                        plot
                        (var.out$mean, var.out$total, 
                        pch=
                        16
                        , 
                        cex=
                        0.6
                        , 
                        xlab=
                        "Mean log-expression"
                        ,
    
                        ylab=
                        "Variance of log-expression"
                        )

                        points
                        (var.fit$mean, var.fit$var, 
                        col=
                        "red"
                        , 
                        pch=
                        16
                        )
o <- 
                        order
                        (var.out$mean)

                        lines
                        (var.out$mean[o], var.out$tech[o], 
                        col=
                        "red"
                        , 
                        lwd=
                        2
                        )
                    


HVGs are identified as genes with large positive biological components. These are saved to file for future reference. Note that some of the p-values are reported as zero due to numerical imprecision.



                        hvg.out <- var.out[
                        which
                        (var.out$FDR <= 
                        0.05 
                        & var.out$bio >= 
                        0.5
                        ),]
hvg.out <- hvg.out[
                        order
                        (hvg.out$bio, 
                        decreasing=
                        TRUE
                        ),]

                        nrow
                        (hvg.out)
                    




                        ## [1] 1755
                    




                        write.table
                        (
                        file=
                        "brain_hvg.tsv"
                        , hvg.out, 
                        sep=
                        "\t"
                        , 
                        quote=
                        FALSE
                        , 
                        col.names=
                        NA
                        )

                        head
                        (hvg.out)
                    




                        ## 	    mean     total       bio      tech p.value FDR
## Plp1 4.045420 16.949056 16.681804 0.2672513       0   0
## Trf  2.427692 11.317924 10.745370 0.5725539       0   0
## Mal  2.454213 10.427362  9.860428 0.5669333       0   0
## Apod 2.044163  8.973862  8.319578 0.6542837       0   0
## Mog  1.974681  8.472565  7.803619 0.6689461       0   0
## Mbp  2.324417  7.853273  7.259729 0.5935431       0   0
                    


**Figure 22.**
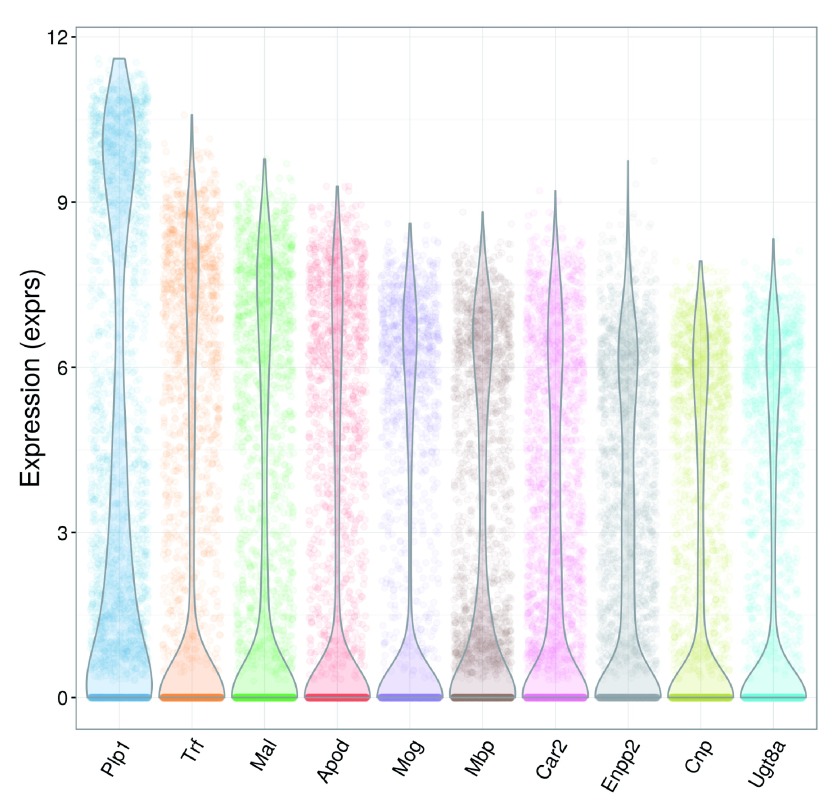
Violin plots of normalized log-expression values for the top 10 HVGs in the brain dataset. For each gene, each point represents the log-expression value for an individual cell.



Again, we check the distribution of expression values for the top 10 HVGs to ensure that they are not being driven by outliers ( Figure 22). Some tweaking of the plotExpression parameters is necessary to visualize a large number of cells.

                        plotExpression
                        (sce, 
                        rownames
                        (hvg.out)[
                        1
                        :
                        10
                        ], 
                        alpha=
                        0.05
                        , 
                        jitter=
                        "jitter"
                        ) + fontsize
                    


To identify genes involved in defining subpopulations, the set of HVGs is tested for significant pairwise correlations. Given the size of the set, we only use the top 500 HVGs to reduce computational work. Here, the number of significantly correlated pairs is much higher than in the HSC dataset, indicating that strong substructure is present. These results are also saved to file for use in designing validation experiments.



                        set.seed
                        (
                        100
                        )
var.cor <- 
                        correlatePairs
                        (sce, 
                        design=
                        design, 
                        subset.row=rownames
                        (hvg.out)[
                        1
                        :
                        500
                        ])

                        write.table
                        (
                        file=
                        "brain_cor.tsv"
                        , var.cor, 
                        sep=
                        "\t"
                        , 
                        quote=
                        FALSE
                        , 
                        row.names=
                        FALSE
                        )

                        head
                        (var.cor)
                    




                        ##    gene1  gene2 	 rho      p.value          FDR
## 1   Meg3 Snhg11 0.8542706 1.999998e-06 2.611414e-06
## 2 Snap25  Stmn2 0.8023813 1.999998e-06 2.611414e-06
## 3 Ppp3ca  Prkcb 0.7977351 1.999998e-06 2.611414e-06
## 4 Atp1b1   Rtn1 0.7959162 1.999998e-06 2.611414e-06
## 5  Stmn3  Stmn2 0.7958141 1.999998e-06 2.611414e-06
## 6 Snap25  Ndrg4 0.7938286 1.999998e-06 2.611414e-06
                    





                        sig.cor <- var.cor$FDR <= 
                        0.05

                        sum
                        (sig.cor)
                    





                        ## [1] 111798
                    


### Further data exploration with the correlated HVGs

We first remove the sex effect using the removeBatchEffect function from the *limma* package ( Ritchie *et al.*, 2015). This ensures that any sex-specific differences will not dominate the visualization of the expression profiles. In this manner, we maintain consistency with the use of design in the previous steps. (However, if an analysis method can accept a design matrix, blocking on nuisance factors in the design matrix is preferable to manipulating the expression values with removeBatchEffect. This is because the latter does not account for the loss of residual degrees of freedom, nor the uncertainty of estimation of the blocking factor terms.) We store these sex-corrected expression values in the norm_exprs field of the SCESet object for later use.




                        library
                        (limma)

                        adj.exprs <- 
                        exprs
                        (sce)

                        adj.exprs <- 
                        removeBatchEffect
                        (adj.exprs, 
                        batch=
                        sce$sex)

                        norm_exprs
                        (sce) <- adj.exprs
                    


**Figure 23.**
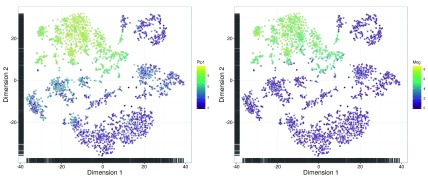
*t*-SNE plots constructed from the normalized and corrected log-expression values of correlated HVGs for cells in the brain dataset. Each point represents a cell and is coloured according to its expression of the top HVG (left) or *Mog* (right).



We perform dimensionality reduction on the correlated HVGs to check if there is any substructure. Cells separate into clear clusters in the *t*-SNE plot ( Figure 23), corresponding to distinct subpopulations. This is consistent with the presence of multiple cell types in the diverse brain population.

                        chosen <- 
                        unique
                        (
                        c
                        (var.cor$gene1[sig.cor], var.cor$gene2[sig.cor]))

                        top.hvg <- 
                        rownames
                        (hvg.out)[
                        1
                        ]

                        tsne1 <- 
                        plotTSNE
                        (sce, 
                        exprs_values=
                        "norm_exprs"
                        , 
                        colour_by=
                        top.hvg,
    
                        perplexity=
                        10
                        , 
                        rand_seed=
                        100
                        , 
                        feature_set=
                        chosen) + fontsize

                        tsne2 <- 
                        plotTSNE
                        (sce, 
                        exprs_values=
                        "norm_exprs"
                        , 
                        colour_by=
                        "Mog"
                        ,
    
                        perplexity=
                        10
                        , 
                        rand_seed=
                        100
                        , 
                        feature_set=
                        chosen) + fontsize

                        multiplot
                        (tsne1, tsne2, 
                        cols=
                        2
                        )
                    


**Figure 24.**
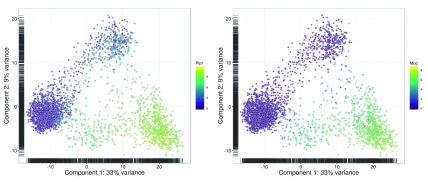
PCA plots constructed from the normalized and corrected log-expression values of correlated HVGs for cells in the brain dataset. Each point represents a cell and is coloured according to its expression of the top HVG (left) or *Mog* (right).



The PCA plot is less effective at separating cells into many different clusters ( Figure 24). This is because the first two principal components are driven by strong differences between specific subpopulations, which reduces the resolution of more subtle differences between some of the other subpopulations. Nonetheless, some substructure is still visible.

                        pca1 <- 
                        plotPCA
                        (sce, 
                        exprs_values=
                        "norm_exprs"
                        , 
                        colour_by=
                        top.hvg) + fontsize

                        pca2 <- 
                        plotPCA
                        (sce, 
                        exprs_values=
                        "norm_exprs"
                        , 
                        colour_by=
                        "Mog"
                        ) + fontsize

                        multiplot
                        (pca1, pca2, 
                        cols=
                        2
                        )
                    


For both methods, we colour each cell based on the expression of a particular gene. This is a useful strategy for visualizing changes in expression across the lower-dimensional space. It can also be used to characterise each cluster if the selected genes are known markers for particular cell types. For example, *Mog* can be used to identify clusters corresponding to oligodendrocytes.

### Clustering cells into putative subpopulations

The normalized and sex-adjusted log-expression values for correlated HVGs are used to cluster cells into putative subpopulations. Specifically, we perform hierarchical clustering on the Euclidean distances between cells, using Ward’s criterion to minimize the total variance within each cluster. This yields a dendrogram that groups together cells with similar expression patterns across the chosen genes. An alternative approach is to cluster on a matrix of distances derived from correlations (e.g., as in quickCluster). This is more robust to noise and normalization errors, but is also less sensitive to subtle changes in the expression profiles.



                        chosen.exprs <- 
                        norm_exprs
                        (sce)[chosen,]

                        my.dist <- 
                        dist
                        (
                        t
                        (chosen.exprs))

                        my.tree <- 
                        hclust
                        (my.dist, 
                        method=
                        "ward.D2"
                        )
                    


Clusters are explicitly defined by applying a dynamic tree cut ( Langfelder *et al.*, 2008) to the dendrogram. This exploits the shape of the branches in the dendrogram to refine the cluster definitions, and is more appropriate than cutree for complex dendrograms. Greater control of the empirical clusters can be obtained by manually specifying cutHeight in cutreeDynamic.



                        library
                        (dynamicTreeCut)

                        my.clusters <- 
                        unname
                        (
                        cutreeDynamic
                        (my.tree, 
                        distM=as.matrix
                        (my.dist), 
                        verbose=
                        0
                        ))
                    


**Figure 25.**
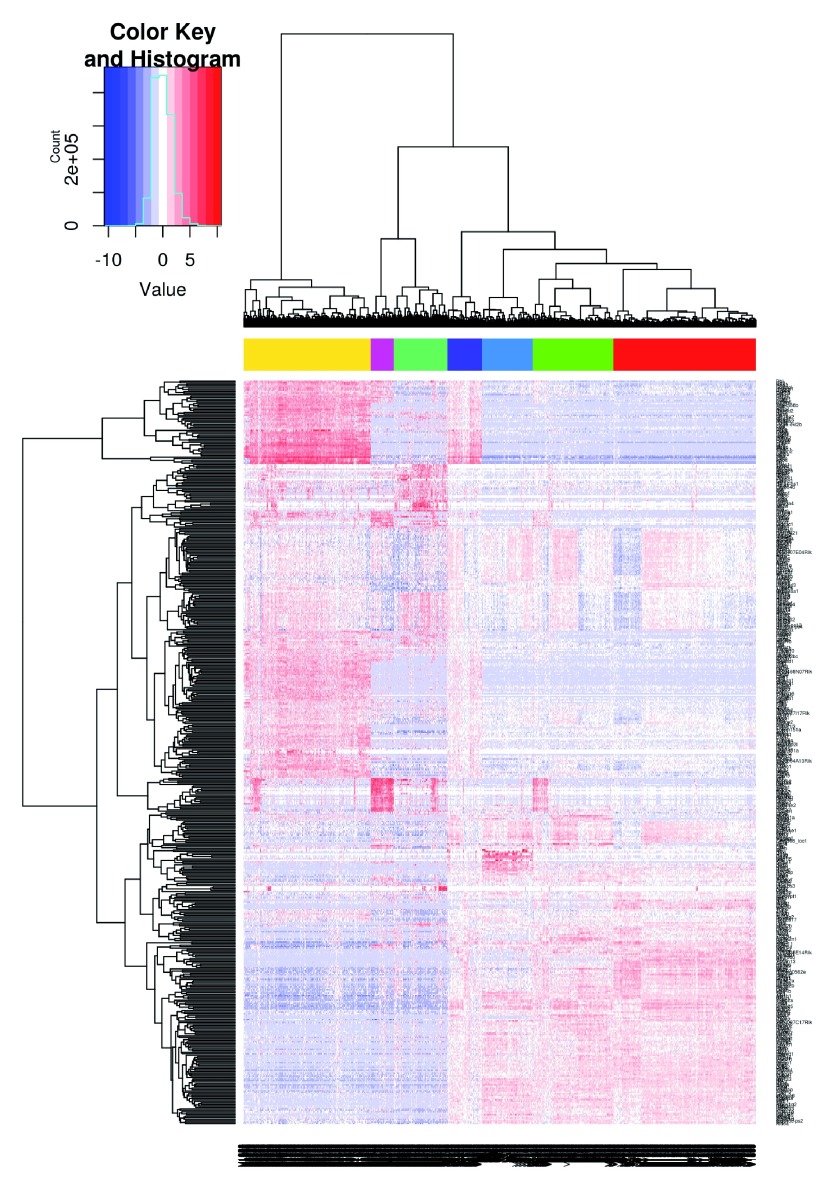
Heatmap of mean-centred normalized and corrected log-expression values for correlated HVGs in the brain dataset. Dendrograms are formed by hierarchical clustering on the Euclidean distances between genes (row) or cells (column). Column colours represent the cluster to which each cell is assigned after a dynamic tree cut.



Figure 25 contains a clear block-like pattern, representing systematic differences between clusters of cells with distinct expression profiles. This is consistent with the presence of well-defined subpopulations that were previously observed in the dimensionality reduction plots.

                        heat.vals <- chosen.exprs - 
                        rowMeans
                        (chosen.exprs)

                        clust.col <- 
                        rainbow
                        (
                        max
                        (my.clusters))

                        heatmap.2
                        (heat.vals, 
                        col=
                        bluered, 
                        symbreak=
                        TRUE
                        , 
                        trace=
                        ’none’
                        , 
                        cexRow=
                        0.3
                        ,
    
                        ColSideColors=
                        clust.col[my.clusters], 
                        Colv=as.dendrogram
                        (my.tree))
                    


This heatmap can be stored at a greater resolution for detailed inspection later.



                        pdf
                        (
                        "brain_heat.pdf"
                        , 
                        width=
                        20
                        , 
                        height=
                        40
                        )

                        heatmap.2
                        (heat.vals, 
                        col=
                        bluered, 
                        symbreak=
                        TRUE
                        , 
                        trace=
                        ’none’
                        , 
                        cexRow=
                        0.3
                        ,
    
                        ColSideColors=
                        clust.col[my.clusters], 
                        Colv=as.dendrogram
                        (my.tree))

                        dev.off
                        ()
                    


### Detecting marker genes between subpopulations

Once putative subpopulations are identified, we can identify marker genes for specific subpopulations of interest. This is done by identifying genes that are consistently DE in one subpopulation compared to the others. DE testing can be performed using a number of packages, but for this workflow, we will use the *edgeR* package ( Robinson *et al.*, 2010). First, we set up a design matrix specifying which cells belong to each cluster. Each cluster* coefficient represents the average log-expression of all cells in the corresponding cluster. We also block on uninteresting factors such as sex.



                        cluster <- 
                        factor
                        (my.clusters)

                        de.design <- 
                        model.matrix
                        (
                            ^~^
                        
                        0 
                        + cluster + sce$sex)

                        head
                        (
                        colnames
                        (de.design))
                    




                        ## [1] "cluster1" "cluster2" "cluster3" "cluster4" "cluster5" "cluster6"
                    


We set up a DGEList object for entry into the *edgeR* analysis. This new object contains all relevant information from the original SCESet object, including the counts and (library size-adjusted) size factors.



                        library
                        (edgeR)

                        y <- 
                        convertTo
                        (sce, 
                        type=
                        "edgeR"
                        )
                    


*edgeR* uses negative binomial (NB) distributions to model the read/UMI counts for each sample. We estimate the NB dispersion parameter that quantifies the biological variability in expression across cells in the same cluster. Large dispersion estimates above 0.5 are often observed in scRNA-seq data due to technical noise, in contrast to bulk data where values of 0.05–0.2 are more typical. We then use the design matrix to fit a NB GLM to the counts for each gene ( McCarthy *et al.*, 2012).



                        y <- 
                        estimateDisp
                        (y, de.design)

                        fit <- 
                        glmFit
                        (y, de.design)

                        summary
                        (y$tagwise.dispersion)
                    




                        ##    Min. 1st Qu.  Median    Mean 3rd Qu.      Max.
## 0.04733 0.35370 0.64530 1.28600 1.32400 102.40000
                    


We assume that one of the clusters corresponds to our subpopulation of interest. Each gene is tested for DE between the chosen cluster and every other cluster in the dataset. We demonstrate this below for cluster 1, though the same process can be applied to any other cluster by changing chosen.clust.



                        result.logFC <- result.PValue <- 
                        list
                        ()

                        chosen.clust <- 
                        which
                        (
                        levels
                        (cluster)==
                        "1"
                        ) 
                        # character, as ’cluster’ is a factor.

                        for (clust in 
                        seq_len
                        (
                        nlevels
                        (cluster))) {
    
                        if (clust==chosen.clust) { next }
    
                        contrast <- 
                        numeric
                        (
                        ncol
                        (de.design))
    
                        contrast[chosen.clust] <- 
                        1
    
                        contrast[clust] <- -
                        1
    
                        res <- 
                        glmLRT
                        (fit, 
                        contrast=
                        contrast)
    
                        con.name <- 
                        paste0
                        (
                        ’vs.’
                        , 
                        levels
                        (cluster)[clust])
    
                        result.logFC[[con.name]] <- res$table$logFC
    
                        result.PValue[[con.name]] <- res$table$PValue

                        }
                    


Potential marker genes are identified by taking the top set of DE genes from each pairwise comparison between clusters. We arrange the results into a single output table that allows a marker set to be easily defined for a user-specified size of the top set. For example, to construct a marker set from the top 10 genes of each comparison, one would filter marker.set to retain rows with Top less than or equal to 10.



                        collected.ranks <- 
                        lapply
                        (result.PValue, rank, 
                        ties=
                        "first"
                        )

                        min.rank <- 
                        do.call
                        (pmin, collected.ranks)

                        marker.set <- 
                        data.frame
                        (
                        Top=
                        min.rank, 
                        Gene=rownames
                        (y),
    
                        logFC=do.call
                        (cbind, result.logFC), 
                        stringsAsFactors=
                        FALSE
                        )

                        marker.set <- marker.set[
                        order
                        (marker.set$Top),]

                        head
                        (marker.set, 
                        10
                        )
                    




                        ##     Top    Gene  logFC.vs.2  logFC.vs.3 logFC.vs.4 logFC.vs.5 logFC.vs.6 logFC.vs.7
## 26    1  Gm9846 -2.69173561 -0.89238306 -4.2332332 -1.0222698 -0.5414615 -2.5287437
## 223   1 Slc32a1  0.09461874 -0.04485368  0.1585265 -4.5682143 -1.4174543 -0.2546009
## 297   1   Cspg5 -1.30778951 -2.54296437 -1.5771899 -1.9881673 -1.4086953 -5.0830952
## 298   1    Syt1  2.78822084 -0.25850578  1.4804092 -0.8895181  0.3458730  1.8327007
## 862   1   Mef2c -1.08816401 -4.45879597 -2.9639706 -2.7639706 -2.9780931 -0.8413323
## 2563  1    Scd2 -4.45332845 -0.26021806 -1.0034850  0.1048065 -2.7348760 -3.4221061
## 260   2   Rcan2 -3.22472364 -3.05410260 -2.1732655 -4.5132580 -2.6087020 -0.9949232
## 309   2   Ndrg4  3.83886951 -0.34125245  2.6623976 -0.9701018  0.3516469  2.8775459
## 763   2     Clu -1.59785766 -2.42881333 -2.7317868 -1.9346444 -0.8799791 -6.1547824
## 963   2   Ncald -2.87305577 -4.43604787 -2.1004299 -4.5752214 -3.5526851 -1.5981341
                    


**Figure 26.**
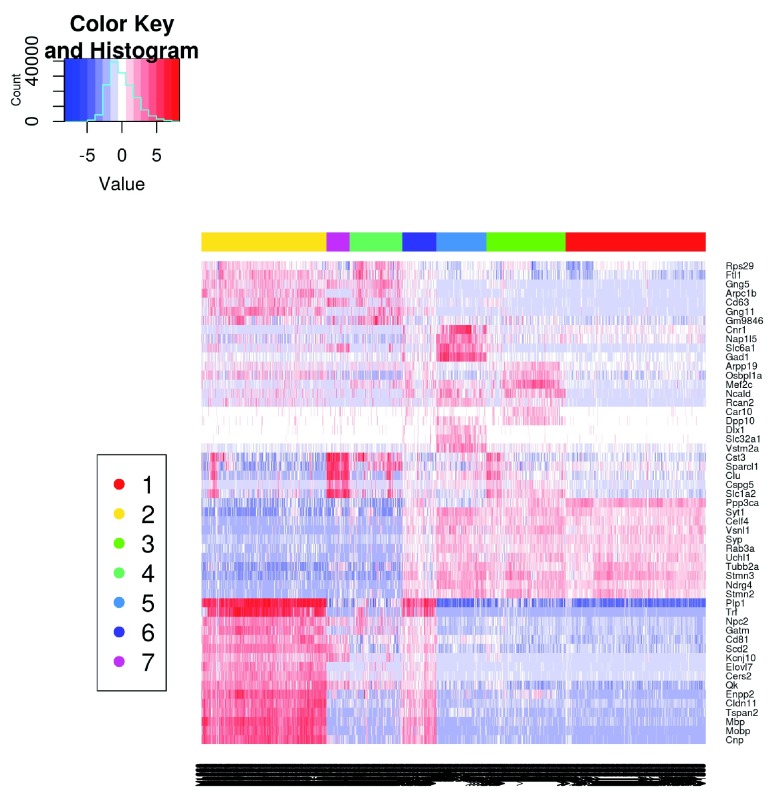
Heatmap of mean-centred normalized and corrected log-expression values for the top set of markers for cluster 1 in the brain dataset. Column colours represent the cluster to which each cell is assigned, as indicated by the legend.

We save the list of candidate marker genes for further examination. We also examine their expression profiles to verify that the DE signature is robust. Figure 26 indicates that most of the top markers have strong and consistent up- or downregulation in cells of cluster 1 compared to some or all of the other clusters. Thus, cells from the subpopulation of interest can be identified as those that express the upregulated markers and do not express the downregulated markers.



                        write.table
                        (marker.set, 
                        file=
                        "brain_marker_1.tsv"
                        , 
                        sep=
                        "\t"
                        , 
                        quote=
                        FALSE
                        , 
                        col.names=
                        NA
                        )

                        top.markers <- marker.set$Gene[marker.set$Top <= 
                        10
                        ]

                        top.exprs <- 
                        norm_exprs
                        (sce)[top.markers,,drop=
                        FALSE
                        ]

                        heat.vals <- top.exprs - 
                        rowMeans
                        (top.exprs)

                        heatmap.2
                        (heat.vals, 
                        col=
                        bluered, 
                        symbreak=
                        TRUE
                        , 
                        trace=
                        ’none’
                        , 
                        cexRow=
                        0.6
                        ,
    
                        ColSideColors=
                        clust.col[my.clusters], 
                        Colv=as.dendrogram
                        (my.tree), 
                        dendrogram=
                        ’none’
                        )

                        legend
                        (
                        "bottomleft"
                        , 
                        col=
                        clust.col, 
                        legend=sort
                        (
                        unique
                        (my.clusters)), 
                        pch=
                        16
                        )
                    


Many of the markers in Figure 26 are not uniquely up- or downregulated in the chosen cluster. Testing for unique DE tends to be too stringent as it overlooks important genes that are expressed in two or more clusters. For example, in a mixed population of CD4 ^+^-only, CD8 ^+^-only, double-positive and double-negative T cells, neither *Cd4* or *Cd8* would be detected as subpopulation-specific markers because each gene is expressed in two subpopulations. With our approach, both of these genes will be picked up as candidate markers as they will be DE between at least one pair of subpopulations. A combination of markers can then be chosen to characterize a subpopulation, which is more flexible than trying to find uniquely DE genes.

It must be stressed that the *p*-values computed here cannot be interpreted as measures of significance. This is because the clusters have been empirically identified from the data. *edgeR* does not account for the uncertainty and stochasticity in clustering, which means that the *p*-values are much lower than they should be. As such, these *p*-values should only be used for ranking candidate markers for follow-up studies. However, this is not a concern in other analyses where the groups are pre-defined. For such analyses, the FDR-adjusted *p*-value can be directly used to define significant genes for each DE comparison, though some care may be required to deal with plate effects ( Hicks *et al.*, 2015; Tung *et al.*, 2016).

### Additional comments

Having completed the basic analysis, we save the SCESet object with its associated data to file. This is especially important here as the brain dataset is quite large. If further analyses are to be performed, it would be inconvenient to have to repeat all of the pre-processing steps described above.



                        saveRDS
                        (
                        file=
                        "brain_data.rds"
                        , sce)
                    


## Alternative parameter settings and strategies

### Normalizing based on spike-in coverage

Scaling normalization strategies for scRNA-seq data can be broadly divided into two classes. The first class assumes that there exists a subset of genes that are not DE between samples, as previously described. The second class uses the fact that the same amount of spike-in RNA was added to each cell. Differences in the coverage of the spike-in transcripts can only be due to cell-specific biases, e.g., in capture efficiency or sequencing depth. Scaling normalization is then applied to equalize spike-in coverage across cells.

The choice between these two normalization strategies depends on the biology of the cells and the features of interest. If the majority of genes are expected to be DE and there is no reliable house-keeping set, spike-in normalization may be the only option for removing cell-specific biases. Spike-in normalization should also be used if differences in the total RNA content of individual cells are of interest. In any particular cell, an increase in the amount of endogenous RNA will not increase spike-in coverage (with or without library quantification). Thus, the former will not be represented as part of the bias in the latter, which means that the effects of total RNA content on expression will not be removed upon scaling. With non-DE normalization, an increase in RNA content will systematically increase the expression of all genes in the non-DE subset, such that it will be treated as bias and removed.

We demonstrate the use of spike-in normalization on a dataset involving different cell types – namely, mouse embryonic stem cells (mESCs) and mouse embryonic fibroblasts (MEFs) ( Islam *et al.*, 2011). The count table was obtained from NCBI GEO as a supplementary file under the accession GSE29087 ( http://www.ncbi.nlm.nih.gov/geo/query/acc.cgi?acc=GSE29087). We load the counts into R and specify the rows corresponding to spike-in transcripts. The negative control wells do not contain any cells and are useful for quality control but need to be removed prior to downstream analysis.



                        counts <- 
                        read.table
                        (
                        "GSE29087_L139_expression_tab.txt.gz"
                        , 
                        colClasses=c
                        (
                        list
                        (
                        "character"
                        ,
    
                        NULL
                        , 
                        NULL
                        , 
                        NULL
                        , 
                        NULL
                        , 
                        NULL
                        , 
                        NULL
                        ), 
                        rep
                        (
                        "integer"
                        , 
                        96
                        )), 
                        skip=
                        6
                        , 
                        sep=
                        ’\t’
                        , 
                        row.names=
                        1
                        )

                        sce <- 
                        newSCESet
                        (
                        countData=
                        counts)

                        sce$grouping <- 
                        rep
                        (
                        c
                        (
                        "mESC"
                        , 
                        "MEF"
                        , 
                        "Neg"
                        ), 
                        c
                        (
                        48
                        , 
                        44
                        , 
                        4
                        ))

                        sce <- sce[,sce$grouping!=
                        "Neg"
                        ] 
                        # Removing negative control wells.

                        sce <- 
                        calculateQCMetrics
                        (sce, 
                        feature_controls=list
                        (
                        spike=grep
                        (
                        "SPIKE"
                        , 
                        rownames
                        (counts))))

                        isSpike
                        (sce) <- 
                        "spike"
                    


We then apply the computeSpikeFactors method to estimate size factors for all cells. This method computes the total count over all spike-in transcripts in each cell, and calculates size factors to equalize the total spike-in count across cells. Here, we set general.use=TRUE as we intend to apply the spike-in factors to all counts.



                        sce <- 
                        computeSpikeFactors
                        (sce, 
                        general.use=
                        TRUE
                        )
                    


Applying normalize will use the spike-in-based size factors to compute normalized log-expression values. Unlike in the previous analyses, we do not have to set separate size factors for the spike-in transcripts. This is because the relevant factors are already being used for all genes and spike-in transcripts when general.use=TRUE. (The exception is if the experiment uses multiple spike-in sets that behave differently and need to be normalized separately.)



                        sce <- 
                        normalize
                        (sce)
                    


**Figure 27.**
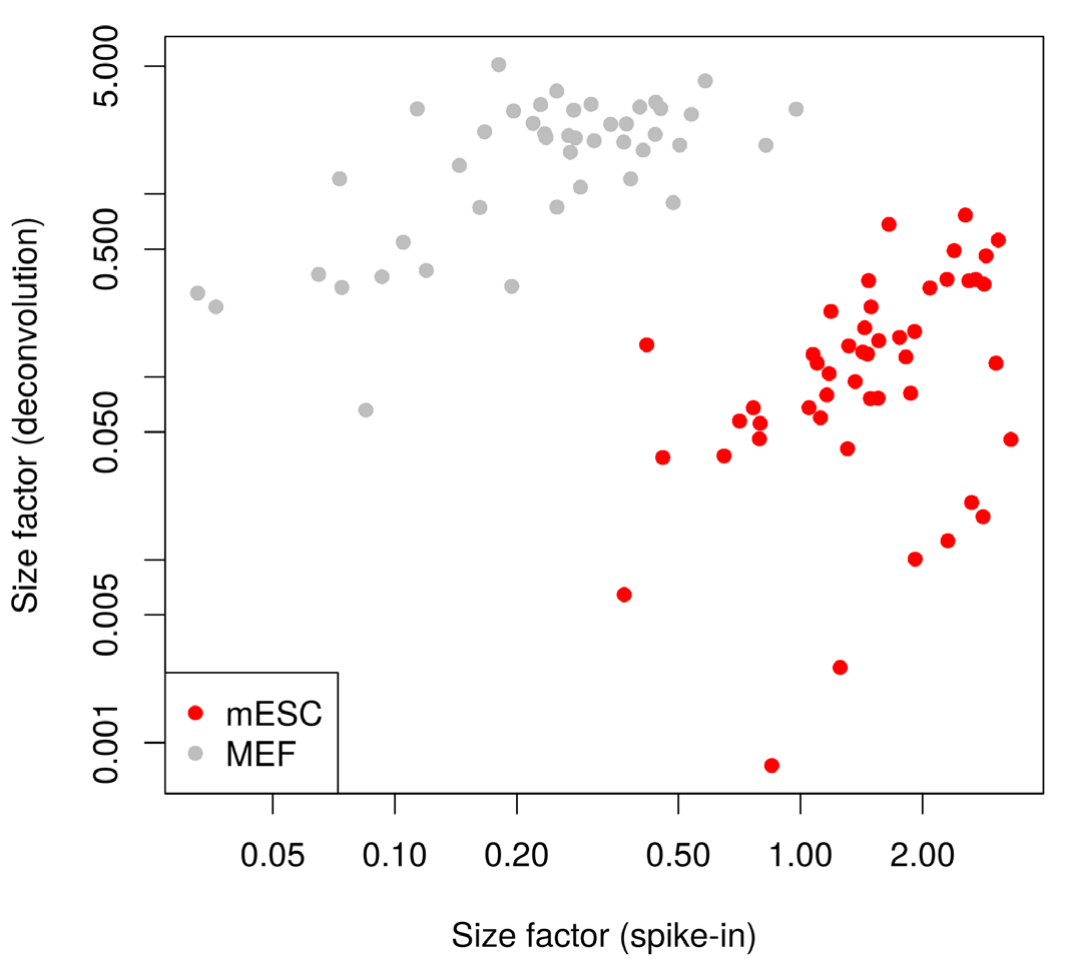
Size factors from spike-in normalization, plotted against the size factors from deconvolution for all cells in the mESC/MEF dataset. Axes are shown on a log-scale, and cells are coloured according to their identity. Deconvolution size factors were computed with small pool sizes owing to the low number of cells of each type.



For comparison, we also compute the deconvolution size factors and plot them against the spike-in factors. We observe a negative correlation between the two sets of values ( Figure 27). This is because MEFs contain more endogenous RNA, which reduces the relative spike-in coverage in each library (thereby decreasing the spike-in size factors) but increases the coverage of endogenous genes (thus increasing the deconvolution size factors). If the spike-in size factors were applied to the counts, the expression values in MEFs would be scaled up while expression in mESCs would be scaled down. However, the opposite would occur if deconvolution size factors were used.

                        colours <- 
                        c
                        (
                        mESC=
                        "red"
                        , 
                        MEF=
                        "grey"
                        )

                        deconv.sf <- 
                        computeSumFactors
                        (sce, 
                        sf.out=
                        TRUE
                        , 
                        cluster=
                        sce$grouping, 
                        sizes=
                        1
                        :
                        4
                        *
                        10
                        )

                        plot
                        (
                        sizeFactors
                        (sce), deconv.sf, 
                        col=
                        colours[sce$grouping], 
                        pch=
                        16
                        , 
                        log=
                        "xy"
                        ,
    
                        xlab=
                        "Size factor (spike-in)"
                        , 
                        ylab=
                        "Size factor (deconvolution)"
                        )

                        legend
                        (
                        "bottomleft"
                        , 
                        col=
                        colours, 
                        legend=names
                        (colours), 
                        pch=
                        16
                        )
                    


Whether or not total RNA content is relevant – and thus, the choice of normalization strategy – depends on the biological hypothesis. In the HSC and brain analyses, variability in total RNA across the population was treated as noise and removed by non-DE normalization. This may not always be appropriate if total RNA is associated with a biological difference of interest. For example, Islam *et al.* (2011) observe a 5-fold difference in total RNA between mESCs and MEFs. Similarly, the total RNA in a cell changes across phases of the cell cycle ( Buettner *et al.*, 2015). Spike-in normalization will preserve these differences in total RNA content such that the corresponding biological groups can be easily resolved in downstream analyses.

### Blocking on the cell cycle phase

Cell cycle phase is usually uninteresting in studies focusing on other aspects of biology. However, the effects of cell cycle on the expression profile can mask other effects and interfere with the interpretation of the results. This cannot be avoided by simply removing cell cycle marker genes, as the cell cycle can affect a substantial number of other transcripts ( Buettner *et al.*, 2015). Rather, more sophisticated strategies are required, one of which is demonstrated below using data from a study of T Helper 2 (T _H_2) cells ( Mahata *et al.*, 2014). Buettner *et al.* (2015) have already applied quality control and normalized the data, so we can use them directly as log-expression values (accessible as Supplementary Data 1 of https://dx.doi.org/10.1038/nbt.3102).



                        library
                        (openxlsx)

                        incoming <- 
                        read.xlsx
                        (
                        "nbt.3102-S7.xlsx"
                        , 
                        sheet=
                        1
                        , 
                        rowNames=
                        TRUE
                        )

                        incoming <- incoming[,!
                        duplicated
                        (
                        colnames
                        (incoming))] 
                        # Remove duplicated genes.

                        sce <- 
                        newSCESet
                        (
                        exprsData=t
                        (incoming), 
                        logged=
                        TRUE
                        )
                    


**Figure 28.**
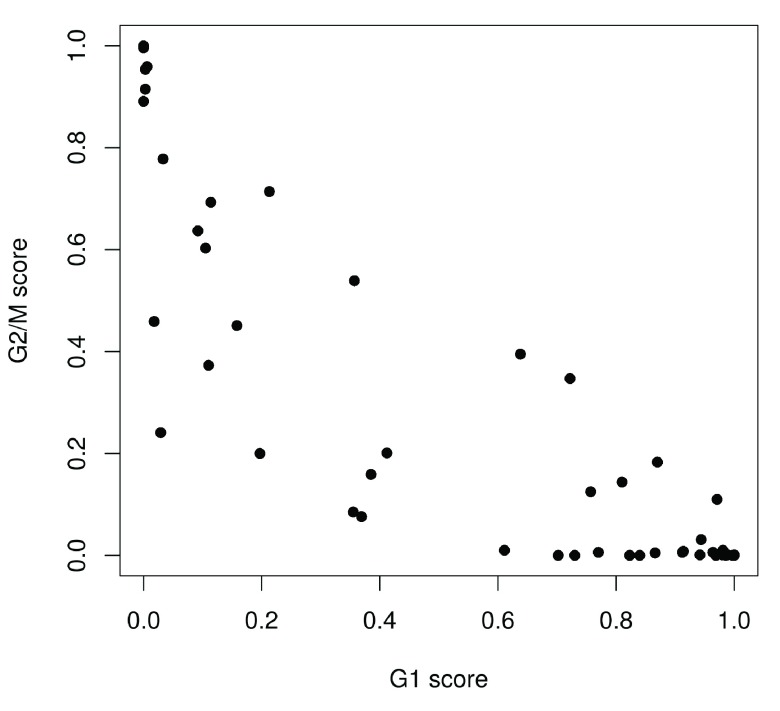
Cell cycle phase scores from applying the pair-based classifier on the T _H_2 dataset, where each point represents a cell.



We empirically identify the cell cycle phase using the pair-based classifier in cyclone. The majority of cells in Figure 28 seem to lie in G1 phase, with small numbers of cells in the other phases.

                        anno <- 
                        select
                        (org.Mm.eg.db, 
                        keys=rownames
                        (sce), 
                        keytype=
                        "SYMBOL"
                        , 
                        column=
                        "ENSEMBL"
                        )

                        ensembl <- anno$ENSEMBL[
                        match
                        (
                        rownames
                        (sce), anno$SYMBOL)]

                        assignments <- 
                        cyclone
                        (sce, mm.pairs, 
                        gene.names=
                        ensembl, 
                        assay=
                        "exprs"
                        )

                        plot
                        (assignments$score$G1, assignments$score$G2M, 
                        xlab=
                        "G1 score"
                        , 
                        ylab=
                        "G2/M score"
                        , 
                        pch=
                        16
                        )
                    


We can block directly on the phase scores in downstream analyses. This is more graduated than using a strict assignment of each cell to a specific phase, as the magnitude of the score considers the uncertainty of the assignment. The phase covariates in the design matrix will absorb any phase-related effects on expression such that they will not affect estimation of the effects of other experimental factors. Users should also ensure that the phase score is not confounded with other factors of interest. For example, model fitting is not possible if all cells in one experimental condition are in one phase, and all cells in another condition are in a different phase.



                        design <- 
                        model.matrix
                        (
                            ^~^ G1 + G2M, assignments$score)

                        fit.block <- 
                        trendVar
                        (sce, 
                        use.spikes=
                        NA
                        , 
                        trend=
                        "loess"
                        , 
                        design=
                        design)

                        dec.block <- 
                        decomposeVar
                        (sce, fit.block)
                    


**Figure 29.**
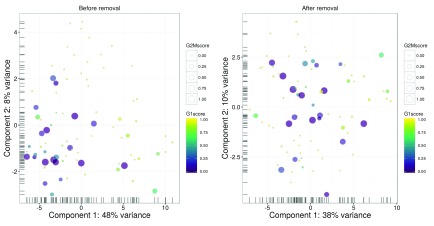
PCA plots before (left) and after (right) removal of the cell cycle effect in the T _H_2 dataset. Each cell is represented by a point with colour and size determined by the G1 and G2/M scores, respectively. Only HVGs were used to construct each plot.



For analyses that do not use design matrices, we remove the cell cycle effect directly from the expression values using removeBatchEffect. The result of this procedure is visualized with some PCA plots in Figure 29. Before removal, the distribution of cells along the first two principal components is strongly associated with their G1 and G2/M scores. This is no longer the case after removal, which suggests that the cell cycle effect has been mitigated.

                        # Finding HVGs without blocking on phase score.

                        fit <- 
                        trendVar
                        (sce, 
                        use.spikes=
                        NA
                        , 
                        trend=
                        "loess"
                        )

                        dec <- 
                        decomposeVar
                        (sce, fit)
top.hvgs <- 
                        which
                        (dec$FDR <= 
                        0.05 
                        & dec$bio >= 
                        0.5
                        )
sce$G1score <- assignments$score$G1
sce$G2Mscore <- assignments$score$G2M
out <- 
                        plotPCA
                        (sce, 
                        feature_set=
                        top.hvgs, 
                        colour_by=
                        "G1score"
                        , 
                        size_by=
                        "G2Mscore"
                        ) +
    fontsize + 
                        ggtitle
                        (
                        "Before removal"
                        )


                        # Using HVGs after blocking on the phase score.

                        top.hvgs2 <- 
                        which
                        (dec.block$FDR <= 
                        0.05 
                        & dec.block$bio >= 
                        0.5
                        )

                        norm_exprs
                        (sce) <- 
                        removeBatchEffect
                        (
                        exprs
                        (sce), 
                        covariates=
                        assignments$score[,
                        c
                        (
                        "G1"
                        , 
                        "G2M"
                        )])
out2 <- 
                        plotPCA
                        (sce, 
                        exprs_values=
                        "norm_exprs"
                        , 
                        feature_set=
                        top.hvgs2, 
                        colour_by=
                        "G1score"
                        ,
    
                        size_by=
                        "G2Mscore"
                        ) + fontsize + 
                        ggtitle
                        (
                        "After removal"
                        )

                        multiplot
                        (out, out2, 
                        cols=
                        2
                        )
                    


**Figure 30.**
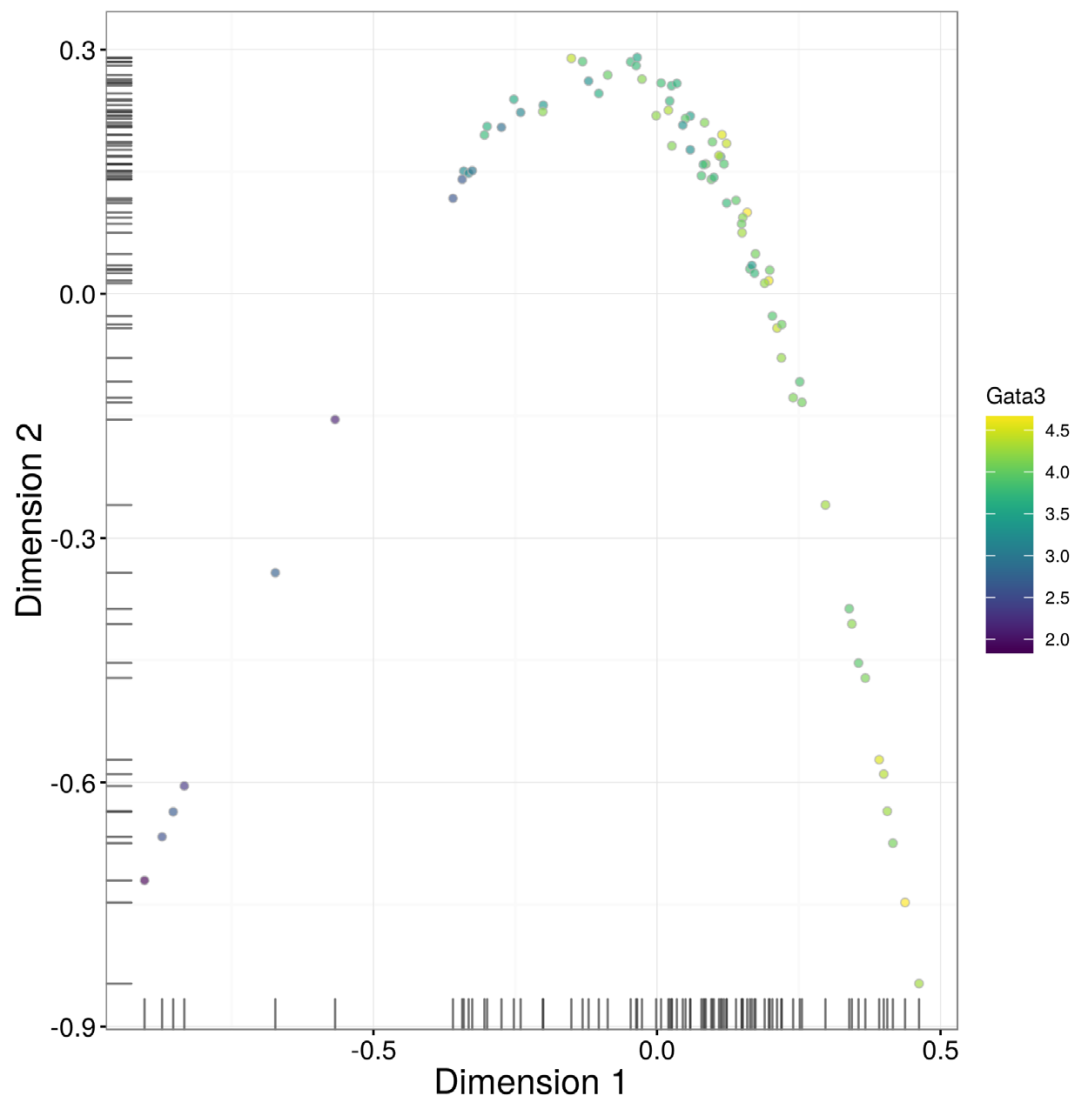
A diffusion map for the T _H_2 dataset, where each cell is coloured by its expression of *Gata3*.



As an aside, this dataset contains cells at various stages of differentiation ( Mahata *et al.*, 2014). This is an ideal use case for diffusion maps which perform dimensionality reduction along a continuous process. In Figure 30, cells are arranged along a trajectory in the low-dimensional space. The first diffusion component is likely to correspond to T _H_2 differentiation, given that a key regulator *Gata3* ( Zhu *et al.*, 2006) changes in expression from left to right.

                        plotDiffusionMap
                        (sce, 
                        exprs_values=
                        "norm_exprs"
                        , 
                        colour_by=
                        "Gata3"
                        ) + fontsize
                    


### Extracting annotation from Ensembl identifiers

Feature-counting tools typically report genes in terms of standard identifiers from Ensembl or Entrez. These identifiers are used as they are unambiguous and highly stable. However, they are difficult to interpret compared to the gene symbols which are more commonly used in the literature. We can easily convert from one to the other using annotation packages like *org.Mm.eg.db*. This is demonstrated below for Ensembl identifiers in a mESC dataset ( Kolodziejczyk *et al.*, 2015) obtained from http://www.ebi.ac.uk/teichmann-srv/espresso. The select call extracts the specified data from the annotation object, and the match call ensures that the first gene symbol is used if multiple symbols correspond to a single Ensembl identifier.



                        incoming <- 
                        read.table
                        (
                        "counttable_es.csv"
                        , 
                        header=
                        TRUE
                        , 
                        row.names=
                        1
                        )
my.ids <- 
                        rownames
                        (incoming)
anno <- 
                        select
                        (org.Mm.eg.db, 
                        keys=
                        my.ids, 
                        keytype=
                        "ENSEMBL"
                        , 
                        column=
                        "SYMBOL"
                        )
anno <- anno[
                        match
                        (my.ids, anno$ENSEMBL),]

                        head
                        (anno)
                    




                        ## 		ENSEMBL SYMBOL
## 1 ENSMUSG00000000001  Gnai3
## 2 ENSMUSG00000000003   Pbsn
## 3 ENSMUSG00000000028  Cdc45
## 4 ENSMUSG00000000031    H19
## 5 ENSMUSG00000000037  Scml2
## 6 ENSMUSG00000000049   Apoh
                    


To identify which rows correspond to mitochondrial genes, we need to use extra annotation describing the genomic location of each gene. For Ensembl, this involves using the *TxDb.Mmusculus.UCSC.mm10.ensGene* package.



                        library
                        (TxDb.Mmusculus.UCSC.mm10.ensGene)
location <- 
                        select
                        (TxDb.Mmusculus.UCSC.mm10.ensGene, 
                        keys=
                        my.ids,
     
                        column=
                        "CDSCHROM"
                        , 
                        keytype=
                        "GENEID"
                        )
location <- location[
                        match
                        (my.ids, location$GENEID),]
is.mito <- location$CDSCHROM == 
                        "chrM" 
                        & !
                        is.na
                        (location$CDSCHROM)

                        sum
                        (is.mito)
                    




                        ## [1] 13
                    


Identification of rows that correspond to spike-in transcripts is much easier, given that the ERCC spike-ins were used.



                        is.spike <- 
                        grepl
                        (
                        "^ERCC"
                        , my.ids)

                        sum
                        (is.spike)
                    




                        ## [1] 92
                    


All of this information can be consolidated into a SCESet object for further manipulation. Alternatively, annotation from BioMart resources can be directly added to the object using the getBMFeatureAnnos function from *scater*.



                        anno <- anno[,-
                        1
                        ,drop=
                        FALSE
                        ]

                        rownames
                        (anno) <- my.ids
sce <- 
                        newSCESet
                        (
                        countData=
                        incoming, 
                        featureData=AnnotatedDataFrame
                        (anno))
sce <- 
                        calculateQCMetrics
                        (sce, 
                        feature_controls=list
                        (
                        ERCC=
                        is.spike))

                        isSpike
                        (sce) <- 
                        "ERCC"
                    


We filter out rows that do not correspond to endogenous genes or spike-in transcripts. This will remove rows containing mapping statistics such as the number of unaligned or unassigned reads, which would be misleading if treated as gene expression values. The object is then ready for downstream analyses as previously described.



                        sce <- sce[
                        grepl
                        (
                        "ENSMUS"
                        , 
                        rownames
                        (sce)) | 
                        isSpike
                        (sce),]

                        dim
                        (sce)
                    




                        ## Features Samples
##    38653     704
                    


## Conclusions

This workflow provides a step-by-step guide for performing basic analyses of single-cell RNA-seq data in R. It provides instructions for a number of low-level steps such as quality control, normalization, cell cycle phase assignment, data exploration, HVG and marker gene detection, and clustering. This is done with a number of different datasets to provide a range of usage examples. The workflow is modular so individual steps can be substituted with alternative methods according to user preferences. In addition, the processed data can be easily used for higher-level analyses with other Bioconductor packages. We anticipate that this workflow will assist readers in assembling analyses of their own scRNA-seq data.

## Software availability

All software packages used in this workflow are publicly available from the Comprehensive R Archive Network ( https://cran.r-project.org) or the Bioconductor project ( http://bioconductor.org). The specific version numbers of the packages used are shown below, along with the version of the R installation. Version numbers of all Bioconductor packages correspond to release version 3.4 of the Bioconductor project. Users can install all required packages and execute the workflow by following the instructions at https://www.bioconductor.org/help/workflows/simpleSingleCell. The workflow takes less than an hour to run on a desktop computer with 8 GB of memory.



                    sessionInfo
                    ()
                




                    ## R version 3.3.1 Patched (2016-10-17 r71532)
## Platform: x86_64-pc-linux-gnu (64-bit)
## Running under: Ubuntu 14.04.5 LTS
##
## locale:
##  [1] LC_CTYPE=en_GB.UTF-8 	  LC_NUMERIC=C 		     LC_TIME=en_GB.UTF-8
##  [4] LC_COLLATE=en_GB.UTF-8 	  LC_MONETARY=en_GB.UTF-8    LC_MESSAGES=en_GB.UTF-8
##  [7] LC_PAPER=en_GB.UTF-8 	  LC_NAME=C 		     LC_ADDRESS=C
## [10] LC_TELEPHONE=C 	   	  LC_MEASUREMENT=en_GB.UTF-8 LC_IDENTIFICATION=C
##
## attached base packages:
## [1] stats4 	 parallel stats     graphics   grDevices utils    datasets methods base
##
## other attached packages:
##  [1] TxDb.Mmusculus.UCSC.mm10.ensGene_3.4.0 GenomicFeatures_1.26.0
##  [3] GenomicRanges_1.26.0 		       GenomeInfoDb_1.10.0
##  [5] openxlsx_3.0.0 		       	       edgeR_3.16.0
##  [7] dynamicTreeCut_1.63-1 		       limma_3.30.0
##  [9] gplots_3.0.1 		       	       RBGL_1.50.0
## [11] graph_1.52.0 		       	       org.Mm.eg.db_3.4.0
## [13] AnnotationDbi_1.36.0 		       IRanges_2.8.0
## [15] S4Vectors_0.12.0 		       scran_1.2.0
## [17] scater_1.2.0 		       	       ggplot2_2.1.0
## [19] Biobase_2.34.0 		       	       BiocGenerics_0.20.0
## [21] gdata_2.17.0 		       	       R.utils_2.4.0
## [23] R.oo_1.20.0 		       	       R.methodsS3_1.7.1
## [25] destiny_2.0.0 		       	       mvoutlier_2.0.6
## [27] sgeostat_1.0-27 		       Rtsne_0.11
## [29] BiocParallel_1.8.0 		       knitr_1.14
## [31] BiocStyle_2.2.0 		       	       
##
## loaded via a namespace (and not attached):
##   [1] Hmisc_3.17-4 		     RcppEigen_0.3.2.9.0 	 plyr_1.8.4
##   [4] igraph_1.0.1 		     sp_1.2-3 	 		 shinydashboard_0.5.3
##   [7] splines_3.3.1 		     digest_0.6.10 	 	 htmltools_0.3.5
##  [10] viridis_0.3.4 		     magrittr_1.5 	 	 cluster_2.0.5
##  [13] Biostrings_2.42.0 	     matrixStats_0.51.0  	 xts_0.9-7
##  [16] colorspace_1.2-7 	     rrcov_1.4-3 	 	 dplyr_0.5.0
##  [19] RCurl_1.95-4.8 	     tximport_1.2.0 	 	 lme4_1.1-12
##  [22] survival_2.39-5 	     zoo_1.7-13 	 	 gtable_0.2.0
##  [25] XVector_0.14.0 	     zlibbioc_1.20.0 	         MatrixModels_0.4-1
##  [28] car_2.1-3 	             kernlab_0.9-25 	  	 prabclus_2.2-6
##  [31] DEoptimR_1.0-6 	     SparseM_1.72 	  	 VIM_4.6.0
##  [34] scales_0.4.0 		     mvtnorm_1.0-5 	  	 DBI_0.5-1
##  [37] GGally_1.2.0 		     Rcpp_0.12.7 	  	 sROC_0.1-2
##  [40] xtable_1.8-2 		     laeken_0.4.6 	  	 foreign_0.8-67
##  [43] proxy_0.4-16 		     mclust_5.2 	         Formula_1.2-1
##  [46] vcd_1.4-3 		     FNN_1.1 	  	         RColorBrewer_1.1-2
##  [49] fpc_2.1-10 	             acepack_1.3-3.3 	  	 modeltools_0.2-21
##  [52] reshape_0.8.5 		     XML_3.98-1.4 	  	 flexmix_2.3-13
##  [55] nnet_7.3-12 		     locfit_1.5-9.1 	  	 labeling_0.3
##  [58] reshape2_1.4.1 	     munsell_0.4.3 	  	 tools_3.3.1
##  [61] RSQLite_1.0.0 		     pls_2.5-0 	  	 	 evaluate_0.10
##  [64] stringr_1.1.0 		     cvTools_0.3.2 	  	 robustbase_0.92-6
##  [67] caTools_1.17.1 	     nlme_3.1-128 	   	 mime_0.5
##  [70] quantreg_5.29 	             formatR_1.4 	   	 biomaRt_2.30.0
##  [73] pbkrtest_0.4-6 	     beeswarm_0.2.3 	   	 e1071_1.6-7
##  [76] statmod_1.4.26 	     smoother_1.1    	         tibble_1.2
##  [79] robCompositions_2.0.2       pcaPP_1.9-61   	         stringi_1.1.2
##  [82] lattice_0.20-34 	     trimcluster_0.1-2   	 Matrix_1.2-7.1
##  [85] nloptr_1.0.4 	             lmtest_0.9-34   	 	 data.table_1.9.6
##  [88] bitops_1.0-6 		     rtracklayer_1.34.0   	 httpuv_1.3.3
##  [91] R6_2.2.0                    latticeExtra_0.6-28         KernSmooth_2.23-15
##  [94] gridExtra_2.2.1 	     vipor_0.4.4                 boot_1.3-18
##  [97] MASS_7.3-45 		     gtools_3.5.0     		 assertthat_0.1
## [100] SummarizedExperiment_1.4.0  chron_2.3-47    		 rhdf5_2.18.0
## [103] rjson_0.2.15 		     GenomicAlignments_1.10.0    Rsamtools_1.26.0
## [106] diptest_0.75-7              mgcv_1.8-15                 grid_3.3.1
## [109] rpart_4.1-10                class_7.3-14                minqa_1.2.4
## [112] TTR_0.23-1                  scatterplot3d_0.3-37        shiny_0.14.1
## [115] ggbeeswarm_0.5.0
                

