# Supplementary material for: A step-by-step workflow for low-level analysis of single-cell RNA-seq data with Bioconductor
Source: F1000Res. 2016 Oct 31;5:2122. Originally published 2016 Aug 31. [Version 2] doi: 10.12688/f1000research.9501.2 (PMC5112579; doi:10.12688/f1000research.9501.2)
Supplement: Peer review history

# Peer review: Referee response for version 2

**DOI:** 10.5256/f1000research.10712.r17325

**Published:** 2016-11-29

**Authors:** Diana H.P. Low

**Affiliations:** 1 Institute of Molecular and Cell Biology, Agency for Science, Technology and Research, Singapore, Singapore

The revised manuscript has satisfactorily addressed my largest concern about the reproducibility of the workflow by updating the code, and stating the dependence on Bioconductor 3.4.

 While I understand that this workflow is meant to work under version 3.4, it is important to mention these information in the manuscript regardless of the time of publication, especially if one expects that there might be casual users of R (at this point I would echo the sentiments of Hongkai Ji regarding installation convenience). Working with development versions of R - albeit awaiting its release at that point in time - is a slight danger in itself, unless the workflow is only relying on packages that the authors have themselves developed. However, these are but minor issues.

 I thank the authors for creating this documentation that should provide a sufficient launchpad for those getting into single-cell analyses. I hope the authors will continue to improve upon the components as the field matures.

I have read this submission. I believe that I have an appropriate level of expertise to confirm that it is of an acceptable scientific standard.

# Peer review: Referee response for version 2

**DOI:** 10.5256/f1000research.10712.r17327

**Published:** 2016-11-21

**Authors:** Hongkai Ji

**Affiliations:** 1 Department of Biostatistics, Johns Hopkins Bloomberg School of Public Health, Baltimore, MD, USA

In this revised manuscript, the authors have satisfactorily answered my previous questions. Overall, the workflow provides a useful step-by-step instruction on how to analyze scRNA-seq data, and it is worthwhile to be documented in the literature. The authors explained why they hesitated to provide the whole pipeline through an R file and a graphical user interface. While their explanations are reasonable, convenience in installation and data exploration will greatly help many users. Therefore, I hope they could add/improve those components as they continue to develop this workflow.

I have read this submission. I believe that I have an appropriate level of expertise to confirm that it is of an acceptable scientific standard.

# Peer review: Referee response for version 2

**DOI:** 10.5256/f1000research.10712.r17329

**Published:** 2016-11-11

**Authors:** Andrew McDavid

**Affiliations:** 1 Department of Biostatistics and Computational Biology, University of Rochester, Rochester, NY, USA

In the 2nd version, Lun and colleagues have updated the workflow for the 3.4 release of Bioconductor and provided a tool to install all the needed dependencies to replicate the workflow. This eases reproducibility and addresses my first and largest concern.

 The authors have not modified the article in response to my other comments, but rather offer a rebuttal. I acknowledge that some of the issues I raise are probably too large to be solved in a vignette, but nonetheless potential users of this workflow should be aware of them. I reply below:

**1b.** Inheriting from a `SummarizedExperiment` not only allows storage in sparse formats, which as indicated in rebuttal may not be terribly applicable for scRNAseq, but also allows analysis of data that is too big to fit into memory, by storing the data on-disk in HDF5 format. Adopting `SummarizedExperiment` as a container would future-proof this software in the event that data sets do get too large to fit into memory of conventional machines. A back-of-the envelop calculation suggests this won't be an issue until data sets are on the order of 10 ^5^ cells, and require on the order of gigabytes of memory to store. Conducting analysis on big-memory machines might delay this issue indefinitely.

**2.**  Although feature counting is an approach used by many studies, I do not know that it has been shown to provide equivalent estimates to EM-based transcript quantitation (and would welcome a reference otherwise!) Because feature counting throws away multi-mapped reads, at a minimum it is an inefficient use (scarce, costly) data. I would not characterize it generally as "conservative," either, unless the reads are discarded uniformly at random from all possible transcripts, and only single isoforms are of interest. If uniformity doesn't hold, or multiple isoforms are present, feature counting distorts the relative abundances of genes, and even sample-to-sample comparisons of the same gene (e.g. Figures S1 and S4 in [1]). Additionally, modern quantitation programs do much more than account for multi-mapping reads--they also can model biases in the sequencing chemistry and fragment start sites, and can be faster than alignment-based procedures. ^2,3^  I find analyst inertia an unconvincing reason not to adopt them.

**3. **I also agree that the plethora of alternate approaches is a quagmire for end-users at the moment. Lamentably there has been very little in the way of across-method comparison, and instead the methods have been developed around a specific data set, or two, and rarely compared to each other. More direct head-to-head comparisons would advance the field dramatically. In the meantime, *caveat calculator*.  

 I thank the authors for their effort in creating this tutorial. Having well-documented software and workflows will be critical for allowing the sorts of head-to-head comparisons I mention in [3].

I have read this submission. I believe that I have an appropriate level of expertise to confirm that it is of an acceptable scientific standard.

## References

[1] TrapnellCHendricksonDGSauvageauMGoffLRinnJLPachterL: Differential analysis of gene regulation at transcript resolution with RNA-seq. *Nat Biotechnol*.2013;31(1) : 10.1038/nbt.2450 46-53 10.1038/nbt.2450 23222703PMC3869392

[2] BrayNLPimentelHMelstedPPachterL: Near-optimal probabilistic RNA-seq quantification. *Nat Biotechnol*.2016;34(5) : 10.1038/nbt.3519 525-7 10.1038/nbt.3519 27043002

[3] PatroRMountSMKingsfordC: Sailfish enables alignment-free isoform quantification from RNA-seq reads using lightweight algorithms. *Nat Biotechnol*.2014;32(5) : 10.1038/nbt.2862 462-4 10.1038/nbt.2862 24752080PMC4077321

# Peer review: Referee response for version 1

**DOI:** 10.5256/f1000research.10234.r15986

**Published:** 2016-10-20

**Authors:** Antonio Rausell

**Affiliations:** 1 Clinical Bioinformatics laboratory, Imagine Institute, Paris Descartes University - Sorbonne Paris Cité, Paris, France

In the Software tool article “A step-by-step workflow for low-level analysis of single-cell RNA-seq data”, Lun, McCarthy and Marioni thoroughly describe a comprehensive pipeline for the low-level analysis of single-cell RNA-seq data. The article covers important topics such as the quality control of cells and genes, normalization of expression levels, control for technical factors and cell cycle, detection of highly variable genes, assessment of subpopulations of cells and associated differentially expressed genes. The workflow is illustrated in a number of datasets offering diverse scenarios that nicely guide the reader on the different criteria that may be adopted throughout the analysis. The manuscript is clearly presented, the quality of the code and figures is excellent and a great effort has been done to introduce complex questions in an easily accessible manner to a broad audience. Importantly, the authors discuss situations where it is difficult to provide a clear-cut recipe, and the need for experimental validation is stressed. Overall I think the article is an important contribution to the community and that it should quickly become a reference guide in the field.

 I report here a number of comments, questions and suggestions with the hope that they may contribute to improve an already excellent article:

 1.    In addition to the approaches proposed by the authors to detect low quality cells, I would suggest the readers the possibility of identifying outlier cells by performing a PCA on the normalized gene expression matrix restricted to protein-coding genes (e.g. using biotype annotations from Ensembl biomart). On the one hand, outlier cells will dominate the first principal components, which will show high percentage of variances simply accounting from the separation of the outlier from the compact cloud of “normal” cells. On the other hand, a PCA analysis could suggest keeping a cell whose relative similarity to the rest of the cells in a low-dimensional space seems rather normal, even if it could still have an allegedly "bad" quality metric.

 2.    As an additional quality control check for the cells, I would also suggest to assess whether the sequencing depth was generally deep enough for most of the cells, by inspecting for instance the saturation curve of the number of detected genes (or other features like the known exon-exon junctions) as a function of the fraction of down-sampled reads.

 3.    In the text it is proposed to filter out low-abundance genes, defined as “those with an average count below a filter threshold of 1." However, the average count is assessed before the normalization step. Would it be more meaningful to apply this filter on the normalized counts?

 4.    A priori it is difficult to rule out the possibility that the filtering of low-abundance genes could eventually hamper the identification of relevant genes in rare populations of cells. I would generally suggest being rather inclusive at this stage, especially when no clustering of single-cells has been done yet, so that it would still be possible to check whether e.g. the few cells expressing some genes -even if at low levels- are actually forming a distinctive and biologically relevant cluster.

 5.    Authors propose as an alternative approach to gene filtering, to select genes that have non-zero counts in at least n cells. As illustrated in Figure 6, the number of cells expressing a given gene may be modeled by its mean expression level. This was elegantly addressed in Shalek *et al*. (2014) through a likelihood ratio test comparing a null model -where all cells express a gene in a lognormal fashion- with an alternate model -where a gene is not expressed in a subpopulation of cells α (See section "Controlling for relationship between expression level and detection efficiency" in the supplementary material: http://www.nature.com/nature/journal/v510/n7505/extref/nature13437-s1.pdf). Genes for which the null model is rejected may be indicative of a subpopulation of cells not expressing the gene at a higher fraction than the one expected from technical noise (e.g. dropout events). I would suggest exploring such approach in order to avoid filtering out relevant genes due to a sharp threshold on the number of cells expressing it.

 6.    In the section “Filtering out low-abundance genes”, the sentence "This provides some more protection against genes with outlier expression patterns, i.e., strong expression in only one or two cells. Such outliers are typically uninteresting as they can arise from amplification artifacts that are not replicable across cells.[...]" would be better followed by setting alt.keep <- numcells >= 2 instead of >= 10

 7.    It would be interesting to complement Figure 7 and 18 with a second panel representing the correlation between size factors from deconvolution versus spike-in-specific size factors, as done in Figure 27. In the eventual case that a low correlation between them was found in a non-DE scenario, would it be advisable to neglect spike-ins from the analysis?

 8.    As pointed by the authors, spike-in molecules have been extensively used to infer the amount of variability in the expression levels of one gene that can be explained from technical noise (e.g. Brennecke *et al.*, 2013; Grün *et al.*, 2014; Islam *et al.*, 2014). Ding *et al.* (2015) went further on the applications of spike-in levels, by using them to explicitly remove technical noise and compute de-noised gene expression levels (R software GRM, http://wanglab.ucsd.edu/star/GRM/). I would suggest the readers such possibility that could largely benefit downstream analysis such as the detection of subpopulation of cells and cell trajectories, as they would mainly rely on biological variation. This would still be compatible with an assessment of HVG only based on biological variation by fitting the trend to the variance estimates of the endogenous genes (after technical denoising).

 9.    Authors state that the technical component estimation through the fitting of a mean-variance trend to the spike-in transcripts “is compromised by the small number of spike-in transcripts, the uneven distribution of their abundances and (for low numbers of cells) the imprecision of their variance estimates”. Do the same remarks generally apply to a spike-in-specific normalization? And if so, should spike-in normalization be considered accurate enough when applied to cases with strong DE even if it is conceptually more appropriate than a deconvolution approach?

 10.    In the section “Identifying HVGs from the normalized log-expression” the authors justify their choice of "the variance of the log-expression values because the log-transformation protects against genes with strong expression in only one or two cells. This ensures that the set of top HVGs is not dominated by genes with (mostly uninteresting) outlier expression patterns”. However, the filtering of genes with such patterns has already been proposed in a previous section, so those cases should no longer be a risk here.

 11.    The interpretability of the approach of "Identifying correlated gene pairs with Spearman’s rho" is to some extent limited without a previous analysis such as PCA, ICA or MDS, transforming the high-dimensional space into a space of independent (uncorrelated) dimensions. I would rather favor the identification of sets of genes with a high weight on each of the retained independent axis (i.e. driving the variance in such axes, and therefore disentangling sets of correlated genes for each of the orthogonal dimensions). Otherwise, the analysis could risk to be dominated by the first component, probably neglecting other relevant hidden factors.

 12.    In any case, I advise not to restrict to correlated HVGs downstream dimensionality reduction analysis such as PCA or ICA aiming at the identification of subpopulations of cells and their gene signatures. Such methods exploit correlation patterns (linear or non-linear) in a well-grounded way and they do not require a feature selection step. The sentence "We only use the correlated HVGs in plotPCA because any substructure should be most pronounced in the expression profiles of these genes" could eventually not hold true in some instances: correlated HVGs were assessed without considering those independent components, the relative contribution of each dimension to the total variance, and the relative contribution of each gene to each dimension.

 13.    In the brain dataset, correlated HVGs genes were assessed considering the design <- model.matrix(~sce$sex); correlatePairs(sce, design=design). It would be useful to further explain here how this function accounts for the design matrix on the assessment of Spearman’s rho.

 14.    In the brain dataset, removeBatchEffect from limma package is used to remove the sex effect. Then tSNE and PCA are applied on the sex-corrected expression values restricted to correlated HVGs. Consistently, correlated HVGs were assessed considering the very same factor: design <- model.matrix(~sce$sex); correlatePairs(sce, design=design). I would further warn the reader and stress the necessity of that consistency between both steps.

 15.    For consistency with the assessment of correlations based on Spearman's rho, in the heatmap I would recommend to assess first the dendrograms for the cells and the genes by using also a spearman correlation: For instance:

 cells.cor <- cor(expressionmatrix, method="spearman")

 cells.cor.dist <- as.dist(1-samples.cor)

 cells.tree <- hclust(cells.cor.dist,method='complete')

 And then in heatmap.2 setting Colv=as.dendrogram(cells.tree)

 And analogously with the genes for Rowv

 This should be adapted in the case that a design is used as in correlatePairs(sce, design=design)

 Personally I would also suggest to check how the heatmaps look by setting scale='row' in heatmap.2 function.

 16.    I would suggest explicitly mentioning in the pipeline which approaches are based on linear or non-linear assumptions. The workflow alternates methods from both categories, which should be taken into account to understand their downstream consequences. For instance:

- The function plotExplanatoryVariables from scater package, with the default method= "density", produces a density plot of R-squared values for each variable when fitted as the only explanatory variable in a linear model.
- The function removeBatchEffect from limma package fits a linear model to the data, including both batches and regular treatments, then removes the component due to the batch effects.
- Then the analysis is restricted to correlated HVGs, which are assessed on spearman’s rho, i.e. rank-based, non-linear
- tSNE is non-linear based, PCA is linear
- Clusters are defined through dynamic tree cut to the dendograms assessed by hierarchical clustering on the Euclidean distances between cells (linear, although in a non-orthogonal space)

17.    The use of hierarchical clustering for clustering cells into putative subpopulations is based on Euclidean distances (or correlations) assessed in a non-orthogonal space. I would rather favor an analytical clustering directly performed in a low-dimensional orthogonal space such us those led by PCA, ICA or MDS, in which the most-informative dimensions can be selected (e.g. through their eigen values in PCA).

 18.  A PCA analysis should be accompanied by a plot representing the % of variance explained by each principal component, so that it can be judged the number of relevant dimensions to be retained while disregarding the rest as “noise”. It could be the case that more than 2 dimensions are relevant to separate subpopulations in a finer detail. The inspection of eigenvalues would help supporting that "PCA plot is less effective at separating cells into many different clusters (Figure 24). This is because the first two principal components are driven by strong differences between specific subpopulations, which reduces the resolution of more subtle differences between some of the other subpopulations."

 19.    In the brain analysis, three main steps are: 1) the deconvolution method is used to normalize expression levels. Here similar cells are clustered together and cells are normalized in each cluster. Authors state: “This improves normalization accuracy by reducing the number of DE genes between cells in the same cluster”. Clustering is performed here with the quickCluster function from scran package, where a distance matrix is constructed using Spearman’s correlation on the counts between cells. 2) A hierarchical clustering is then performed and a dynamic tree cut is used to define clusters of cells. Then, the batch(sex)-corrected expression values of the (Spearman’s rho) correlated HVG are used to build a dendogram assessed through hierarchical clustering on the Euclidean distances between cells, where clusters are defined. And 3) those clusters are used to assess DE with edgeR on the counts, normalized using the library size-adjusted size factors (if I well understood) and including all genes (not only correlated HVG). I personally found such procedure a bit cumbersome as it is relying on different types of expression matrices and metrics in each of the 3 steps (see also next comment). I also wonder to what extent the initial quickCluster results could be biasing the clusters detected downstream, and, if so, whether the normalization step would be biasing in turn the differentially expression results. The correspondence between the quickCluster results with the clusters from the dendograms should at least be inspected and discussed.

 20.    In line with the previous comment, in the brain analysis I wonder whether the pipeline could somehow be simplified by 1) performing spike-in normalization (which seems possible given the quality of the spike-in trend observed in Figure 21), 2) doing a PCA on the batch(sex)-corrected expression values of all genes (not only correlated HVGs), and performing clustering on the retained principal components, and 3) assessing DE with edgeR on the counts normalized using the spike-in factors.

 Minor comments

 21.    Some code at the beginning of the analysis to check and install all the required packages would be welcome

 22.    Everything run smoothly in our hands except for the gdata package when trying to read the xls file. The perl command interpreter was running abnormally long and it was using a large amount of RAM. We finally opened the xls file in excel and converted into tab separated file, then read it using the general read.table command.

I have read this submission. I believe that I have an appropriate level of expertise to confirm that it is of an acceptable scientific standard, however I have significant reservations, as outlined above.

## References

[1] ShalekAKSatijaRShugaJTrombettaJJGennertDLuDChenPGertnerRSGaublommeJTYosefNSchwartzSFowlerBWeaverSWangJWangXDingRRaychowdhuryRFriedmanNHacohenNParkHMayAPRegevA: Single-cell RNA-seq reveals dynamic paracrine control of cellular variation. *Nature*.2014;510(7505) : 10.1038/nature13437 363-9 10.1038/nature13437 24919153PMC4193940

[2] BrenneckePAndersSKimJKKołodziejczykAAZhangXProserpioVBayingBBenesVTeichmannSAMarioniJCHeislerMG: Accounting for technical noise in single-cell RNA-seq experiments. *Nat Methods*.2013;10(11) : 10.1038/nmeth.2645 1093-5 10.1038/nmeth.2645 24056876

[3] GrünDKesterLvan OudenaardenA: Validation of noise models for single-cell transcriptomics. *Nat Methods*.2014;11(6) : 10.1038/nmeth.2930 637-40 10.1038/nmeth.2930 24747814

[4] IslamSZeiselAJoostSLa MannoGZajacPKasperMLönnerbergPLinnarssonS: Quantitative single-cell RNA-seq with unique molecular identifiers. *Nat Methods*.2014;11(2) : 10.1038/nmeth.2772 163-6 10.1038/nmeth.2772 24363023

[5] DingBZhengLZhuYLiNJiaHAiRWildbergAWangW: Normalization and noise reduction for single cell RNA-seq experiments. *Bioinformatics*.2015;31(13) : 10.1093/bioinformatics/btv122 2225-7 10.1093/bioinformatics/btv122 25717193PMC4481848

# Response

**Published:** 2016-10-26

**Authors:** Aaron Lun

**Affiliations:** Cancer Research UK Cambridge Research Institute, UK

Thanks for your comments, Antonio. Our responses are as below:

***1. In addition to the approaches proposed by the authors to detect low quality cells, I would suggest the readers the possibility of identifying outlier cells by performing a PCA on the normalized gene expression matrix restricted to protein-coding genes (e.g. using biotype annotations from Ensembl biomart). On the one hand, outlier cells will dominate the first principal components, which will show high percentage of variances simply accounting from the separation of the outlier from the compact cloud of “normal” cells. On the other hand, a PCA analysis could suggest keeping a cell whose relative similarity to the rest of the cells in a low-dimensional space seems rather normal, even if it could still have an allegedly "bad" quality metric.***

 This is certainly a valid approach, though we do not mention it here for several reasons. The first reason is that there is an increased risk of being confounded by biological effects when gene expression patterns are directly used, e.g. where uncommon cell types are classified as outliers and removed. The second is that we do not want to confuse readers with a variety of possible options - while our approach is not the only way to do it, it does work, and thus serves its purpose in this workflow. Finally, the use of PCA-based outlier detection has been explored in some detail by Ilicic *et al.* (2016), which we have already mentioned in the text.

***2. As an additional quality control check for the cells, I would also suggest to assess whether the sequencing depth was generally deep enough for most of the cells, by inspecting for instance the saturation curve of the number of detected genes (or other features like the known exon-exon junctions) as a function of the fraction of down-sampled reads.***

 This is an interesting idea, though it seems to be more useful as a diagnostic for future experiments rather than for an already existing dataset. Even if saturation is not reached, it would not affect the data analysis provided that the existing counts were large enough. Our diagnostics focus on the quality of the data that we currently have, rather than the potential for improving the experiment by collecting more data.

***3. In the text it is proposed to filter out low-abundance genes, defined as “those with an average count below a filter threshold of 1." However, the average count is assessed before the normalization step. Would it be more meaningful to apply this filter on the normalized counts?***

 Unfortunately, most normalization methods (e.g. deconvolution, TMM, DESeq) perform poorly with unfiltered data due to the poor precision of low counts. This necessitates some degree of filtering prior to normalization. We do not think that this has a major effect on the mean count for most genes, given that the size factors average out to unity across all cells.

***4. A priori it is difficult to rule out the possibility that the filtering of low-abundance genes could eventually hamper the identification of relevant genes in rare populations of cells. I would generally suggest being rather inclusive at this stage, especially when no clustering of single-cells has been done yet, so that it would still be possible to check whether e.g. the few cells expressing some genes -even if at low levels- are actually forming a distinctive and biologically relevant cluster.***

 In the context of this workflow, one of the roles of filtering is to reduce the number of genes that need to be tested as being highly variable. This improves power by reducing the severity of the multiple testing correction, increasing the chance that potentially informative genes are detected as HVGs and used in downstream analyses. Thus, while relaxing the filter may retain more genes, fewer of these genes may actually be used in the downstream analysis. (This is more likely than not - low-abundance genes are not generally detected as being highly variable, due to inherent limits on the scope of variability in count data.) Indeed, in the example of few cells expressing few genes at low levels, it is difficult to see how such genes would be detected as being significant in a HVG analysis.

***5. Authors propose as an alternative approach to gene filtering, to select genes that have non-zero counts in at least n cells. As illustrated in Figure 6, the number of cells expressing a given gene may be modeled by its mean expression level. This was elegantly addressed in Shalek et al. (2014) through a likelihood ratio test comparing a null model -where all cells express a gene in a lognormal fashion- with an alternate model -where a gene is not expressed in a subpopulation of cells α (See section "Controlling for relationship between expression level and detection efficiency" in the supplementary material: http://www.nature.com/nature/journal/v510/n7505/extref/nature13437-s1.pdf). Genes for which the null model is rejected may be indicative of a subpopulation of cells not expressing the gene at a higher fraction than the one expected from technical noise (e.g. dropout events). I would suggest exploring such approach in order to avoid filtering out relevant genes due to a sharp threshold on the number of cells expressing it.***

 There are several arguments against using such an approach, at least during the filtering stage. Firstly, this approach specifically selects for bimodal genes whereas it is entirely possible that interesting genes could vary across a continuum of expression values (or, in fact, are bimodal at two non-zero locations). The second is that the significance threshold effectively serves the same purpose as a threshold on the percentage of expressing genes - only less interpretable, as it depends on the vagaries and assumptions of the model. Indeed, default thresholds for significance (e.g. 1%, 5%) may not be appropriate for filtering and exploratory analyses. Thus, some tuning of the significance thresholds is likely to be required, further reducing interpretability. Consequently, we feel that the approach we have suggested is more likely to be generally useful to the wider biological community.

***6. In the section “Filtering out low-abundance genes”, the sentence "This provides some more protection against genes with outlier expression patterns, i.e., strong expression in only one or two cells. Such outliers are typically uninteresting as they can arise from amplification artifacts that are not replicable across cells.[...]" would be better followed by setting alt.keep <- numcells >= 2 instead of >= 10***

 The "ideal" threshold depends largely on the biological context. The HSC dataset contains a highly purified and homogeneous population. We would expect that most expressed genes would be present in a substantial number of these cells, hence the choice of threshold. While relaxing the filter is possible, this runs into the problems discussed above in our response to point 4. Of course, in other situations where rare cell types are present (e.g. olfactory neurons expressing unique receptors), relaxing the filter might be necessary to retain biological information. We have added a comment about this in the revised manuscript.

***7. It would be interesting to complement Figure 7 and 18 with a second panel representing the correlation between size factors from deconvolution versus spike-in-specific size factors, as done in Figure 27. In the eventual case that a low correlation between them was found in a non-DE scenario, would it be advisable to neglect spike-ins from the analysis?***

 We considered adding this, but felt that it would make this part of the workflow somewhat difficult to follow given that we use only the deconvolution factors for normalisation of the endogenous genes. Nevertheless, we agree that this is an important point and are glad that the reviewer pointed out Figure 27 where we discuss this issue in some detail.

 Low correlations between the spike-in and deconvolution size factors are not a cause for concern. As we have mentioned, this is entirely possible due to differences in total mRNA content. In terms of normalization, the two sets of size factors simply deal with different biases, so differences between them do not provide any indication of spike-in quality.

***8. As pointed by the authors, spike-in molecules have been extensively used to infer the amount of variability in the expression levels of one gene that can be explained from technical noise (e.g. Brennecke et al., 2013; Grün et al., 2014; Islam et al., 2014). Ding et al. (2015) went further on the applications of spike-in levels, by using them to explicitly remove technical noise and compute de-noised gene expression levels (R software GRM, http://wanglab.ucsd.edu/star/GRM/). I would suggest the readers such possibility that could largely benefit downstream analysis such as the detection of subpopulation of cells and cell trajectories, as they would mainly rely on biological variation. This would still be compatible with an assessment of HVG only based on biological variation by fitting the trend to the variance estimates of the endogenous genes (after technical denoising).***

 The GRM strategy is an interesting one. However, we do not use it here because the denoising is performed based on a curve fitted to the spike-in log-FPKMs against the known concentrations. This is philosophically similar to spike-in-based normalization, in that it will preserve information about total RNA content. For example, cells with more endogenous RNA will have larger gene counts and unchanged (or smaller) spike-in counts; this results in larger de-noised expression values compared to other cells with less total RNA. Such behaviour may not be desirable in situations where cell size is not of interest.

***9. Authors state that the technical component estimation through the fitting of a mean-variance trend to the spike-in transcripts “is compromised by the small number of spike-in transcripts, the uneven distribution of their abundances and (for low numbers of cells) the imprecision of their variance estimates”. Do the same remarks generally apply to a spike-in-specific normalization? And if so, should spike-in normalization be considered accurate enough when applied to cases with strong DE even if it is conceptually more appropriate than a deconvolution approach?***

 In general, no, the remarks do not apply for spike-in normalization. This is because spike-in normalization computes a single size factor, using information across all spike-in transcripts. As a result, the size factor is generally quite precise. Fitting of the mean-variance trend is less stable because it uses information from each individual spike-in transcript. This is subject to the issues described in the text, thus reducing the stability of the outcome.

***10. In the section “Identifying HVGs from the normalized log-expression” the authors justify their choice of "the variance of the log-expression values because the log-transformation protects against genes with strong expression in only one or two cells. This ensures that the set of top HVGs is not dominated by genes with (mostly uninteresting) outlier expression patterns”. However, the filtering of genes with such patterns has already been proposed in a previous section, so those cases should no longer be a risk here.***

 This depends on the type of abundance filtering that was chosen. In this workflow, we performed filtering based on the average count, which does not explicitly protect against strong outliers. Thus, some additional protection is needed during the downstream analysis. If filtering was performed based on an "at least n" strategy, then outliers will be less of an issue during HVG detection. Of course, the "at least n" filter has problems of its own regarding an appropriate choice for "n", as we have discussed in the text and in our response to point 6, which is why we have not used it as the default filtering strategy.

***11. The interpretability of the approach of "Identifying correlated gene pairs with Spearman’s rho" is to some extent limited without a previous analysis such as PCA, ICA or MDS, transforming the high-dimensional space into a space of independent (uncorrelated) dimensions. I would rather favor the identification of sets of genes with a high weight on each of the retained independent axis (i.e. driving the variance in such axes, and therefore disentangling sets of correlated genes for each of the orthogonal dimensions). Otherwise, the analysis could risk to be dominated by the first component, probably neglecting other relevant hidden factors.***

 This point of calculating these correlations is to provide a simple screen for genes that are likely to be involved in defining the substructure of the dataset. Interpretation of the cause of these correlations can then be performed using PCA, ICA, etc. as suggested on the subset of interesting genes. Without some pre-selection of genes (in terms of high variance or correlation), biological and technical noise may interfere with dimensionality reduction - see our response to point 12.

 Our approach allows relevant genes to be selected in a statistically rigorous manner based on significant correlations. In contrast, it is unclear how selection would be performed based on the PCA weights. For example, what should be considered a "high weight", and from how many principal components should genes be selected? The simplicity of the calculation of significant pairwise correlations also provides a useful sanity check for conclusions drawn from more complex downstream analyses.

 Finally, if there are hidden factors, these are likely to increase the correlations and cause rejection of the null hypothesis for the relevant genes. So, genes that are affected by these factors will still be retained for downstream analysis and interpretation.

***12. In any case, I advise not to restrict to correlated HVGs downstream dimensionality reduction analysis such as PCA or ICA aiming at the identification of subpopulations of cells and their gene signatures. Such methods exploit correlation patterns (linear or non-linear) in a well-grounded way and they do not require a feature selection step. The sentence "We only use the correlated HVGs in plotPCA because any substructure should be most pronounced in the expression profiles of these genes" could eventually not hold true in some instances: correlated HVGs were assessed without considering those independent components, the relative contribution of each dimension to the total variance, and the relative contribution of each gene to each dimension.***

 The aim of selecting correlated HVGs is to reduce the amount of technical and (uncorrelated/uninteresting) biological noise in the data to be used for downstream analyses. This improves the performance of dimensionality reduction approaches, especially if the substructure is relatively weak. For example, with PCA, adding a large number of uncorrelated genes will interfere with correct placement of cells along a trajectory:

*par(mfrow=c(1,2))*

*loc <- 1:100/100 # True placement of cells*

*a1 <- matrix(jitter(rep(loc, 50)), nrow=50, byrow=TRUE) # Correlated genes*

*x1 <- prcomp(t(a1))*

*plot(x1$x[,1]) # Should be on the diagonal*

*a2 <- rbind(a1, matrix(rnorm(100000), ncol=100)) # Adding uncorrelated noise*

*x2 <- prcomp(t(a2))*

*plot(x2$x[,1]) # Correct placing is disrupted*

 Similar arguments can be made for distance-based approaches like t-SNE and diffusion maps, where the nearest neighbours become more difficult to identify correctly with increasing noise.

 Finally, the identification of correlated HVGs does not need to consider the nature of the substructure. We only need to identify the genes that are affected by this substructure, in one way or the other - it is the function of downstream analyses to determine what the substructure actually represents.

***13. In the brain dataset, correlated HVGs genes were assessed considering the design <- model.matrix(~sce$sex); correlatePairs(sce, design=design). It would be useful to further explain here how this function accounts for the design matrix on the assessment of Spearman’s rho.***

 For one-way layouts, a value of rho is first computed within each group of cells. The average across all groups (weighted by the number of cells) is then used as the final value of rho for any given pair of genes. For more complex designs, a linear model is fitted to the log-normalized counts, and rho is calculated using the residuals of the model fit. (While the linear model approach also works for one-way layouts, it requires some additional assumptions that can be avoided with a simpler group-based approach.) More details can be found in the documentation for the correlatePairs() function.

***14. In the brain dataset, removeBatchEffect from limma package is used to remove the sex effect. Then tSNE and PCA are applied on the sex-corrected expression values restricted to correlated HVGs. Consistently, correlated HVGs were assessed considering the very same factor: design <- model.matrix(~sce$sex); correlatePairs(sce, design=design). I would further warn the reader and stress the necessity of that consistency between both steps.***

 We have added a comment on this to the manuscript.

***15. For consistency with the assessment of correlations based on Spearman's rho, in the heatmap I would recommend to assess first the dendrograms for the cells and the genes by using also a spearman correlation...***

 Our dendrograms are constructed based on the distances between cells, which is different from the correlations between genes. Using the correlations to cluster the genes makes more sense with respect to checking consistency, but the primary aim of our analysis is to identify clusters of cells (potential subpopulations) rather than clusters of genes. The latter is certainly a worthwhile analysis (e.g. to identify gene modules) but, in the context of this workflow, the differences in clustering on correlations versus Euclidean distances would only have a minor effect.

***16. I would suggest explicitly mentioning in the pipeline which approaches are based on linear or non-linear assumptions. The workflow alternates methods from both categories, which should be taken into account to understand their downstream consequences...***

 Obviously, each computational method makes a number of assumptions. For the sake of readability and simplicity (especially for inexperienced readers), we have not discussed most of these assumptions in this workflow, except for those that are critical to choosing between methods, e.g. spike-in normalization versus deconvolution. Nonetheless, we have modified the manuscript to elaborate on the reasons for using non-linear methods such as Spearman's rho and t-SNE.

***17. The use of hierarchical clustering for clustering cells into putative subpopulations is based on Euclidean distances (or correlations) assessed in a non-orthogonal space. I would rather favor an analytical clustering directly performed in a low-dimensional orthogonal space such us those led by PCA, ICA or MDS, in which the most-informative dimensions can be selected (e.g. through their eigen values in PCA).***

 There are many possible approaches to clustering, each with their own advantages and disadvantages. For example, pre-selection of a low-dimensional space via PCA may reduce noise during clustering, but it may also discard subtle features present in lower-ranked PCs. Our clustering approach is simple but effective enough, which is why we have used it in this workflow. Other methods may well do better, but a discussion of the pros and cons of different clustering strategies is beyond the scope of this article.

***18. A PCA analysis should be accompanied by a plot representing the % of variance explained by each principal component, so that it can be judged the number of relevant dimensions to be retained while disregarding the rest as “noise”. It could be the case that more than 2 dimensions are relevant to separate subpopulations in a finer detail. The inspection of eigenvalues would help supporting that "PCA plot is less effective at separating cells into many different clusters (Figure 24). This is because the first two principal components are driven by strong differences between specific subpopulations, which reduces the resolution of more subtle differences between some of the other subpopulations."***

 We only use PCA for visualization, rather than selection of principal components for further quantitative analysis in low-dimensional space. For this purpose, knowing the relative contributions to the total variance from non-visualized components is less helpful. For example, even if we determined that the top 10 dimensions were "relevant", it is unclear how this would assist visualization. Nonetheless, we now mention in the text how this information can be generated and used.

***19. In the brain analysis, three main steps are: 1) the deconvolution method is used to normalize expression levels. Here similar cells are clustered together and cells are normalized in each cluster. Authors state: “This improves normalization accuracy by reducing the number of DE genes between cells in the same cluster”. Clustering is performed here with the quickCluster function from scran package, where a distance matrix is constructed using Spearman’s correlation on the counts between cells. 2) A hierarchical clustering is then performed and a dynamic tree cut is used to define clusters of cells. Then, the batch(sex)-corrected expression values of the (Spearman’s rho) correlated HVG are used to build a dendogram assessed through hierarchical clustering on the Euclidean distances between cells, where clusters are defined. And 3) those clusters are used to assess DE with edgeR on the counts, normalized using the library size-adjusted size factors (if I well understood) and including all genes (not only correlated HVG). I personally found such procedure a bit cumbersome as it is relying on different types of expression matrices and metrics in each of the 3 steps (see also next comment). I also wonder to what extent the initial quickCluster results could be biasing the clusters detected downstream, and, if so, whether the normalization step would be biasing in turn the differentially expression results. The correspondence between the quickCluster results with the clusters from the dendograms should at least be inspected and discussed.***

 In terms of the choice of matrices and metrics, we have chosen approaches that we feel are suitable for each step of the workflow. Given that each step examines a different aspect of the data, some flexibility is inevitably required in supplying the correct input to each method.

 Regarding quickCluster, Lun *et al.* (2016) show that unbiased size factor estimates are still obtained after clustering. This is because size factors computed within each cluster are explicitly corrected to be comparable between clusters. As for the similarity between the quickCluster results and the dendrograms, we do not believe that this provides a useful indication of method performance. Some agreement is expected, as the two methods should recover similar structure in the data. However, some disagreement is also expected, as quickCluster provides a quick-and-dirty clustering to reduce the amount of DE genes present during deconvolution, while the dendrograms are much more refined due to feature selection. Such incongruences are not a problem for normalization - even if quickCluster identifies the "incorrect" clusters, it is still adequate if it separates cells with vastly different transcriptomic profiles.

***20. In line with the previous comment, in the brain analysis I wonder whether the pipeline could somehow be simplified by 1) performing spike-in normalization (which seems possible given the quality of the spike-in trend observed in Figure 21), 2) doing a PCA on the batch(sex)-corrected expression values of all genes (not only correlated HVGs), and performing clustering on the retained principal components, and 3) assessing DE with edgeR on the counts normalized using the spike-in factors.***

 One could certainly perform such an analysis. However, we chose to use the approach described in the workflow, because feature selection can improve the results of downstream analyses, as discussed in our response to point 12; and the choice of whether or not to do spike-in normalization depends primarily on whether total RNA content is interesting, not on the quality of the spike-ins.

***21. Some code at the beginning of the analysis to check and install all the required packages would be welcome***

 We have added a link to the Bioconductor workflow page, which provides instructions for installing all required packages and running the workflow.

***22. Everything run smoothly in our hands except for the gdata package when trying to read the xls file. The perl command interpreter was running abnormally long and it was using a large amount of RAM. We finally opened the xls file in excel and converted into tab separated file, then read it using the general read.table command.***

 We understand the suboptimality of dealing with Excel files in bioinformatics analysis. Unfortunately, the authors of this study provided the count data in Excel format on NCBI GEO. We decided to load the data directly rather than manually supplying the counts in a simpler format. The latter would make the workflow less generalisable as it would no longer use data from public, well-recognised sources. In our hands, loading of the Excel file usually requires a couple of minutes and 3-4 GB of RAM.

# Peer review: Referee response for version 1

**DOI:** 10.5256/f1000research.10234.r15991

**Published:** 2016-10-18

**Authors:** David duVerle

**Affiliations:** 1 Department of Computational Biology and Medical Sciences, University of Tokyo, Tokyo, Japan

The pipeline described in this article seems promising. I was able to partly reproduce the results, as well as run similar treatment on a single cell dataset of my own.

 However:

- The fundamental flaws mentioned by other reviewers over a month ago, still haven't been addressed: the pipeline requires Dev versions of R and bioconductor packages, yet makes no mention of it anywhere in the article.
- In fact, even after installing the Bioconductor Dev versions of all required modules, it would appear the pipeline no longer works with the latest versions (e.g. scran_1.1.10, with R 3.3.1):

*> isSpike(sce) <- "ERCC"*

Error in `isSpike<-`(`*tmp*`, value = "Spike") :

*'isSpike' must be logical or NULL*

**

*etc.*

 While likely easy to fix, this type of incompatibility issues undermine the entire point of the article and perfectly illustrate the dangers of relying on development versions for this type of pipeline.

- Additionally, the example dataset used by the article, is loaded from an Excel spreadsheet, which is generally considered extremely bad practice. It would behoove the authors of a software walkthrough aimed at somewhat-novice bioinformaticians to encourage best practices.
- In the current conditions, and until some **major revision** work is done, it is impossible to properly review the pipeline and approve this article unreservedly.

I have read this submission. I believe that I have an appropriate level of expertise to confirm that it is of an acceptable scientific standard, however I have significant reservations, as outlined above.

## References

[1] ZeebergBRRissJKaneDWBusseyKJUchioELinehanWMBarrettJCWeinsteinJN: Mistaken identifiers: gene name errors can be introduced inadvertently when using Excel in bioinformatics. *BMC Bioinformatics*.2004;5: 10.1186/1471-2105-5-80 80 10.1186/1471-2105-5-80 15214961PMC459209

# Response

**Published:** 2016-10-18

**Authors:** Aaron Lun

**Affiliations:** Cancer Research UK Cambridge Research Institute, UK

Thanks for your comments, David. Regarding the incompatibility in software versions, we have been waiting for the imminent release of the latest version of Bioconductor (3.4) before revising the article. It seemed more prudent to wait for the latest software to become available, rather than making stop-gap modifications to accommodate soon-to-be-obsolete versions. We believe that this update should clear up any problems with execution of the workflow.

 We agree that Excel spreadsheets are a poor formatting choice for bioinformatics work. Unfortunately, the processed dataset is provided in this format from NCBI GEO (https://www.ncbi.nlm.nih.gov/geo/query/acc.cgi?acc=GSE61533, see Supplementary files). While having to tackle Excel formatting is not ideal, it is preferable to having to re-process the entire dataset to obtain counts from the raw read sequences. Moreover, at no point do we save into Excel - analysis results are always stored in simple tab-delimited formats, and the R objects themselves are saved in serialized form.

# Peer review: Referee response for version 1

**DOI:** 10.5256/f1000research.10234.r16243

**Published:** 2016-09-30

**Authors:** Andrew McDavid

**Affiliations:** 1 Department of Biostatistics and Computational Biology, University of Rochester, Rochester, NY, USA

Lun, McCarthy and Marioni share a workflow for analysis of single cell RNAseq (scRNA-seq) data using software they have developed. The workflow is illustrated on two data sets of varying size and characteristic. The computational and statistical findings of the workflow are interpreted in their experimental context. Having a well-documented protocol for the analysis of scRNA-seq is an important contribution to the community, since it is still a wilderness in terms of methods and processing, for better or worse.That scRNA-seq is a quickly evolving discipline--and the implications this has for the workflow--forms the bulk of my criticism of this paper.

**1a.** The paper describes a currently-unreleased version of software. Other reviewers have indicated the difficulties this poses. I trust the authors will verify the correctness of their code and reproducibility of the analysis when their packages are finalized in Bioconductor 3.4. I also trust that this workflow will be made available as a literate (e.g knitr) document so that readers won't have to cut and paste from their web-browser. This reviewer was able to reproduce the figures reported in the first data set after loading the development version of ` *scater*` (now version 1.1.14).

**1b.** The main software package ` *scater*` defines a `SCESet` inheriting from `ExpressionSet`, which has been superceded by `SummarizedExperiment`. SummarizedExperiment is more likely to scale to large data sets (it can store data out of core or in sparse matrix formats). In practice, this is not such a big deal since it's relatively easy to coerce between the two object types.

**2.**  The title of this article stipulates that it is for "low-level" analysis of RNA-seq data, but the all-important question of how to process the data as many analysts will get them (short reads as .fasta files) is elided.  

 (Pseudo)-Alignment and quantification is an important, and probably overlooked step in scRNA-seq analysis. Counting transcripts by counting overlaps with features, a la countOverlaps or htSeq is inefficient ^1^, since many reads (30%-80% of those that map anywhere, in this reviewer's experience) do not align uniquely. Hence the need and value to use quantification tools that respect the degeneracy of multimapping reads, ie, RSEM, Star, Kallisto, Sailfish, *et al*.  A low-level analysis thus may wish to consider remapping with an appropriate tool. Fortunately, it does appear that ` *scater*` has provisions for doing (re)-alignment with Kallisto.

**3.**  The authors may consider referencing other extant methods that could address areas of their workflow, especially methods that are adapted to deal with the non-normality of scRNA-seq data.

- For normalization, there is scone, which tests many different normalization procedures and optimizes over the "best" one.
- For identification of highly variable genes, there is Basics ^2^, which applies a hierarchical Bayesian model to test for over-dispersion, as opposed to modeling departures from an overall mean-variance relationship.
- For single cell differential expression and gene set enrichment for bimodal distributions found in scRNA-seq, there is MAST ^3^.
- For clustering, there is clusterExperiment.
- For multi-dimensional scaling on bimodal data, there is ZIFA ^4^. All of the above, aside from ZIFA are R/Bioconductor packages.

I have read this submission. I believe that I have an appropriate level of expertise to confirm that it is of an acceptable scientific standard, however I have significant reservations, as outlined above.

## References

[1] LiBDeweyCN: RSEM: accurate transcript quantification from RNA-Seq data with or without a reference genome. *BMC Bioinformatics*.2011;12: 10.1186/1471-2105-12-323 323 10.1186/1471-2105-12-323 21816040PMC3163565

[2] VallejosCAMarioniJCRichardsonS: BASiCS: Bayesian Analysis of Single-Cell Sequencing Data. *PLoS Comput Biol*.2015;11(6) : 10.1371/journal.pcbi.1004333 e1004333 10.1371/journal.pcbi.1004333 26107944PMC4480965

[3] FinakGMcDavidAYajimaMDengJGersukVShalekAKSlichterCKMillerHWMcElrathMJPrlicMLinsleyPSGottardoR: MAST: a flexible statistical framework for assessing transcriptional changes and characterizing heterogeneity in single-cell RNA sequencing data. *Genome Biol*.2015;16: 10.1186/s13059-015-0844-5 278 10.1186/s13059-015-0844-5 26653891PMC4676162

[4] PiersonEYauC: ZIFA: Dimensionality reduction for zero-inflated single-cell gene expression analysis. *Genome Biol*.2015;16: 10.1186/s13059-015-0805-z 241 10.1186/s13059-015-0805-z 26527291PMC4630968

# Response

**Published:** 2016-10-18

**Authors:** Aaron Lun

**Affiliations:** Cancer Research UK Cambridge Research Institute, UK

Thanks for your comments, Andrew. Our responses are as below.

***1a. The paper describes a currently-unreleased version of software. Other reviewers have indicated the difficulties this poses. I trust the authors will verify the correctness of their code and reproducibility of the analysis when their packages are finalized in Bioconductor 3.4. I also trust that this workflow will be made available as a literate (e.g knitr) document so that readers won't have to cut and paste from their web-browser. This reviewer was able to reproduce the figures reported in the first data set after loading the development version of `scater` (now version 1.1.14).***

 Yes, this was an oversight on our part. The revised verison will include a link to the Bioconductor workflow page, where users can simply run a command to automatically download the relevant data files and packages prior to running the workflow.

***1b. The main software package `scater` defines a `SCESet` inheriting from `ExpressionSet`, which has been superceded by `SummarizedExperiment`. SummarizedExperiment is more likely to scale to large data sets (it can store data out of core or in sparse matrix formats). In practice, this is not such a big deal since it's relatively easy to coerce between the two object types.***

 We considered the practicality of storing data in sparse matrix format. Unfortunately, most existing tools for downstream data analysis require a full-sized matrix as input, so any gains in memory efficiency during storage seem to be countered by the need to (repeatedly) expand the matrix at multiple analysis steps. Moreover, a sparse matrix only improves efficiency for raw count data where unambiguous zeroes are present; upon applying normalization and transformation steps, this may no longer be the case, such that a full-sized matrix will ultimately be required anyway.

***2.  The title of this article stipulates that it is for "low-level" analysis of RNA-seq data, but the all-important question of how to process the data as many analysts will get them (short reads as .fasta files) is elided. (Pseudo)-Alignment and quantification is an important, and probably overlooked step in scRNA-seq analysis. Counting transcripts by counting overlaps with features, a la countOverlaps or htSeq is inefficient1, since many reads (30%-80% of those that map anywhere, in this reviewer's experience) do not align uniquely. Hence the need and value to use quantification tools that respect the degeneracy of multimapping reads, ie, RSEM, Star, Kallisto, Sailfish, et al.  A low-level analysis thus may wish to consider remapping with an appropriate tool. Fortunately, it does appear that `scater` has provisions for doing (re)-alignment with Kallisto.***

 We find that conventional feature counting works quite well for read-based scRNA-seq data, having used this approach in several recent studies (Achim *et al.*, 2015; Kolodziejczyk *et al.*, 2015; Scialdone *et al*., 2016). While ignoring multi-mapped reads during quantification is conservative, we feel that it does provide a greater degree of confidence in our downstream inferences. Certainly, there may be gains in power from using tools that extract more information from multi-mapping reads, but we do not consider this advantage to be so pronounced that it should be standard procedure for all scRNA-seq data analyses. For UMI-based data, there does not yet appear to be any clear "gold standard" approach for UMI processing into counts, so we have not provided any description of that step.

 In summary, we decided to start the workflow from the raw count data, rather than starting from read sequences, as conventional approaches for quantification described elsewhere seem to work well; to maintain some flexibility with respect to future developments in this field; and because our workflow focuses on the steps of the analysis that are carried out in R/Bioconductor, whereas most existing quantification tools require manual installation and execution from the command-line.

**References:**

- Achim *et al.* (2015), *Nature Biotechnology* 33:503–509
- Kolodziejczyk *et al.* (2015), *Cell Stem Cell* 17(4):471-485
- Scialdone *et al. *(2016), *Nature* 535:289-293

**The authors may consider referencing other extant methods that could address areas of their workflow, especially methods that are adapted to deal with the non-normality of scRNA-seq data.**

 As you have stated, there are many alternative approaches that could be used in various parts of the workflow. However, we feel that it is beyond the scope of this article to enter into discussions about the relative advantages of different methods. In fact, this may undermine the pedagogical value of the workflow by providing too many options to inexperienced users. The methods we have described work well in a variety of situations, so we have chosen them for use in the various analysis steps. We have added a sentence to the discussion about the existence of alternative methods for low-level processing, and encouraged experienced users to explore them.

# Peer review: Referee response for version 1

**DOI:** 10.5256/f1000research.10234.r15987

**Published:** 2016-09-19

**Authors:** Hongkai Ji

**Affiliations:** 1 Department of Biostatistics, Johns Hopkins Bloomberg School of Public Health, Baltimore, MD, USA

In this article, the authors introduce a computational workflow to perform low-level analysis of single-cell RNA-seq (scRNA-seq) data based on R and Bioconductor. The workflow takes a read count matrix as input, and it provides R commands for loading data, quality control, gene filtering, data normalization (with or without spike-in controls), classifying cells based on their cell cycle phase, identifying highly variable genes, analyzing genes’ pairwise correlation, and basic data exploration such as clustering and visualization. The workflow is demonstrated using a number of real data examples. Overall, I think that the workflow provides a timely and very useful guide for people who want to analyze scRNA-seq data.

 This study is largely reproducible. I am able to obtain all major results in this article by running the commands provided by the authors. I have several comments and suggestions which I hope the authors can address in order to make their workflow more user-friendly.

 1. It seems that installing the right version of R and Bioconductor is crucial for this pipeline to work. Some commands in the workflow depend on R version 3.3.1 or higher and the developing (devel) version of Bioconductor. The first time I tried the workflow, I encountered numerous errors. For example,

 > isSpike(sce) <- "ERCC"

 Error in `isSpike<-`(`*tmp*`, value = "ERCC") :

   'isSpike' must be logical or NULL

 > numcells <- nexprs(sce, byrow=TRUE)

 Error: could not find function "nexprs"

 > sce <- computeSpikeFactors(sce, type="ERCC", general.use=FALSE)

 Error in .local(x, ...) :

   unused arguments (type = "ERCC", general.use = FALSE)

 It turns out that I used an older version of R and Bioconductor. I then updated my R and Bioconductor packages and still had many problems. Finally, I decided to completely remove R and Bioconductor from my computer. I then installed R 3.3.1 and Bioconductor (devel version), and the pipeline worked. Although I eventually fixed the problem, I feel that this trial and error process can be frustrating for users. I therefore suggest that the authors make the R/Bioconductor dependencies clear at the beginning of the article. It would be even better if the authors could minimize the pipeline’s dependency on certain versions of R/Bioconductor.

 2. This workflow uses a number of R and Bioconductor packages. A user may not have all packages installed on their computer. Installing these packages one by one manually can be a little tedious. It would be nice if the authors can provide an R script that automatically finds missing packages on a user’s computer and install them. This could improve the pipeline’s user experience.

 3. It will also be useful if the authors can provide an R file that contains all commands in the workflow so that users only need to slightly edit their code for future datasets. It might be beyond the scope of this article, but the authors may consider delivering the pipeline using an R shiny graphical user interface in the future to make it accessible to users without R coding experience.

I have read this submission. I believe that I have an appropriate level of expertise to confirm that it is of an acceptable scientific standard, however I have significant reservations, as outlined above.

# Response

**Published:** 2016-09-19

**Authors:** Aaron Lun

**Affiliations:** Cancer Research UK Cambridge Research Institute, UK

Thanks for your comments, Hongkai. Our responses to each of your points is below:

1. Yes, this was an oversight on our part. The pipeline was developed using packages from BioC-devel, to take advantage of cutting-edge methods in each package. For that reason, the pipeline is strictly dependent on Bioconductor release version 3.4, a fact that we will make explicit in the next revision. We do not think that this is a major inconvenience given that the next release of Bioconductor is less than a month away.
2. This is a good point. In fact, this article would ideally coincide with a parallel release on the Bioconductor workflow page, where the workflow installation machinery will automatically install all dependencies required for the package. Unfortunately, because this article was using packages from BioC-devel, we were unable to coordinate its release with that on the Bioconductor workflow page (which is limited to BioC-release packages). This will be fixed in the next revision where we will add a reference to Bioconductor-based installation of required packages.
3. While we understand the convenience that an R script can offer, we feel that supplying such a script would invite attempts to blindly use the code without considering the context or caveats of the various methods. We believe that some initial copy-pasting is a small price to pay if the user is consistently reminded of how to properly interpret the output. Note that the Bioconductor workflow site and our Github page provide an Rmarkdown file containing all the necessary code blocks for easy execution of the entire workflow; if necessary, users can change the input files to generate an analysis report similar to the article. Of course, a graphical user interface is even more intuitive, but this is difficult to set up in a manner that is amenable to rigorous and reproducible data analysis.

# Peer review: Referee response for version 1

**DOI:** 10.5256/f1000research.10234.r15990

**Published:** 2016-09-09

**Authors:** Diana H.P. Low

**Affiliations:** 1 Institute of Molecular and Cell Biology, Agency for Science, Technology and Research, Singapore, Singapore

Lun and colleagues describe a low-level analysis specific for single-cell RNA-seq experiments, using open-source packages available on Bioconductor. This paper could potentially be a valuable resource for those who want to carry out such analysis in R.

 The steps are very descriptive, and they even include 2 different datasets presenting different types and conditions for analysis. They have done a very thorough job in explaining the decisions taken at each step of QC, filtering, normalization and provide some basic but important visualization examples (clustering, heatmaps) that would help in assessing not only the quality of the dataset technically, but also provided information on the outcome of the experiment itself.

 Unfortunately I could not run some of the steps in the workflow which prevented me from assessing the code. Some I could figure out and "fix" in the attempt to run the code, but others not so much.

 I provide some (not exhaustive) examples below to help in the troubleshooting, and if these (and the subsequent code relying on these outputts) could be solved, I would be happy to continue the review further.

1. isSpike(sce) <- "ERCC" //worked with isSpike(sce) <- is.spike
2. can't find the function nexprs [I had to use numcells <- rowSums(exprs(sce)!=0)]
3. is.ercc <- isSpike(sce, type="ERCC") //worked with [is.ercc <- isSpike(sce)
4. Could not run code from the section: Identifying HVGs from the normalized log-expression

var.fit <- trendVar(sce, trend="loess", use.spikes=FALSE, span=0.2)

 Error in simpleLoess(y, x, w, span, degree = degree, parametric = parametric,  : 

   invalid 'x'

I have read this submission. I believe that I have an appropriate level of expertise to confirm that it is of an acceptable scientific standard, however I have significant reservations, as outlined above.

# Response

**Published:** 2016-09-09

**Authors:** Aaron Lun

**Affiliations:** Cancer Research UK Cambridge Research Institute, UK

Thanks for your comments, Diana. The code actually depends on Bioconductor version 3.4 (i.e., BioC "devel"), rather than the current Bioconductor 3.3 (i.e., BioC "release"). This allows us to include cutting-edge features from all packages to provide a high level of functionality in the workflow. However, some of these features are not present in the release version, thus leading to execution failure.

 The devel versions of all packages can be easily installed by setting *useDevel() *followed by *biocLite()*, as described on the Bioconductor website. We will also modify the text to explicitly state that Bioconductor 3.4 is required - currently, this can only be implicitly determined from the package versions, which admittedly is not obvious to casual users.
